# Self-reported social media use does not affect cross-cultural consensus in first impressions

**DOI:** 10.1017/ehs.2026.10042

**Published:** 2026-03-24

**Authors:** Vojtěch Fiala, Slawomir Wacewicz, Zuzana Štěrbová, Ondřej Pavlovič, Juan David Leongómez, Andrés Castellanos-Chacón, Selahattin Adil Saribay, Karel Kleisner, Petr Tureček

**Affiliations:** 1 Centre for Language Evolution Studies, Faculty of Humanities, Nicolaus Copernicus University in Toruń, Toruń, Poland; 2 Institute of Advanced Studies, Nicolaus Copernicus University in Toruń, Toruń, Poland; 3 Department of Philosophy and History of Science, Faculty of Science, Charles University, Prague, Czech Republic; 4 Department of Psychology, Faculty of Arts, Charles University, Prague, Czech Republic; 5 EvoCo: Human Behaviour and Evolution Lab, Faculty of Psychology, Universidad El Bosque, Bogotá, Colombia; 6 Department of Psychology, Kadir Has University, Istanbul, Türkiye; 7 Center for Theoretical Study, Charles University and Czech Academy of Sciences, Prague, Czech Republic

**Keywords:** cross-cultural agreement, facial characteristics, WEIRD people, geometric morphometrics

## Abstract

Research focusing on first impression formation based on facial stimuli lacks a conclusion on whether there is a cross-cultural agreement and how deeply it has proliferated across distant populations. Social media may play an important role in the level of cross-cultural agreement as they provide us with overwhelming numbers of visual stimuli, including faces. Sharing social media aesthetics, their users may utilise facial cues congruently. We asked participants from seven distant, ethnically variable countries from five continents to rate facial attractiveness, trustworthiness and dominance of a single ethnically invariant facial sample (*N* = 195, 106 women, M_Age = 23.23), also accounting for their self-reported social media use intensity and socioeconomic background. We expected the agreement between cultures to be better for participants who reported a higher intensity of social media use. Instead, we observed substantial cross-cultural agreement, especially for attractiveness and trustworthiness, regardless of the self-reported social media use intensity. However, the samples of participants from similar cultural backgrounds (same countries) agreed more. We also see substantial agreement in facial cue utilisation. In line with previous research, the distinctiveness of facial shape affects perceived attractiveness congruently across cultures. Despite the relatively small age range, age positively affects ascribed dominance.

## Graphical abstract

**Figure:**
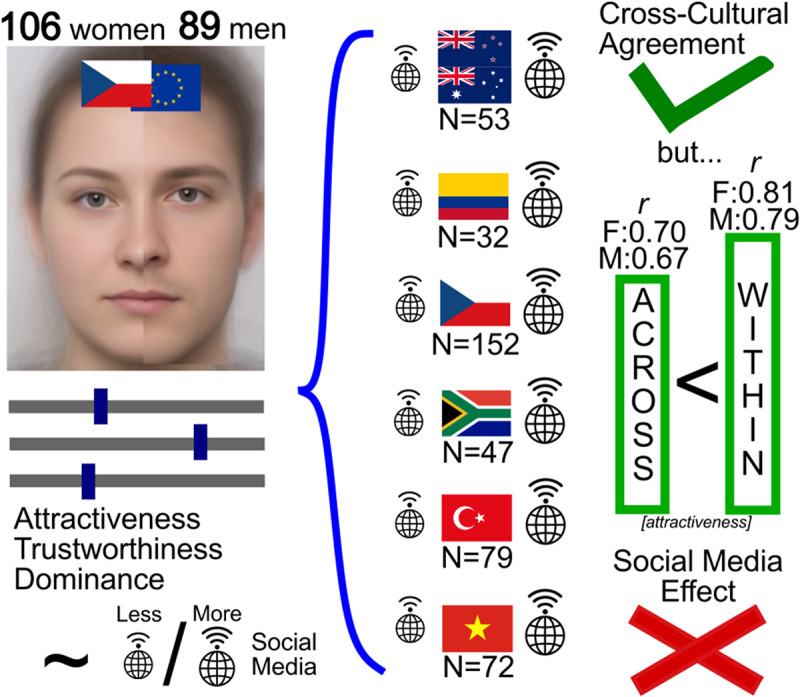

## Social media summary

You, readers of this status, your social media use intensity does not systematically affect how you perceive faces.

## 1. Introduction

Research on facial characterisation continues to fall short of a consensus on several of its core questions. As an example, let us consider whether people across countries, populations, or social classes evaluate facial features consistently during the *first impression formation* from neutral facial photographs (as defined in Zebrowitz, 2017). Previous studies reported that raters from different world regions did agree on the perceived attractiveness of the same set of faces (Coetzee et al., 2014) and that raters in every studied culture prefer similar facial characteristics, like averageness/prototypicality (Kleisner et al., 2024).

Additional studies indicate that shared attractiveness norms and visual diet are important for agreement across visually distinctive populations (Sorokowski, Kościński, et al., 2013) and that unfamiliarity across populations hinders appropriate utilisation of attractiveness-relevant traits like facial shape averageness (Apicella et al., 2007) or skin tone (Strom et al., 2012). Moreover, individuals fine-tune their preferences for facial characteristics to align with local socioeconomic conditions and their individual life history (De Barra et al., 2013; Scott et al., 2008), which further shapes their first impressions. For example, in harsh environments with bad health and unequally distributed resources, people shall prefer individuals with better immune systems or resource provisioning capacity (Pereira et al., 2020; Saribay et al., 2021).

Resource provisioning capacity and immunocompetence are associated with certain facial phenotypes, typically with more pronounced sex-typical traits. Research has examined how preferences for sex-typical traits vary across populations facing different public health conditions (DeBruine et al., 2011) or different levels of income inequality (Brooks et al., 2011). The studies revealed profound contrasts between cultures: both income inequality and worse public health resulted in more pronounced preferences for more masculine (i.e., more sex-typical) traits in male faces. These results were treated as evidence that variance in environmental characteristics induces adaptive cross-cultural differences in forming first impressions and preferences. However, these studies were criticised for using artificially manipulated facial stimuli (Fiala et al., 2021; Rennels et al., 2008). Additionally, state-level measures may not capture the individual’s lived environment well (Borras-Guevara et al., 2017, 2019) and can even mislead a cross-country comparison as some of them depend on the country’s population size (Kratochvíl & Havlíček, 2024).

Furthermore, the effect of environmental characteristics on facial preferences may be quite complex: An experimental priming study by Saribay et al. (2021) showed both a drop and an increase in facial masculinity preferences under different types of environmental harshness, accompanied by an overall decline in attractiveness ratings with high perceived pathogen prevalence. This complexity potentially explains why more masculine traits preference was found both in ‘lower health nations’ (DeBruine et al., 2011; Marcinkowska et al., 2014) and in modern, large-scale urban settings (Marcinkowska et al., 2019; Scott et al., 2014). Importantly, the effects reported by DeBruine et al. (2011) and Scott et al. (2014) point in opposite directions, possibly because the former relied solely on online participants from large-scale societies, whereas the latter used a more diverse sample, both in terms of stimuli and large – vs. small-scale societies, with ratings collected offline.

Furthermore, some studies in evolutionary psychology have begun to question the general concept of ‘shared preferences’ across different cultures (Zhan et al., 2021) and at the inter-individual level (Ibáñez-Berganza et al., 2019). The role of subjectivity in facial characterisation, which has been downgraded since the rise of the current evolutionary psychology paradigm (Langlois et al., 2000), is hereby reintroduced. After years of research, the question of whether individuals from different world regions agree on the attribution of facial characteristics remains unanswered, as it is still unclear which traits are consistently preferred and what societal, demographic, personal, or environmental factors drive or hinder this agreement.

Notably, the local environment (such as level of socioeconomic development, resource distribution, and pathogen prevalence) is not the sole explanation of dis/agreement in first impression formation among populations; mutual knowledge is also important. Agreement across populations may be influenced by experience with the facial morphologies of the foreign populations (Sorokowski, Sorokowska, et al., 2013; Strom et al., 2012; cf Pavlovič et al., 2021). By this reasoning, we should observe greater agreement between individuals who have larger experience with faces of various foreign populations. They might have learned who is considered attractive in each foreign population, for example, via the mechanism of social learning (Little et al., 2011). Notably, first-hand experience with foreign faces (i.e., face-to-face interaction with foreigners) is not strictly required for such knowledge, which may instead come via other channels, such as movies, TV shows, or other cultural artefacts (Coetzee & Perrett, 2011), or via group-based cultural stereotypes (Saribay et al., 2025).

During the period of intensive studies of facial first impression formation (1990s-onwards), the world has been undergoing significant changes when it comes to *shared environments* and *visual diet*. The estimated fraction of the global population with internet access doubled between 2011 and 2021, and is now estimated to be above 60 per cent (World Bank, 2025b). Moreover, since the early 2010s, we have been experiencing a rapid proliferation of social media (SM) apps (Ortiz-Ospina, 2019), streamlined by the mass production of relatively accessible smartphones and establishing modern standards of wireless internet access via mobile networks.

Social media establishes a unique environment shared among its users (boyd & Ellison, 2007). Hypothetically, global access to shared virtual environments could help bridge the gaps in visual experiences. This would allow everyone to encounter a wide range of invariant, shared content. Such environments could standardise labels for stimuli, categorising them as ‘appealing’, ‘interesting’, ‘overwhelming’, ‘offensive’, and ‘socially unacceptable’ invariantly for every user. We propose that how individuals perceive the intensity of their online social media usage should reflect their actual usage intensity and their adherence to social media visual norms. Such a social media use intensity (*sensu lato*) should, in turn, affect the level of agreement in first impression formation across populations (cf. Holland & Tiggemann, 2016). In other words, if there is a time when we can observe a broad cross-cultural consensus on how first facial impressions are formed, it should be now. Additionally, if there is a factor that could balance out the differences in these impressions, it would likely be exposure to social media.

There is some evidence that mass media affects mate preferences (Becker, 2004; Boothroyd et al., 2020). With the introduction of the mass media in remote non-WEIRD societies, locals began to acquire ‘global’ (‘Western’ *sensu* Boothroyd et al., 2020) norms of bodily attractiveness. Online social media (social networking sites, hereinafter SM) may present a rich source of facial stimuli from various ethnicities. The prevailing *visual diet*, which affects the social perception of faces (Singh et al., 2022), encompasses a wide range of individuals and includes a diverse array of ethnicities. With the rising quality of AI-generated faces (Boudníková & Kleisner, 2024) and artificial manipulation of photos through filters (Mendoza, 2022; Ramphul & Mejias, 2018), these representations may increasingly diverge from the natural variation found in human appearance.

There is also indirect evidence suggesting that the most popular social media personalities tend to showcase highly attractive members of their populations (Eggerstedt et al., 2020). Further, these personalities appear to form a common cluster, a ‘population of social media influencers’ who share similar facial features across different populations (Fiala et al., 2025). Moreover, the audience of well-known social media personalities is international: According to a database of Instagram influencers’ profiles, taken from hypeauditor.com (01-04/2024), in some countries (India, United States, Brazil, Turkey), up to 40% of the most popular influencers (*N* = 1000) are from outside the selected country. As a result, social media users around the world are exposed to a similar range of facial cues, which may shape their preferences in similar ways. Preliminary evidence shows that users’ facial preferences change due to SM exposure (Sun, 2023). Despite its relatively short existence, SM has also already revolutionised plastic surgery (Eldaly & Mashaly, 2022), with clients asking surgeons, ‘make me look like I was using this Instagram filter’. Therefore, while these phenomena await further inspection, SM might be creating its own environment, making user preferences more similar across different regions. Yet, its impact could be marginal if local contexts and everyday offline visual exposure remain the dominant influences.

### 1.1. The current study

Due to the widespread use of online social media, finding an ‘uncontacted one’ in modern large-scale populations would be demanding. However, whether the *intensity* of social media use accounts for differences in the attributed characteristics remains a valid question. Social media affects people in varying ways, especially between individuals who practice self-control and those who habitually scroll. For convenience, we focus on participants for whom internet access is commonplace. These individuals have become ‘inhabitants of the digital society’ but vary in their online social media use intensity. The visual diet of those who just habitually scroll, arguably, differs from the ones who are more deeply engaged in the ‘offline world’, since facial configurations of popular social media creators, too, differ from the general population (Eggerstedt et al., 2020; Fiala et al., 2025).

While less precise than objective tracking methods (Scharkow, 2016), self-reported social media use may better capture participants’ subjective awareness of their online engagement. This awareness is central to our research question, as facial preferences depend not only on exposure but also on recognising and internalising online aesthetics; self-reports, therefore, provide a useful proxy for perceived engagement, with stronger agreement expected among heavier users.

To disentangle the combined effect of SM, socioeconomic background, and ethnic/cultural factors on ascribing facial characteristics, we collected first impression ratings of the same sample of faces (Czechs) from local (Czech) raters and from raters outside Central Europe. We asked participants about the intensity of their social media use (Boer et al., 2022), including questions regarding which social media platforms they use and how intensively they use them, as well as inquiries about their behaviour in the online world in general. Prior to data collection, we specified our research question here: https://aspredicted.org/4L8_RSH.

We expected that the ratings from frequent social media users worldwide would align more closely with each other, rather than with the ratings from less frequent social media users. Alternatively, the local circumstances and distinctive visual diet present factors that outweigh the effects of SM, rendering the population identity a strong predictor of ascribed characteristics, regardless of whether this is ultimately a consequence of evolutionary adaptive processes. We also considered morphometric predictors related to facial shape, skin lightness, and age, and explored whether they consistently affect ascribed characteristics across populations. A recent study suggests that processing of different aspects of facial morphology may be culture-dependent (Pokorný et al., 2024). By adding facial morphology and skin lightness, we explore whether they predict perceived traits differently in groups of more and less intensive social media users.

In addition to participants’ ethnicity, we focused on their country of residence, as members of the same population are likely to share similar first impressions shaped by common sources of visual information (Cook et al., 2022). This allows us to capture how local ‘visual diets’ influence facial perception, which should be especially pronounced among non-intensive users, whose preferences are more affected by offline environments.

## 2. Materials and methods

### 2.1. Facial photo acquisition

The stimuli sample consisted of 195 individual frontal facial photos of Czech citizens (European/Caucasian, ‘White’), mostly (though not exclusively) university students: 106 women (mean age ± SD: 23.09 ± 4.12, range: 19–39) and 89 men (mean age ± SD: 23.38 ± 4.25, range: 19–43). This type of stimuli sample (age-matched, ethnically homogenous) enabled studying the effects of social media use intensity on cross-cultural agreement better than an ethnically diverse sample would (only one group, the Czechs, should perceive the stimuli faces as ingroup faces, cf. Kleisner et al., 2019). The participants were recruited by direct invitations, social networks, and flyers displayed on university campuses. Participants were informed about the purpose of the data collection and instructed to remove facial cosmetics, jewellery, and any other decorations. They wore standardised black T-shirts and were asked to adopt a neutral facial expression. As compensation, participants received either CZK 200 (app. 7 €) or a bottle of wine. One session took place in 2016 at the Faculty of Science, while the other, in 2019, was at the Faculty of Physical Education and Sport, Charles University, Prague.

Portrait images were captured using a Canon EOS 6D full-frame DSLR. It was set to an exposure time of 1/100, an ISO speed of 100, and a focal length of 85 mm. Before each session, a standard colour chart was shot for later colour adjustment, and the F-number alignment between the camera and a flash metre was checked. The subjects were positioned inside a photographic tent, which is essentially a cube with its interior surfaces – the background, sidewalls, ceiling, and floor – made from white fabric. Illumination was provided by a Photon Europe MSN HSS-800Ws light source, equipped with a 120 cm reverse lighting umbrella. The resulting images were stored in RAW camera format, a resolution of 5472 × 3648 pixels, and were subsequently colour corrected in Adobe Photoshop Lightroom, cropped, resized to ca 400 × 600 pixels, and saved as JPEGS.

### 2.2. Rating collection, social media use intensity assessment

**Figure 1.**
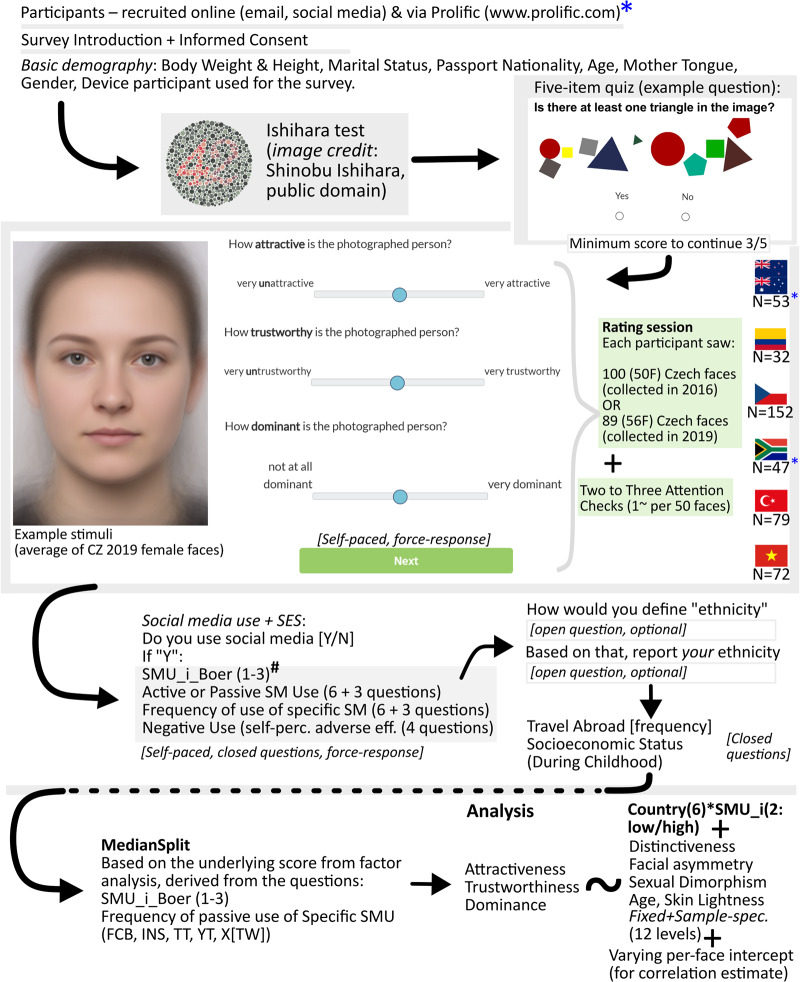
Example stimuli and schematic overview of the experimental design. ^#^SMU_i_Boer (1-3) refers to three questions excerpted from Boer et al. (2022) and Van Den Eijnden et al. (2018). The questions are printed in full in Section 2.2.2.

The next step was to collect facial rating data in Czechia and distant countries. Subsequently, the participants were categorised as frequent or infrequent social media users based on self-reported social media use intensity (split by median, see Section 2.3 and Fig. 1). Please note that this study uses the terms intensity and frequency interchangeably. Following Boer et al. (2022) we decided to keep the term ‘social media use intensity’ they introduced; however, in certain contexts, especially when talking about samples of participants, we decided to refer to ‘frequent’ and ‘infrequent’ users’ samples. In principle, the questions listed below measure frequency (number of visits to social media per constant period), which can be considered a measure of how intensively SM is used by the participants.

#### Facial ratings

We created the survey using the experiment builder Labvanced (https://labvanced.com/). The study was initially constructed in English (VF, SW) and distributed in Australia, New Zealand, and South Africa via Prolific. It was subsequently translated into Czech, Spanish, Vietnamese, and Turkish by native speakers (VF/ZS for the Czech version, JDL for the Spanish version, and SAS for the Turkish one). Since all the translators are English speakers and co-authors of this study, they were aware of the research hypothesis, and they denoted changes that could potentially alter the meaning and affect the results. These instances were discussed and corrected.

The Vietnamese version was translated by our non-academic Vietnamese collaborator, who is a native Vietnamese speaker and is also fluent in English. To ensure accuracy, the Vietnamese version was back-translated using ChatGPT 4 and Google Translate (08/2024). Any potential differences in meaning were discussed with the Vietnamese collaborator and addressed appropriately.

Initially, we informed participants about the purpose of the study, obtained their informed consent, and asked them to report basic demographic information, including age, weight, height, gender, and the device they use to complete the survey. Subsequently, they took a simple quiz that involved recognising objects in pictures. Those who passed the quiz proceeded to the rating session.

Faces appeared on the screen one at a time in random order. The rating scales ranged from 0 (not at all attractive/trustworthy/dominant) to 100 (very attractive/trustworthy/dominant). Afterwards, we asked detailed questions regarding the participants’ life history. This included queries about the travel abroad frequency, ranging from multiple times a year to never. We also asked about their family’s socioeconomic situation during childhood, the participants’ ethnicity, their mother tongue, and their passport nationality. This section also contained questions about social media use.

In Australia, New Zealand, and South Africa, participants were acquired via Prolific (obligatorily online, only registered users may take part). In Turkey, most of the participants were enrolled in university courses. In the rest of the countries, the participants were recruited by sharing the link via email and social media posts (snowball method). All the responses were collected online from the participants’ own device (a Windows/Macintosh/Linux computer (*N* = 240/74/6), an Android (54) or iOS (52) smartphone, or a tablet (9); Table S3 in online Supplementary Materials).

To get a more complex picture of the participants’ nationality, ethnic origin, and language background, see Table S2 in the online Supplementary Materials. The vast majority of participants had citizenship of the country they were sampled and were located there at the time of survey completion. Some samples exhibited greater ethnic diversity, such as Australia, where participants reported European and South-East Asian origins with equal frequency, and Colombia, where participants often identified as mixed or ‘Mestizo’. In contrast, other samples were more ethnically homogeneous, like Czechia and Turkey. In Vietnam, the majority of participants either left the question of ethnicity deliberately unanswered or identified with universal categories: ‘Asian’ or simply ‘human race’.

#### Social media use intensity

Our hypothesis posits that frequent (intensive) users of social media experience different effects on their facial perception compared to infrequent (non-intensive) users due to different prevailing visual diets. We were primarily interested in the frequency (intensity) of passive use (watching) of global social media platforms that have been popular since the early 2020s. Nonetheless, recognising that the relationship between the frequency (intensity) of social media use and facial perception may vary by platform, we inquired more broadly both about general and platform-specific social media usage.

We first asked three questions derived from the social media use intensity (SMU-i) scale (Boer et al., 2022; Van Den Eijnden et al., 2018):

SMU-i #1: How often PER DAY do you look at social network sites? (for example, Facebook, *X[*Twitter*]*, Instagram, *YouTube*, or *TikTok*)

SMU-i #2: How often A WEEK do you post, photo or video on social network sites? (for example, Facebook, *X[*Twitter*]*, Instagram, *YouTube*, or *TikTok*) [the comma between ‘post’ and ‘photo’ was copied by mistake]

SMU-i #3: How often A WEEK you ‘like’ your posts, photos or videos of others on social network sites? (e.g., Facebook, *X[*Twitter*]*, Instagram, *YouTube*, or *TikTok*)

The parts in *italics* denote changes to the original scale from Boer et al. (2022) and Van Den Eijnden et al. (2018). One of the originally listed social media platforms, Google +, has been discontinued, while another one (Twitter) has been renamed (X). We also included two media that focus on videos: YouTube and TikTok. YouTube videos and YouTube shorts were not distinguished (cf. Violot et al., 2024). Each question was an ordered Likert scale, anchored by 0 = ‘Never or less than once a week/day’ and 7 = ‘More than 40 times’.

Subsequently, we asked how frequently participants used each of the *selected social media* (Facebook, YouTube, Instagram, TikTok, X[Twitter]), plus one locally popular SM one by one (six questions). The six-point score was anchored as ‘I do not use that’ to ‘Frequently during the day’.

We also asked whether users use the listed social media actively (uploading content), semi-actively (commenting on and reacting to content uploaded by others), or passively (only watching the content), plus the option ‘I don’t use it.’

In addition to the media listed above, participants were allowed to list three other social media platforms in open questions and asked to indicate how frequently they use them and whether they do so passively or actively. Self-reported social media use varied between participants, with neither being mentioned by more than 20% of the participants. Therefore, we excluded these questions from further analyses.

Four final questions (‘negative use’) traced the participant’s attitudes towards using social media and whether they were aware of any adverse effects of social media on their life (‘I spend a lot of time on online social media’, ‘I’m losing interest in other hobbies because of social media’, ‘I prefer following people and events on social media to interactions outside of it’, ‘If for some reason I can’t visit social media, I feel like I’m missing out’).

Throughout the study, participants were allowed to skip most questions on social media use or discontinue their participation by closing the survey if they felt uncomfortable or no longer wished to continue.

### 2.3. Ascribing participants to a group based on social media usage

#### Categorising participants into groups

We categorised participants into Frequent and Infrequent users based on how they were positioned on a scale of social media use frequency and intensity (SMU-score). To get the score, we used factor analysis, details of which are reported in Part II of the ‘Supplementary_Materials.pdf’ document. Briefly, we identified the underlying score, which was loaded by the three questions taken from Boer et al. (2022) and three out of five questions on specific social media use frequency (all loadings > 0.4). We did the factor analysis using functions fa.parallel() and fa() from the R package psych (Revelle, 2024).

The Elbow method supported a one-factor or two-factor solution (Figures S1–S2). While the parallel analysis run on the polychoric correlation matrix suggested two-to-four underlying factors, the inspection of the 2–4 factor solutions showed that the additional factors were each defined by a single item with no coherent simple structure. Following standard retention criteria (≥2 salient loadings per factor, content coherence, and reliability), we retained a parsimonious structure: a single SMU intensity factor for scoring (SMU-score).

Furthermore, when we included the four ‘negative use’ questions in the factor analysis, a two-factor model with the SMU-score and the Negative Effects Score appeared. Notably, the somewhat ambiguous question ‘I spend a lot of time on online social media’ (originally listed among ‘negative use’ questions), loaded (>0.4) both the SMU-score and Negative Effects Score.

Regardless of the exact layout, the first factors from every analysis, which were steadily loaded by the question on frequency/intensity of use of selected social media, correlated by 0.86 (Table S6). Their correlation with the Negative Effects Score was still positive, but substantially lower (∼0.6). We did not include the questions asking about active/passive use in the analysis, as they happened to combine a binary choice (use/not use) with the graded usage intensity, limited to just three ordered levels (passive/semi-active/active). However, scores based on this question correlated positively with both the Negative Effects Score and the SMU-score. Participants were subsequently split into two groups, based on the SMU-score (within-country median split). We also ran the analyses with participants within a 25% band around the median of the SMU-score excluded (see Part IV of the Supplementary Material). We also divided the raters’ sample based on the underlying score of the frequency of using three social media which specialises on visual content (YouTube, TikTok, and Instagram).

#### Exclusion criteria and other checks

We excluded participants who did not pass a simple initial quiz, those who did not pass attention checks, and those who did not proceed with the survey at some point. We also checked visually whether participants did not mark each face with the same number. Suspicious cases were further inspected on whether they assigned the same number to more than 90% of the stimuli. No such occurrence was observed; however, a few participants (*N* < 10) used quite invariant ratings in a single group/scale (e.g., trustworthiness/females) combination. We decided to keep these participants in the dataset, as they could lower rather than raise the cross-sample agreement.

We also ran a simplified analysis in which we divided the participants based on (a) the frequency of travelling abroad, (b) the family’s financial situation during childhood. Results of these analyses are available in the online ‘Supplementary_Materials.pdf, Part V’. These analyses corroborate our conclusions as reported below.

### 2.4. Analyses

#### Model description

The rating data (see Table 1 for details) were entered in a tidy data table (long format, 42,420 rows). We treated the rated scales (A/attr = attractiveness, T/trustw = trustworthiness, and D/dom = dominance) as the primary response variables. The scores were standardised prior to the analysis. This standardisation was done on the pooled sample to ensure that the eventual scale-use differences between different samples were preserved. All three scales were entered into the same model and treated as correlated (Ma et al., 2015). The model utilised a three-dimensional multivariate Gaussian distribution, forming a multivariate linear model:

**Table 1.** Basic description of the sample of the participants (raters)

Culture	Number of participants	Of it females/males^1^	Age [Yrs]	Distribution procedure
Australia + New Zeal.	53	28/24	34.51 ± 12.62	Prolific (online)
Colombia	32	19/13	30.12 ± 12.95	Online – social media (link shared publicly), email – peers (snowball)
Czech Republic	152	119/32	35.94 ± 10.13	Online – social media (publicly shared link, email (snowball)
South Africa	47	32 /15	25.62 ± 4.28	Prolific (online)
Turkey	79	63/15	22.15 ± 3.03	Online – via email to university course attendants
Vietnam	72	42/28	26.36 ± 7.46	Online (email, social media apps) – distributed by local collaborator (snowball)

*Notes*:

1 Please note that a small fraction of participants decided not to report their gender.

c(Attractiveness, Trustworthiness, Dominance) ∼ multi_normal(c(mu_attr, mu_trustw, mu_dom), Rho, sigma)

Where *mu_attr, mu_trustw,* and *mu_dom* stand for expected mean values of the three response variables. Rho is a matrix of residual correlations between the scales, and sigma characterises the joint variance of the three-dimensional multinormal distribution.

The three formulas that estimated the effects of rater group and morphometric predictors on the facial ratings (one for attractiveness, one for trustworthiness, and one for dominance) can be transcribed as:

*mu_attr/mu_trustw/mu_dom <- a + f_per_group_pr[FSMUi,1])*

+ (beta_age + f_per_group_pr[FSMUi,2])*age

+ (beta_dist + f_per_group_pr[FSMUi,3])*distinctiveness

+ (beta_asym + f_per_group_pr[FSMUi,4])*asymmetry

+ (beta_sshd + f_per_group_pr[FSMUi,5])*sexual_shape_dimorphism

+ (beta_L + f_per_group_pr[FSMUi,6])*Lightness

+ **f_per_group [face,FSMUi]** + z_rNV[rater] * sigma_rater

‘a’ is the fixed intercept across all the groups (it is expected to approach zero).

The term ‘f_per_group_pr[FSMUi,1]’ estimates the effect of a group on ascribed ratings (‘intercept’).

The terms ‘f_per_group_pr[FSMUi,2-6]’ allow us to estimate the effect of a group on the five anthropometric/morphometric predictors’ effects. These variables were always included in the analysis. Evidence suggests that the processing of facial features (typically shape vs. colour, Pokorný et al., 2024) is culture-dependent.

FSMUi: There were 12 groups (six world regions, with participants from each region divided into two subgroups based on their frequency/intensity of social media use). Mind that each term was fitted as formula-specific (‘a’ above represents a_A for attractiveness, a_T for trustworthiness, a_D for dominance, and so forth).

‘Beta’-s are fixed slopes for the five morphometric and demographic predictors.

Priors for the fixed terms were normally distributed around zero mean, with standard deviation = 0.5 (a) or 0.3 (betas). The term f_per_group_pr[FSMUi,1-6] was subject to non-centred parameterisation and sampled using partial pooling, whose principle is identical to what is described just below.

The term in **bold** (‘f_per_group[face,FSMUi]’) stood for the matrix of face-level varying effect [face] in each group of the raters [FSMUi], and it was modelled as correlated with Cholesky decomposition. The Cholesky factor (L_Rho_FSMUi) of the correlation matrix was estimated using LKJ Cholesky distribution with shape parameter prior set to 2. For the Cholesky decomposition, a matrix of z-scores (z_FSMUi) was estimated, using standard normal distribution, and the vector of standard deviations (sigma_FSMUi) was estimated using exponential distribution with rate parameter 1. Together, these terms established the 12 vectors of varying terms for the faces’ ratings in the 12 groups:

*transpars> matrix[face, 12]:f_per_group <- compose_noncentered(sigma_FSMUi, L_Rho_FSMUi, z_FSMUi)*.

This allowed us to estimate the correlation between the 12 groups’ ratings (*Rho_FSMUi*):

*gq> matrix[12,12]:Rho_FSMUi <<- Chol_to_Corr(L_Rho_FSMUi)*

The last part of the equation (z_rNV[rater] * sigma_rater) was a hierarchical term with non-centred parameterisation, using an exponential hyperprior on sigma_rater (rate = 1). It served to estimate the rater-level random effects. The varying intercepts per rater were subsequently computed as:

a_rNV_rater = a + z_rNV * sigma_rater

We also fitted models that omitted random terms at the level of a face (the **bold** part of the equation above) and compared the models’ out-of-sample predictive accuracy (using the Widely Applicable Information Criterion). The ulam() function in the package rethinking, which we used to fit the models, does not return log likelihood when the model has more than one response variable. Therefore, the model comparison was conducted on models with either attractiveness, trustworthiness, or dominance as the sole response variable. As this comparison clearly supported the models with varying per-face terms, we proceeded to fit the model with the three response variables. Importantly, the models with one or three response variables returned similar predictions for the response variable(s) contained.

We used Bayesian inference to evaluate the joint posterior distribution of the parameters within regression models. The models were fitted using R with the rethinking package (McElreath, 2020). It uses the interface of cmdstanr (Gabry et al., 2025) to introduce Stan’s (Stan Development Team, 2020) Markov Chain Monte Carlo (MCMC) infrastructure for sampling the posterior. We used 14 chains, each with 700 iterations, and performed parallel sampling on a 16-core Intel i9 CPU (see the note in the online ‘Supplementary_Materials.pdf’, Figure S5).

#### Differences from the pre-registered analysis

We added five linear predictors in the above-described analysis: Age, Distinctiveness (a morphometric measure of distance of the face from the mean value of the population, Kleisner et al., 2024), Sexual Shape Dimorphism (a variable expressing the position of a face on the axis connecting male and female average configuration, Kleisner et al., 2021), facial asymmetry (expressing the difference between the two mirrored versions of the stimuli, Kleisner et al., 2024), and Lightness (skin lightness measured as CIELab L* from the facial skin of the stimuli).

The models were fitted separately for the male and female subsamples of the stimuli to account for the anticipated sex-dependent effect of the morphometric predictors. We decided not to estimate the sex-specific effects in a common model, as the model structure was already complex and computationally demanding.

As a small modification relative to the preregistration, we excluded a ‘control group’ (i.e., previously acquired rating data from a large Czech sample). This is because these data were collected before the hypotheses were established, and a different rating scale was used for Dominance (1–7 Likert scale). However, an interested reader is advised to visit the project’s repository at https://github.com/VojtechFiala/YUFE_Rating_study_1, a document titled ‘YUFE_Rating_Study_Old_Analyses.pdf’ includes analyses that follow the original preregistration protocol strictly (in accordance with the recent guide on pre-registration, Lakens, 2024).

##### Analyses with raters scoring close to the median excluded

We also ran the analysis excluding participants whose social media intensity scores fell within the 25% band around the median for at least two of the three social media use intensity measures. The results are reported in the online document ‘Supplementary_materials.pdf’, page 23-onwards.

#### Interpreting results

We extracted 4,900 samples from the joint posterior distributions following the sampling. To perform the post-hoc comparison of mean estimates for different groups and correlations between the groups, we took advantage of the generative nature of Bayesian models. As the model returned distributions of credible posterior parameter values, one could detail the predictions by simple arithmetic operations done on the selected estimated parameters’ distributions.

### 2.5. Ethics statement

The authors assert that all procedures contributing to this work comply with the ethical standards of the relevant national and institutional committees on human experimentation and with the Helsinki Declaration of 1975, as revised in 2008. All procedures mentioned and followed were approved by the Institutional Review Board of the Faculty of Science of Charles University (protocol ref. number 04/2020 and 10/2024, and the Committee for Ethics of Scientific Research at the Institute of Psychology at Nicolaus Copernicus University 29/2024. The data collection in Colombia was approved by the local Institutional Review Bureau.

## 3. Results

Unless otherwise stated, we report the results based on a model that includes attractiveness, trustworthiness, and dominance as dependent variables, and morphometric measures, age, and skin lightness as predictors. For alternative parameterisations, see ‘Supplementary_Materials.pdf’.

In general, raters from the same country agreed more with each other than with raters from a different country, and social media use had no systematic effects. As for the morphometric predictors, distinctiveness and age were stable negative predictors of attractiveness, and the effects of anthropometric and morphometric predictors were also mostly stable across cultures.

### 3.1. Correlations between raters’ samples

For *women’s attractiveness*, we observed that the correlation within a country was, on average, by 0.13 [*95% percentile-based compatibility interval* is 0.07, 0.18] higher than the correlation between countries (Table 2). In contrast, social media use frequency had no effect on the correlation strength (frequent users’ samples tended to correlate by −0.02 [−0.10; 0.07] less closely with each other than did the infrequent users’ samples).

**Table 2.** Correlation comparisons (per-face). Mean stands for ‘mean correlation’

Female stimuli	Attractiveness	Trustworthiness	Dominance
Mean within cultures (a)	0.81[0.76; 0.86]	0.70 [0.61; 0.77]	0.61 [0.51; 0.70]
Mean across cultures (b)	0.69 [0.63; 0.74]	0.63 [0.56; 0.70]	0.48 [0.40; 0.55]
*(a–b) Difference*	*0.13 [0.07; 0.18]*	*0.06 [*−*0.01; 0.14]*	*0.13 [0.04; 0.22]*
Mean frequent users (c)	0.68 [0.60; 0.74]	0.64 [0.55; 0.72]	0.64 [0.55; 0.72]
Mean infrequent users (d)	0.70 [0.62; 0.77]	0.64 [0.55; 0.72]	0.53 [0.43; 0.63]
*(c–d) Difference*	−*0.02 [*−*0.10; 0.07]*	*0.00 [*−*0.11; 0.11]*	*0.11 [*−*0.02; 0.24]*
Male stimuli	Attractiveness	Trustworthiness	Dominance
Mean within cultures (e)	0.79 [0.72; 0.84]	0.69 [0.61; 0.77]	0.69 [0.60; 0.77]
Mean across cultures (f)	0.67 [0.60; 0.73]	0.57 [0.50; 0.64]	0.53 [0.45; 0.60]
*(e–f) Difference*	*0.12 [0.06; 0.18]*	*0.12 [0.04; 0.20]*	*0.16 [0.07; 0.25]*
Mean frequent users (g)	0.67 [0.59; 0.74]	0.52 [0.43; 0.62]	0.51 [0.41; 0.60]
Mean infrequent users (h)	0.68 [0.60; 0.76]	0.65 [0.57; 0.73]	0.55 [0.45; 0.65]
*(g–h) Difference*	−*0.03 [*−*0.10; 0.08]*	−*0.13 [*−*0.24;* − *0.01]*	−*0.04 [*−*0.17; 0.08]*

For *women’s trustworthiness*, the correlations in within-culture ratings were not credibly different between the groups; on average, within-culture ratings were by 0.06 [−0.01; 0.14] higher than between-culture ratings (Table 2). No systematic differences were observed between frequent and infrequent social media users (0.00 [−0.11; 0.11]. As for *Women’s dominance*, the within-culture correlations were on average higher by 0.13 [0.04; 0.22], and the frequent users’ samples, too, agreed with each other on average more compared to the average correlation among infrequent users’ samples (0.11 [−0.02; 0.24]).

**Figure 2.**
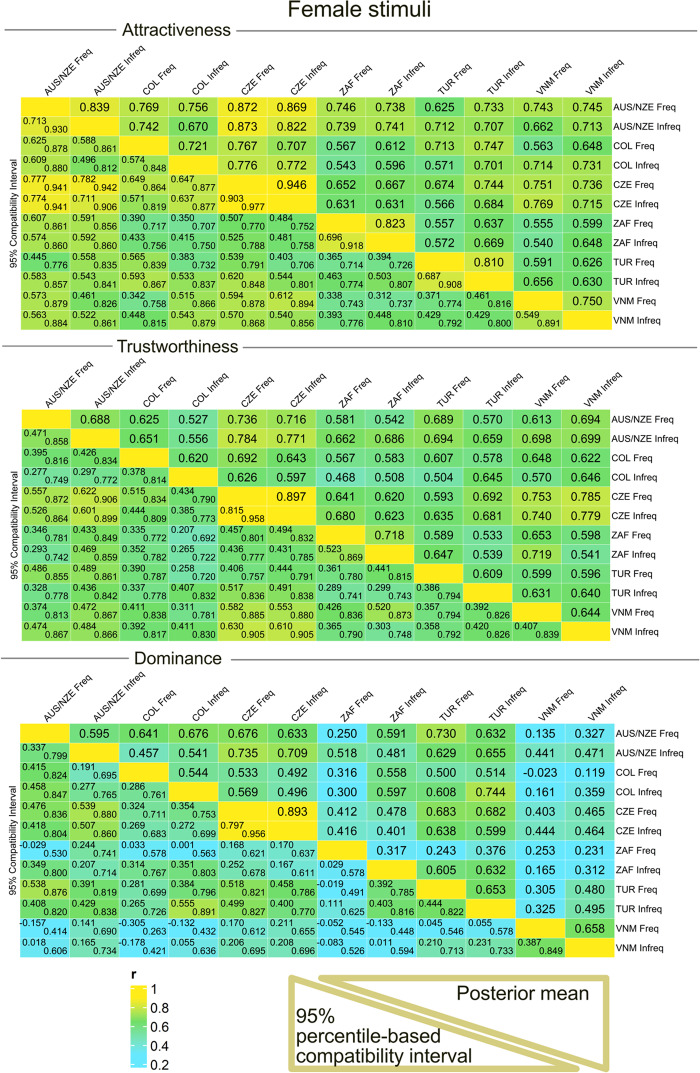
Posterior distributions of correlation coefficients: association between ratings in different samples, *female stimuli* (Freq = users above median in social media use frequency [‘frequent users’]; Infreq = users below the median [‘infrequent users’]). Country abbreviations (CZE [Czechia], AUS/NZE [Australia/New Zealand], TUR [Turkey], COL [Colombia], VNM [Vietnam], ZAF [South Africa]) correspond to three-letter country codes (ISO 3166-1 alpha-3). This plot was created using the package ComplexHeatmap (Gu, 2022; Gu et al., 2016).

As shown in Figure 2, between-culture agreements, expressed as correlations, were moderate to high for attractiveness and trustworthiness. However, the agreement was lower for dominance scale, and Vietnamese raters did not agree with the rest of the cultures on perceived dominance above chance.

In *men’s attractiveness*, the correlation within a country was on average by 0.12 [0.06; 0.18] higher than across countries, and no role in the cross-sample agreement could be assigned to social media use frequency. For *men’s trustworthiness*, the correlations within samples from the same country were on average by 0.12 higher [0.04; 0.20] than the correlations across samples. Frequent users agreed on trustworthiness by 0.13 less [−0.24; −0.01], compared to infrequent users. In *men’s dominance*, too, within-country correlations were by 0.16 [0.07; 0.25] higher than between-country. Unlike in women, the difference in average correlation across frequent and infrequent users was close to zero (Table 2).

**Figure 3.**
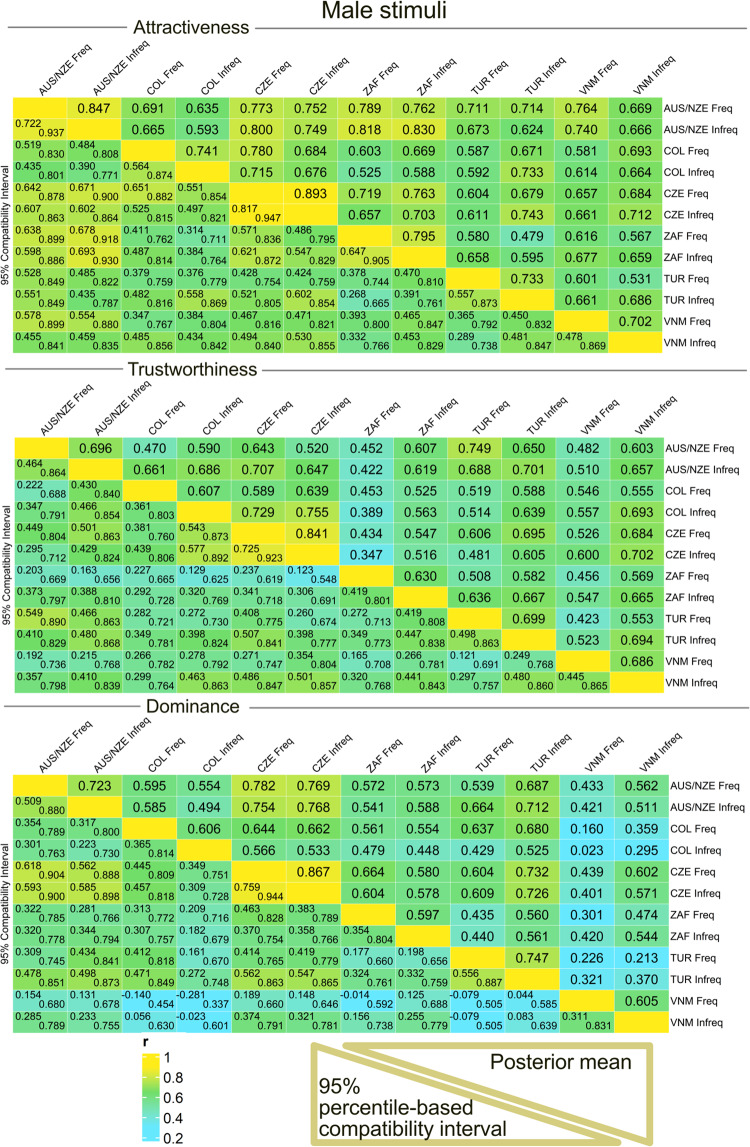
Posterior distributions of correlation coefficients: association between ratings in different samples, *male stimuli*. Abbreviations and layout are the same as in Figure 2.

As in women, there was strong to moderate cross-cultural agreement on attractiveness and trustworthiness (>0.7 and >0.3, respectively). Although mostly credibly positive, the correlations were weaker for male dominance, especially regarding the Vietnamese raters (Fig. 3).

Additional comparisons (see Table S4 in Supplementary Materials) marked that the correlation between the two Czech samples was conclusively higher than was the average of the other correlations and the same applied to the comparison of average of correlations in a combined sample of Czech and Australian/New Zealander ratings vs. the rest of the samples. Samples collected from Prolific did not align more closely with each other than how they aligned with the non-Prolific samples, except for male dominance.

The models also estimated correlation between the three scales on the level of individual ratings. Resulting mean estimated correlations for different scales (between 0.37 and −0.03) marked that the agreement is generally low on that level.

### 3.2. Mean estimates for different raters’ samples

**Figure 4.**
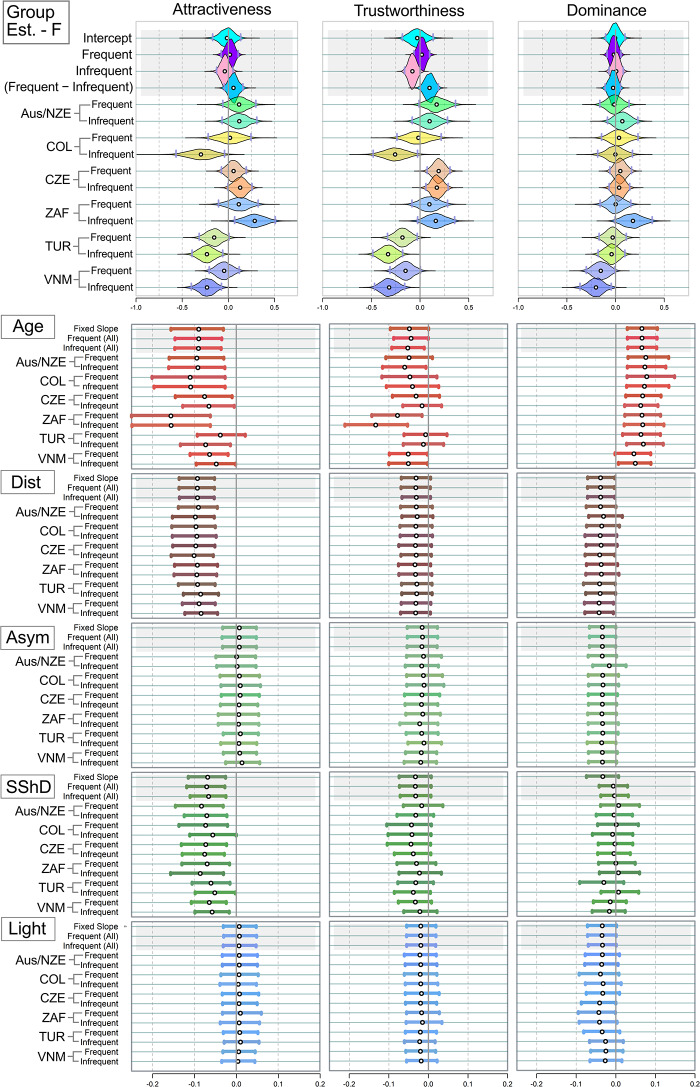
Mean estimated ratings of attractiveness, trustworthiness, and dominance in the twelve *female* stimuli samples. White point = Mean of the estimate; Grey Vertical Bars = Border of 95% percentile-based Compatibility Intervals. Dist = distinctiveness; Asym = facial asymmetry; SShD = Sexual Shape Dimorphism; Light = Skin lightness. Group Est. = Group Estimates – average ratings assigned to the faces in the group of frequent and infrequent users per population. Like in Figures 2 and 3, the country abbreviations correspond to three-letter country codes.

**Figure 5.**
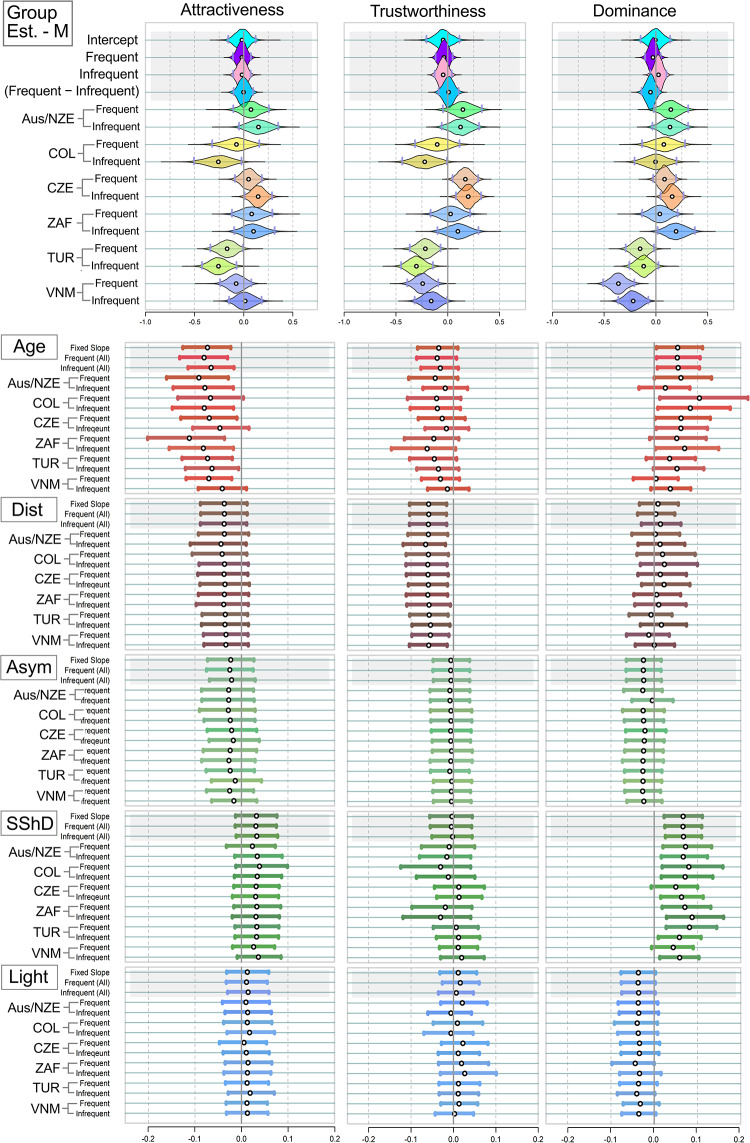
Mean estimated ratings of attractiveness, trustworthiness, and dominance in the twelve *male* stimuli samples (see the labels).

We also estimated mean ratings assigned by different samples of raters. As the upper panels of Figures 4 and 5 suggested, mean ratings obtained from different samples of raters were relatively stable. In the female stimuli sample, frequent users assigned slightly higher ratings of trustworthiness (0.10 [0.00; 0.19]. In men, no credibly non-zero tendency of a similar type was observed.

The results further showed greater variation between samples from different cultures than between samples from the same culture (Table 3). In other words, when two samples, i.e. of frequent vs infrequent users, were drawn from the same population, the differences in average assigned ratings between them were smaller than the differences between samples drawn from different populations. In the case of female dominance and male attractiveness, the 95% percentile-based Compatibility Interval spanned over zero, however, by a very small margin (Table 3).

**Table 3.** ‘Difference of differences’ – are average differences in mean estimates within a population always smaller than average differences between samples from different populations?

Female Stimuli	Attractiveness	Trustworthiness	Dominance
Mean difference within samples	0.17 [0.08; 0.27]	0.15 [0.07; 0.23]	0.10 [0.04; 0.17]
Mean difference between samples	0.24 [0.16; 0.32]	0.27 [0.21; 0.33]	0.14 [0.06; 0.21]
*Difference of the two differences*	0.08 [−0.00; 0.17]	0.12 [0.05; 0.21]	0.04 [−0.01; 0.11]
Male stimuli	Attractiveness	Trustworthiness	Dominance
Mean difference within samples	0.13 [0.06; 0.22]	0.11 [0.05; 0.19]	0.12 [0.06; 0.19]
Mean difference between samples	0.20 [0.12; 0.29]	0.25 [0.18; 0.31]	0.23 [0.17; 0.29]
*Difference of the two differences*	0.07 [−0.00; 0.17]	0.14 [0.06; 0.22]	0.11 [0.04; 0.19]

### 3.3. Effects of morphometric predictors, age, and skin lightness

*Age* negatively affected perceived attractiveness in both sexes across all samples. This effect was relatively strongest in female (stimuli) South African samples (−0.16 [−0.25; −0.06]] for both the frequent and infrequent social media users). No systematic variance based on social media use frequency was observed (Figs. 4 and 5). Age had a consistently positive effect on dominance in every *female* sample. In *males*, the effect of age on dominance was also positive in both frequent and infrequent SM users, but it was weaker than in women.

*Distinctiveness* had a negative effect on the perceived attractiveness. This effect was credibly non-zero across all female stimuli samples: raters from all samples considered more distinctive female faces as slightly less attractive (posterior mean ∼−0.09, with narrow CIs from −0.14 to −0.04). In the men sample, more distinctive facial configurations were related negatively to trustworthiness (credibly negative effect equalled −0.05 to −0.07 in every sample). For *facial asymmetry* and *skin lightness*, the effects were likely zero across all samples and for each of the three rating scales, based on the 95% CI. No systematic variance based on social media use frequency was observed.

*Sexual shape dimorphism (SShD)* influenced female attractiveness in every sample. SShD has been calculated together for male and female faces, with the female group indexed by −1 in the equation. Therefore, negative values of SShD mean more female-like facial configurations. Even though SShD was standardised before entering the analysis (zero mean, variance unity), the nature of the scale (lower values − more feminine) was preserved; more feminine facial configurations are slightly more attractive. The fact that the credibly non-zero coefficient is −0.07 for both frequent and infrequent users suggests that social media use frequency has no effect on the sex-typicality appreciation by the raters.

In contrast, while there was no systematic effect of SShD on assigned male attractiveness, there was a slight, but mostly credibly non-zero, positive effect of SShD on assigned dominance in male faces: more masculine facial configurations were perceived as slightly more dominant − an effect that was again unaffected by social media use intensity.

### 3.4. Alternative subset

For an alternative analysis, we excluded raters who scored within the 25th percentile around the median for at least two of the three social media use frequency measures. Nevertheless, this analysis also suggested that raters’ perceptions did not differ based on the frequency of social media use. Interested readers can find the analyses in the online document ‘Supplementary_Materials.pdf’ (Part III).

Dividing the raters based on a score calculated from the frequency of using three social media platforms that specialise in visual content (YouTube, TikTok, and Instagram), we mostly replicated the results of the main analysis (see Part VI of the online Supplementary Material). Notably, the trend for frequent users to agree more on female dominance became credibly non-zero (β = 0.14 [95% CI: 0.01; 0.27]).

We observed no systematic differences in ratings assigned by groups divided by travel abroad frequency and socioeconomic status (SES). The sole exception is a substantially lowered agreement between groups with the lowest SES (Table S13). While the effects seem pronounced, they are most likely due to splitting participants into two highly unequal groups for this analysis.

## 4. Discussion

Participants from geographically distant countries perceive the facial attractiveness and trustworthiness of the same set of high-quality, unedited facial images in a similar way. The range of observed correlations agrees with contemporary literature on unconstrained facial perception (Jones et al., 2021; Todorov & Oh, 2021), replicating their result using predefined rating scales.

More importantly, we identified no systematic effects of social media use, specific usage of visually oriented social media, or the two control scales (travel abroad frequency and SES during childhood) on attractiveness and trustworthiness perception. On the contrary, the cross-cultural agreement in dominance ratings is generally lower, and frequent users tend to agree on perceived dominance more than the less frequent ones.

To keep the discussion concise, we mostly refer to cognitive and adaptive explanations for cross-cultural agreement or disagreement (lack of experience with other-culture faces, Apicella et al., 2007; Kleisner et al., 2017; different cue utilisation that is explicable as an adaptation to a different environment, Marcinkowska et al., 2014, 2019) rather than shared or differing norms and values. Furthermore, the results of our study should not be directly compared to studies that use artificially created stimuli (e.g., Sofer et al., 2017) or rater-culture-matched stimuli downloaded from the internet (e.g., Sutherland et al., 2018).

Although social media use intensity, a potential source of a similar visual diet, could mediate an increase in cross-cultural agreement, our study found no strong evidence of this effect. The hypothesis that more intensive self-reported social media use would result in increased agreement was not supported: *correlations* in per-face ratings were consistently higher within the same population than between populations (i.e., between participants who were assigned to the groups of frequent and infrequent social media users). The sole exception is perceived male dominance, for which the agreement was consistently higher for frequent users.

While the results provide no support for the abovementioned hypothesis, this may be due to shared characteristics between our samples *en masse*: we estimated the concordance in ascribed characteristics between different groups at the per-face level, and in this setting, we found very good general agreement in facial first impressions across all the large-scale populations that we included. It can be reasonably assumed that all our participants were exposed to European facial variance from diverse sources (films, TV broadcasting, and the internet). For example, someone who avoids social media (including YouTube) could nevertheless consume extensive amounts of international television via streaming services. Moreover, the self-reported social media use intensity may not capture the actual time spent online and, therefore, may bias our conclusions concerning the visual diet of the participants (cf. Verbeij et al., 2021). Nevertheless, the bias is not systematic (e.g., participants do not always under – or overestimate) and it affects different groups (such as men and women, adults and adolescents, and computer and smartphone users) differently (Scharkow, 2016), for this reason, only tracking the participants’ client log files would yield more reliable data on their time spent online.

The *average ratings* assigned by different samples showed slight variation, but the differences were unrelated to the frequency or intensity of social media use. Furthermore, despite the above-mentioned cross-cultural agreement, the differences in average ratings were consistently greater between populations than within populations. Social media users also did not assign lower average ratings, as might have been expected if daily exposure to highly attractive stimuli led to harsher judgments in our study.

The effects of distinctiveness, sexual shape dimorphism, and age on perceived characteristics were largely consistent across cultures and aligned with patterns previously observed in the literature.

### 4.1. Cross-sample and cross-cultural differences

While we may reasonably expect that people in Vietnam, South Africa, and Colombia are less exposed to Central European faces, raters from these groups were able to trace the cues of *attractiveness and trustworthiness* similarly to the other groups (Figs. 2 and 3). Even though the agreement is relatively stronger between the Czech samples and Australia/New Zealand samples and it declines for the remaining samples, the decline (Czechia > WEIRD > non-WEIRD countries) is much less steep than the one reported in Kočnar et al. (2019; see Figure 3 in the referenced article). Therefore, the idea that agreement in first impressions on these scales simply decreases with increasing cultural distance is not supported by our data. Furthermore, the Australian-New Zealand sample was among the most mixed, with nearly an equal number of participants reporting European and South-East Asian ethnic origin. It suggests that greater agreement between Czechs and Australians/New Zealanders is not due to common (i.e., European) ethnic ancestry.

The level of agreement between the Czech and Turkish raters is comparable to that between Czech and Vietnamese raters, even though Turkey – a geographically closer country to Czechia, visited annually by up to 400,000 Czech citizens (Republic of Türkiye, Ministry of Cultural Heritage and Tourism, 2025) – would be expected to show greater similarity in facial first impression formation. Potentially, explaining cultural differences and similarities based on ‘ecological’ criteria (adaptation to local circumstances, level of exposure to foreign faces, and the closeness and similarity of inhabited environments) is an oversimplification. Culture, a shared set of basic values and customs (Hofstede, 2011; Schwartz, 2012), must not be treated as entirely adaptive characteristic. Values that may affect facial stereotyping may reflect characteristics better explicable as functional on the group level as compared to the individual level (Schwartz & Bilsky, 1990). Our initial stance was that the observed phenomena are explicable using evolutionary (adaptive/evolved) mechanisms (see above). Individuals are affected by the shared environment and react adaptively, which, in turn, changes what we see on the whole-sample level. Borrowing explanations from cultural anthropology and social psychology is outside the scope of this study.

South African, Australian, and New Zealand raters were recruited via Prolific, a researcher-oriented crowdsourcing platform with pre-screened, paid participants. Platform-based recruitment can introduce shared participant characteristics across countries (e.g., higher digital literacy and motivation for paid online tasks). Nevertheless, no platform-induced convergence was observed as the agreement was not higher among the Prolific-recruited groups than among those groups and other national samples (Table S1).

Our models utilised individual ratings to estimate the varying effects on both the participant (rater) and the stimulus level. This approach enhanced our confidence that the parameters we estimated were not artefacts, for example, due to per-face averaging within samples. Therefore, contrary to recent data-driven studies that challenge the concept of universally and predictably ascribed facial attractiveness (Ibáñez-Berganza et al., 2019; Sano & Kawabata, 2023), we claim that psychological constructs like trustworthiness, attractiveness, and, to a lesser extent, dominance serve as relevant verbal anchors in first impression formation, given the substantial cross-cultural agreement in their perception.

Interpersonal differences and variance among different populations likely represent different facets of the first impression formation phenomenon. Each face likely possesses some ‘parameter attractiveness’ (likewise, dominance, trustworthiness), from which individual ratings are sampled, and which is similar for raters from different *populations*. However, the way it is drawn/sampled for *individual perceivers* is subject to larger variance. While a person’s visual diet may affect this perception, its effect need not outdo the effect of other factors. In this context, social media usage is just one of many potential sources of interpersonal variance. Other factors include individual health (De Barra et al., 2013), well-being (Little et al., 2007 cf. Garza et al., 2021), personality (Štěrbová et al., 2019), and fear of domestic vs. public violence (Borras-Guevara et al., 2017). Additionally, some populations are more homogeneous (both ethnically and regarding resource distributions), while others exhibit greater diversity (World Bank Group, 2025a; see also Table S2D in Supplementary Materials). Therefore, interpersonal variance and cross-cultural differences are often mutually intermingled and thus not independent of one another.

While our participants were asked about their SES during childhood and frequency of travel abroad, the effects of these variables on cross-cultural agreement were either null or unsystematic (e.g., effects of SES on the agreement depends on the splits Tables S12–S13). As other studies, we faced the trade-off between depth and breadth of data collection.

In that regard, a methodology to bridge the gap between different types of studies is yet to be developed: On the one hand, there are experimental studies (that manipulate the environmental, visual, or social information, to which the participants are exposed (e.g., Saribay et al., 2021), together with studies that try to associate concrete aspects of an individual’s life history (De Barra et al., 2013; Borras-Guevara et al., 2019). These studies typically utilise smaller, more thoroughly examined samples and reimburse their participants. On the other hand, there are studies that use fewer metrics as predictors, or are run on many, usually online, samples (e.g., DeBruine et al., 2011; Fiala et al., 2021).

The results in our study differ from those we would expect from a laboratory-based procedure, where participants are exposed to specific visual stimuli intended to prime them (following Little et al., 2011; Saribay et al., 2021). One would expect weaker and less systematic effects outside the laboratory and without experimental manipulation. Our data align with such expectations, and we interpret the findings as evidence that the frequency of social media use does not even out the cross-cultural differences by a margin large enough to be detected in our online rating study.

Morphing or otherwise altering stimuli may introduce spurious associations that are either artefacts of the manipulation procedure or arise from a failure to control for confounding variables (DeBruine et al., 2010). This may partly explain the discrepancy between our results and those of some previous cross-cultural studies (e.g., Jones et al., 2021).

Even though people without any internet access constitute a minor part of the global population, we may be talking about quite a large minority (up to 2.6 billion people, International Telecommunication Union, 2025a, 2025b). We must therefore distinguish between two types of ‘people staying offline’. They can be as such due to their own decision, or due to unfavourable environmental conditions. In a predominantly online society, the first impressions of those who stay offline may differ for reasons other than visual diet; the values that lead these individuals to avoid the internet could also suggest that their facial preferences differ from those of the wider society. On the contrary, societies with little or no internet access usually inhabit rural, remote areas, which should alter local preferences (Marcinkowska et al., 2014, 2019), and the internet need not play a principal role in such an alteration (Batres & Perrett, 2014). Distinguishing these differences as adaptive vs. culturally induced is beyond the scope of this study.

### 4.2. Effects of anthropometric predictors: A case of strong agreement

The effects of morphometric predictors on both Attractiveness and Dominance are congruent with previous studies (Kleisner et al., 2024; Nakamura et al., 2020).

#### Distinctiveness

Female faces with less average (i.e., more distinctive) features were consistently less preferred. There was a similar tendency in male faces, in which trustworthiness, a correlate of attractiveness, was also credibly negatively related to distinctiveness. This result corroborates the previous evidence from Kleisner et al. (2024). It also importantly nuances the contribution of Apicella et al. (2007), based on which we expected that prior familiarity with faces in each population, and thus knowing which faces are average and which are not in that population, would be required for averageness to affect the ratings. Apicella et al. (2007) showed that among the Hadza, isolated hunter-gatherers, knowledge of facial variance in European faces was limited so that they, unlike Europeans, were unable to use averageness as a cue for attractiveness in European faces. We show that large-scale populations considered non-WEIRD (Vietnam, South Africa, Colombia) still possess sufficient knowledge of European facial variance to trace facial features and use them during first impression formation in a way similar to locals.

Using distinctiveness as a measure of distance from the sample mean, we could not determine whether resemblance to typical facial features of the rater’s population influenced preferences (cf Kleisner et al., 2019). In two of the populations, we do not possess local faces, so the Cross-Group Typicality/Distinctiveness Metric (Kleisner et al., 2019) with which we could explore such effects could not be obtained. Moreover, most countries in our sample can be considered multi-ethnic, which raises the question of which ‘distance to the sample mean face’ would be representative for the local raters.

#### Skin lightness

Coetzee and colleagues (Coetzee et al., 2014) showed that participants from the local population (South Africa) used colour to guide first impressions, while those from abroad (Europeans) used facial shape instead (Coetzee et al., 2014). Detailing this contribution, Kleisner et al. (2017) showed that European ratings tend to correspond to local (i.e., European) stereotypes on facial features’ variance in an African population and therefore have smaller covariance with potentially adaptive preferences within an African (Cameroonian) sample. In our case, the skin lightness has no effect on any of the scales in any of the samples, including South Africa. Neither European nor non-European raters thus used skin colour cues during first impression formation in European faces.

#### Age

Despite the narrow age range in our stimuli, we found a positive effect of age on perceived dominance and a negative one on attractiveness. The effect is present in both sexes, but more pronounced in women, which is in good agreement with previous evidence (Voegeli et al., 2021). There is, however, also evidence that in East Asian populations (represented here by Vietnamese raters), age and trustworthiness can be positively related as well (Li et al., 2022). This effect was not found, as the effects of age were relatively stable across cultures in both direction and magnitude. Results on the association between age and trustworthiness, although weaker, pointed in the same direction as those on the age-attractiveness associations.

## 5. Limitations

Our study was designed to test whether self-reported social media use intensity and frequency affect agreement between participants of different cultural backgrounds. This design decision comes with limitations: The survey distribution procedure, which was obligatorily online, and used the participants’ own device, was not optimised to get a demographically diverse, culturally representative sample of participants that would allow identifying regional differences in body ideals/most common types of plastic surgery and examine their effects on congruency in facial preferences.

We consider this, together with using self-reported measures and limited representativeness of our sample, to be certain limitations. Even so, the research suggests substantial levels of cross-cultural agreement, especially when compared to older studies using similar methodology (e.g., Kočnar et al., 2019). Further nuancing of this perspective would call for controlled laboratory studies as opposed to online, data collection.

When addressing the cross-cultural disagreement in first impressions, we focused on the frameworks of evolutionary psychology and cognitive science. We used their concepts (e.g., adaptation, visual diet and perception), not concepts borrowed from social psychology or cultural anthropology (e.g., values and cultural norms). We do not possess sufficiently detailed demographic or personal data for our raters to estimate the effects of the latter.

Furthermore, we examined the cross-cultural consistency in first impression formation, and we maintain that raters in our study assigned ratings of attractiveness and trustworthiness consistently across samples from different cultures. However, we cannot state that we have characterised general human facial preferences. The latter claim would require a more diverse sample of both stimuli and participants.

## 6. Conclusions

The results of our study suggest very good general agreement in facial first impressions across large-scale populations. It applies especially to attractiveness and trustworthiness. In contrast, dominance ratings exhibit greater variability among raters from different populations, potentially reflecting differing degrees of familiarity with Central European facial diversity. Moreover, frequent users tend to agree more with the female dominance assessment, compared to the infrequent users. The cross-cultural consistency in how morphometric features such as distinctiveness, sexual shape dimorphism (female stimuli), and age affect the perceived characteristics suggests that these traits add consensually to impression formation across cultures. Our findings contribute to the ongoing discussion on cross-cultural stability in facial perception and underscore the complexity of the task of separating the effects of social media from other sources of variation in interpersonal judgements.

## Supporting information

10.1017/ehs.2026.10042.sm001 Fiala et al. supplementary material Fiala et al. supplementary material

## Supplementary material

The supplementary material for this article can be found at https://doi.org/10.1017/ehs.2026.10042.

## Research transparency and reproducibility

All the supplementary materials, data and scripts are available at https://github.com/VojtechFiala/YUFE_Rating_study_1.

## References

[ref1] Apicella, C. L., Little, A. C., & Marlowe, F. W. (2007). Facial averageness and attractiveness in an isolated population of hunter-gatherers. *Perception*, 36(12), 1813–1820. 10.1068/p5601 18283931

[ref2] Batres, C., & Perrett, D. I. (2014). The influence of the digital divide on face preferences in El Salvador: People without internet access prefer more feminine men, more masculine women, and women with higher adiposity. *PLoS One*, 9(7), e100966. 10.1371/journal.pone.0100966 25006801 PMC4089996

[ref3] Becker, A. E. (2004). Television, disordered eating, and young women in Fiji: Negotiating body image and identity during rapid social change. *Culture, Medicine, and Psychiatry*, 28(4), 533–559. 10.1007/s11013-004-1067-5 15847053

[ref4] Boer, M., Stevens, G. W. J. M., Finkenauer, C., & van den Eijnden, R. J. J. M. (2022). The complex association between social media use intensity and adolescent wellbeing: A longitudinal investigation of five factors that may affect the association. *Computers in Human Behavior*, 128. 10.1016/j.chb.2021.107084

[ref5] Boothroyd, L. G., Jucker, J. L., Thornborrow, T., Barton, R. A., Burt, D. M., Evans, E. H., Tovée, M. J., & Tovée, M. J. (2020). Television consumption drives perceptions of female body attractiveness in a population undergoing technological transition. *Journal of Personality and Social Psychology*, 119(4), 839. 10.1037/pspi0000224 31854999

[ref6] Borras-Guevara, M. L., Batres, C., & Perrett, D. I. (2017). Aggressor or protector? Experiences and perceptions of violence predict preferences for masculinity. *Evolution and Human Behavior*, 38(4), 481–489. 10.1371/journal.pone.0211314

[ref7] Borras-Guevara, M. L., Batres, C., & Perrett, D. I. (2019). Fear of violence among Colombian women is associated with reduced preferences for high-BMI men. *Human Nature*, 30(3), 341–369. 10.1007/s12110-019-09350-8 31368014 PMC6698270

[ref8] Boudníková, O., & Kleisner, K. (2024). AI-generated faces show lower morphological diversity than real faces do. *Anthropological Review*, 87(1), 81–91. 10.18778/1898-6773.87.1.06

[ref9] boyd, D. M., & Ellison, N. B. (2007). Social network sites: Definition, history, and scholarship. *Journal of Computer-Mediated Communication*, 13(1), 210–230. 10.1111/j.1083-6101.2007.00393.x

[ref10] Brooks, R., Scott, I. M., Maklakov, A. A., Kasumovic, M. M., Clark, A. P., & Penton-Voak, I. S. (2011). National income inequality predicts women’s preferences for masculinized faces better than health does. *Proceedings of the Royal Society B: Biological Sciences*, 278(1707), 810–812. 10.1098/rspb.2010.0964 PMC3049041 21147809

[ref11] Coetzee, V., Greeff, J. M., Stephen, I. D., & Perrett, D. I. (2014). Cross-cultural agreement in facial attractiveness preferences: The role of ethnicity and gender. *PLoS One*, 9(7), e99629. 10.1371/journal.pone.0099629 24988325 PMC4079334

[ref12] Coetzee, V., & Perrett, D. I. (2011). African and Caucasian body ideals in South Africa and the United States. *Eating Behaviors*, 12(1), 72–74. 10.1016/j.eatbeh.2010.09.006 21184978

[ref13] Cook, R., Eggleston, A., & Over, H. (2022). The cultural learning account of first impressions. *Trends in Cognitive Sciences*, 26(8), 656–668. 10.1016/j.tics.2022.05.007 35697651

[ref14] De Barra, M., DeBruine, L. M., Jones, B. C., Mahmud, Z. H., & Curtis, V. A. (2013). Illness in childhood predicts face preferences in adulthood. *Evolution and Human Behavior*, 34(6), 384–389. 10.1016/j.evolhumbehav.2013.07.001

[ref15] DeBruine, L. M., Jones, B. C., Little, A. C., Crawford, J. R., & Welling, L. L. (2011). Further evidence for regional variation in women’s masculinity preferences. *Proceedings of the Royal Society B: Biological Sciences*, 278(1707), 813–814. 10.1098/rspb.2010.2200

[ref16] DeBruine, L. M., Jones, B. C., Smith, F. G., & Little, A. C. (2010). Are attractive men’s faces masculine or feminine? The importance of controlling confounds in face stimuli. *Journal of Experimental Psychology: Human Perception and Performance*, 36(3), 751–758. 10.1037/a0016457 20515201

[ref17] Eggerstedt, M., Rhee, J., Urban, M. J., Mangahas, A., Smith, R. M., & Revenaugh, P. C. (2020). Beauty is in the eye of the follower: Facial aesthetics in the age of social media. *American Journal of Otolaryngology*, 41(6), 102643. 10.1016/j.amjoto.2020.102643 32711235

[ref18] Eldaly, A. S., & Mashaly, S. M. (2022). The new dilemma of plastic surgery and social media: A systematic review. *European Journal of Plastic Surgery*, 45(3), 371–382. 10.1007/s00238-021-01891-5

[ref19] Fiala, V., Szala, A., Saribay, A. S., Leongómez, J. D., Wacewicz, S., Berenji, M., & Kleisner, K. (2025). Bringing evidence of systematic differences between the faces of social media influencers and the general population. EHBEA 2025 conference, 14-17 April, 2025, Newcastle upon Tyne: Northumbria University, UK, 108 pages, url: https://tvpollet.github.io/ehbea_2025_schedule/EHBEA_2025_Schedule_html (accessed on: 24 March 2026).

[ref20] Fiala, V., Třebický, V., Pazhoohi, F., Leongómez, J. D., Tureček, P., Saribay, S. A., Akoko, R. M., & Kleisner, K. (2021). Facial attractiveness and preference of sexual dimorphism: A comparison across five populations. *Evolutionary Human Sciences*, 3, e38. 10.1017/ehs.2021.33 37588529 PMC10427909

[ref21] Gabry, J., Češnovar, R., Johnson, A., & Bronder, S. (2025). *cmdstanr: R Interface to ‘CmdStan’* Stan Development Team and their assignees. R package version 0.9.0. https://mc-stan.org/cmdstanr/

[ref22] Garza, R., Pazhoohi, F., & Byrd-Craven, J. (2021). Women’s preferences for strong men under perceived harsh versus safe ecological conditions. *Evolutionary Psychology*, 19(3), 14747049211032352. 10.1177/14747049211032351 PMC10480609 34296646

[ref23] Gu, Z. (2022). Complex heatmap visualization. *iMeta*, 1(3), e43. 10.1002/imt2.43 38868715 PMC10989952

[ref24] Gu, Z., Eils, R., & Schlesner, M. (2016). Complex heatmaps reveal patterns and correlations in multidimensional genomic data. *Bioinformatics*, 32(18), 2847–2849. 10.1093/bioinformatics/btw313 27207943

[ref25] Hofstede, G. (2011). Dimensionalizing cultures: The hofstede model in context. *Online Readings in Psychology and Culture*, 2(1), 8. 10.9707/2307-0919.1014

[ref26] Holland, G., & Tiggemann, M. (2016). A systematic review of the impact of the use of social networking sites on body image and disordered eating outcomes. *Body Image*, 17, 100–110. 10.1016/j.bodyim.2016.02.008 26995158

[ref27] Ibáñez-Berganza, M., Amico, A., & Loreto, V. (2019). Subjectivity and complexity of facial attractiveness. *Scientific Reports*, 9(1), 8364. 10.1038/s41598-019-44655-9 31182736 PMC6557895

[ref28] International Telecommunication Union. (2025a). *Global Internet use continues to rise but disparities remain, especially in low-income regions*. https://www.itu.int/en/mediacentre/Pages/PR-2024-11-27-facts-and-figures.aspx

[ref29] International Telecommunication Union. (2025b). Internet use continues to grow, but universality remains elusive, especially in low-income regions. https://www.itu.int/itu-d/reports/statistics/2024/11/10/ff24-internet-use/

[ref30] Jones, B. C., DeBruine, L. M., Flake, J. K., Liuzza, M. T., Antfolk, J., Arinze, N. C., Ndukaihe, I. L. G., Bloxsom, N. G., Lewis, S. C., Foroni, F., Willis, M. L., Cubillas, C. P., Vadillo, M. A., Turiegano, E., Gilead, M., Simchon, A., Saribay, S. A., Owsley, N. C., Jang, C., … Coles, N. A. (2021). To which world regions does the valence-dominance model of social perception apply? *Nature Human Behaviour*, 5(1), 159–169. 10.1038/s41562-020-01007-2 33398150

[ref31] Kleisner, K., Kočnar, T., Tureček, P., Stella, D., Akoko, R. M., Třebický, V., & Havlíček, J. (2017). African and European perception of African female attractiveness. *Evolution and Human Behavior*, 38(6), 744–755. 10.1016/j.evolhumbehav.2017.07.002

[ref32] Kleisner, K., Pokorný, Š., & Saribay, S. A. (2019). Toward a new approach to cross-cultural distinctiveness and typicality of human faces: The cross-group typicality/distinctiveness metric. *Frontiers in Psychology*, 10, 124. 10.3389/fpsyg.2019.00124 30766504 PMC6365443

[ref33] Kleisner, K., Tureček, P., Roberts, S. C., Havlíček, J., Valentova, J. V., Akoko, R. M., Leongómez, J. D., Apostol, S., Varella, M. A. C., & Saribay, S. A. (2021). How and why patterns of sexual dimorphism in human faces vary across the world. *Scientific Reports*, 11(1), 5978. 10.1038/s41598-021-85402-3 33727579 PMC7966798

[ref34] Kleisner, K., Tureček, P., Saribay, S. A., Pavlovič, O., Leongómez, J. D., Roberts, S. C., Varella, M. A., Valentova, J. V., Apostol, S., Akoko, R. M., & Varella, M. A. C. (2024). Distinctiveness and femininity, rather than symmetry and masculinity, affect facial attractiveness across the world. *Evolution and Human Behavior*, 45(1), 82–90. 10.1016/j.evolhumbehav.2023.10.001

[ref35] Kočnar, T., Saribay, S. A., & Kleisner, K. (2019). Perceived attractiveness of Czech faces across 10 cultures: Associations with sexual shape dimorphism, averageness, fluctuating asymmetry, and eye color. *PLoS ONE*, 14(11), e0225549. 10.1371/journal.pone.0225549 31751432 PMC6872208

[ref36] Kratochvíl, L., & Havlíček, J. (2024). The fallacy of global comparisons based on per capita measures. *Royal Society Open Science*, 11(3), 230832. 10.1098/rsos.230832 38511080 PMC10951725

[ref37] Lakens, D. (2024). When and how to deviate from a preregistration. *Collabra: Psychology*, 10(1), 117094. 10.1525/collabra.117094

[ref38] Langlois, J. H., Kalakanis, L., Rubenstein, A. J., Larson, A., Hallam, M., & Smoot, M. (2000). Maxims or myths of beauty? A meta-analytic and theoretical review. *Psychological Bulletin*, 126(3), 390–423. 10.1037/0033-2909.126.3.390 10825783

[ref39] Li, Y., Chen, Z., Liu, X., & Qi, Y. (2022). Perceiving the facial trustworthiness: Facial age, emotional expression, and attractiveness. *Quarterly Journal of Experimental Psychology*, 75(5), 818–829. 10.1177/17470218211047176 34477460

[ref40] Little, A. C., Cohen, D. L., Jones, B. C., & Belsky, J. (2007). Human preferences for facial masculinity change with relationship type and environmental harshness. *Behavioral Ecology and Sociobiology*, 61(6), 967–973. 10.1007/s00265-006-0325-7

[ref41] Little, A. C., Jones, B. C., DeBruine, L. M., & Caldwell, C. A. (2011). Social learning and human mate preferences: A potential mechanism for generating and maintaining between-population diversity in attraction. *Philosophical Transactions of the Royal Society B: Biological Sciences*, 366(1563), 366–375. 10.1098/rstb.2010.0192 PMC3013470 21199841

[ref42] Ma, F., Xu, F., & Luo, X. (2015). Children’s and adults’ judgments of facial trustworthiness: The relationship to facial attractiveness. *Perceptual and Motor Skills*, 121(1), 179–198. 10.2466/27.22.PMS.121c10x1 26108060

[ref43] Marcinkowska, U. M., Kozlov, M. V., Cai, H., Contreras-Garduño, J., Dixson, B. J., Oana, G. A., Kaminski, G., Li, N. P., Lyons, M. T., Onyishi, I. E., Prasai, K., Pazhoohi, F., Prokop, P., Rosales Cardozo, S. L., Sydney, N., Yong, J. C., & Rantala, M. J. (2014). Cross-cultural variation in men’s preference for sexual dimorphism in women’s faces. *Biology Letters*, 10(4), 20130850. 10.1098/rsbl.2013.0850 24789138 PMC4013689

[ref44] Marcinkowska, U. M., Rantala, M. J., Lee, A. J., Kozlov, M. V., Aavik, T., Cai, H., & Dixson, B. J. (2019). Women’s preferences for men’s facial masculinity are strongest under favorable ecological conditions. *Scientific Reports*, 9(1), 3387. 10.1038/s41598-019-39350-8 30833635 PMC6399235

[ref45] McElreath, R. (2020). *Rethinking: statistical rethinking book package. R package version 2.13*. Richard McElreath. https://github.com/rmcelreath/rethinking

[ref46] Mendoza, B. A. (2022). *Face filters and their effects on users* [Diploma Thesis]. San Diego State University. https://digitalcollections.sdsu.edu/do/29179ee6-bcd0-4fca-8ac1-abe8a27b90d8

[ref47] Nakamura, K., Ohta, A., Uesaki, S., Maeda, M., & Kawabata, H. (2020). Geometric morphometric analysis of Japanese female facial shape in relation to psychological impression space. *Heliyon*, 6(10), e05148. 10.1016/j.heliyon.2020.e05148 33072915 PMC7549058

[ref48] Ortiz-Ospina, E. (2019). The rise of social media. OurWorldinData.org. https://ourworldindata.org/rise-of-social-media (Accessed 24 March 2026).

[ref49] Pavlovič, O., Fiala, V., & Kleisner, K. (2021). Environmental convergence in facial preferences: A cross-group comparison of Asian Vietnamese, Czech Vietnamese, and Czechs. *Scientific Reports*, 11, 550. 10.1038/s41598-020-79623-1 33436663 PMC7804147

[ref50] Pereira, K. J., David, V. F., Varella, M. A. C., & Valentova, J. V. (2020). Environmental threat influences preferences for sexual dimorphism in male and female faces but not voices or dances. *Evolution and Human Behavior*, 41(4), 303–311. 10.1016/j.evolhumbehav.2020.05.003

[ref51] Pokorný, Š., Pavlovič, O., & Kleisner, K. (2024). Sexual Dimorphism: The Interrelation of Shape and Color. Arch Sex Behav, 53(8), 3255–3265. 10.1007/s10508-024-02918-1 38944665 PMC11335828

[ref52] Ramphul, K., & Mejias, S. G. (2018). Is ‘Snapchat Dysmorphia’ a real issue? *Cureus*, 10(3), e2263. 10.7759/cureus.2263 29732270 PMC5933578

[ref53] Rennels, J. L., Bronstad, P. M., & Langlois, J. H. (2008). Are attractive men’s faces masculine or feminine? The importance of type of facial stimuli. *Journal of Experimental Psychology: Human Perception and Performance*, 34(4), 884–893. 10.1037/0096-1523.34.4.884 18665733

[ref54] Republic of Türkiye, Ministry of Cultural Heritage and Tourism (2025). *Yearly Bulletins*. https://www.ktb.gov.tr/EN-249299/yearly-bulletins.html

[ref55] Revelle, W. (2024). *Psych: procedures for psychological, psychometric, and personality research (Version 2.4.12)*. R package, Northwestern University. https://CRAN.R-project.org/package=psych

[ref56] Sano, T., & Kawabata, H. (2023). A computational approach to investigating facial attractiveness factors using geometric morphometric analysis and deep learning. *Scientific Reports*, 13(1), 19797. 10.1038/s41598-023-47084-x 37957245 PMC10643417

[ref57] Saribay, S. A., Pokorný, Š., Tureček, P., & Kleisner, K. (2025). Facial basis of stereotypes: Judgements of warmth and competence based on cross-group typicality/distinctiveness of faces. *British Journal of Psychology*, 116(1), 269–286. 10.1111/bjop.12738 39347699 PMC11724689

[ref58] Saribay, S. A., Tureček, P., Paluch, R., & Kleisner, K. (2021). Differential effects of resource scarcity and pathogen prevalence on heterosexual women’s facial masculinity preferences. *Evolutionary Human Sciences*, 3, e48. 10.1017/ehs.2021.42 37588556 PMC10427302

[ref59] Scharkow, M. (2016). The accuracy of self-reported internet use—A validation study using client log data. *TFO Collections*, 13–27. 10.1080/19312458.2015.1118446

[ref60] Schwartz, S. H. (2012). An overview of the Schwartz theory of basic values. *Online Readings in Psychology and Culture*, 2(1), 11. 10.9707/2307-0919.1116

[ref61] Schwartz, S. H., & Bilsky, W. (1990). Toward a theory of the universal content and structure of values: Extensions and cross-cultural replications. *Journal of Personality and Social Psychology*, 58(5), 878–891. 10.1037/0022-3514.58.5.878

[ref62] Scott, I. M., Clark, A. P., Josephson, S. C., Boyette, A. H., Cuthill, I. C., Fried, R. L., & Penton-Voak, I. S. (2014). Human preferences for sexually dimorphic faces may be evolutionarily novel. *Proceedings of the National Academy of Sciences*, 111(40), 14388–14393. 10.1073/pnas.1409643111 PMC4210032 25246593

[ref63] Scott, I. M., Swami, V., Josephson, S. C., & Penton-Voak, I. S. (2008). Context-dependent preferences for facial dimorphism in a rural Malaysian population. *Evolution and Human Behavior*, 29(4), 289–296. 10.1016/j.evolhumbehav.2008.02.004

[ref64] Singh, B., Mellinger, C., Earls, H. A., Tran, J., Bardsley, B., & Correll, J. (2022). Does cross-race contact improve cross-race face perception? A meta-analysis of the cross-race deficit and contact. *Personality and Social Psychology Bulletin*, 48(6), 865–887. 10.1177/01461672211024463 34176344

[ref65] Sofer, C., Dotsch, R., Oikawa, M., Oikawa, H., Wigboldus, D. H. J., & Todorov, A. (2017). For your local eyes only: Culture-specific face typicality influences perceptions of trustworthiness. *Perception*, 46(8), 914–928. 10.1177/0301006617691786 28152651

[ref66] Sorokowski, P., Kościński, K., & Sorokowska, A. (2013). Is beauty in the eye of the beholder but ugliness culturally universal? Facial preferences of Polish and Yali (Papua) people. *Evolutionary Psychology*, 11(4), 907–925. 10.1177/147470491301100414

[ref67] Sorokowski, P., Sorokowska, A., & Kras, D. (2013). Face color and sexual attractiveness: Preferences of Yali people of Papua. *Cross-Cultural Research*, 47(4), 415–427. 10.1177/1069397113485540

[ref68] Stan Development Team. (2020). *RStan: the R interface to Stan. R package version 2.21.2*. http://mc-stan.org/

[ref69] Štěrbová, Z., Tureček, P., & Kleisner, K. (2019). She always steps in the same river: Similarity among long-term partners in their demographic, physical, and personality characteristics. *Frontiers in Psychology*, 10, 52. 10.3389/fpsyg.2019.00052 30804826 PMC6371050

[ref70] Strom, M. A., Zebrowitz, L. A., Zhang, S., Bronstad, P. M., & Lee, H. K. (2012). Skin and bones: The contribution of skin tone and facial structure to racial prototypicality ratings. *PLoS One*, 7(7), e41193. 10.1371/journal.pone.0041193 22815966 PMC3399873

[ref71] Sun, Y. (2023). Beauty in RED: how social media influencers construct aesthetic norms of Chinese women. 10.7302/21608

[ref72] Sutherland, C. A. M., Liu, X., Zhang, L., Chu, Y., Oldmeadow, J. A., & Young, A. W. (2018). Facial first impressions across culture: Data-driven modeling of Chinese and British perceivers’ unconstrained facial impressions. *Personality and Social Psychology Bulletin*, 44(4), 521–537. 10.1177/0146167217744194 29226785

[ref73] Todorov, A., & Oh, D. (2021). The structure and perceptual basis of social judgments from faces. In B. Gawronski (Ed.), *Advances in experimental social psychology* (vol 63, pp. 189–245). Academic Press. 10.1016/bs.aesp.2020.11.004

[ref74] Van Den Eijnden, R., Koning, I., Doornwaard, S., Van Gurp, F., & Ter Bogt, T. (2018). The impact of heavy and disordered use of games and social media on adolescents’ psychological, social, and school functioning. *Journal of Behavioral Addictions*, 7(3), 697–706. 10.1556/2006.7.2018.65 30264607 PMC6426368

[ref75] Verbeij, T., Pouwels, J. L., Beyens, I., & Valkenburg, P. M. (2021). The accuracy and validity of self-reported social media use measures among adolescents. *Computers in Human Behavior Reports*, 3, 100090. 10.1016/j.chbr.2021.100090

[ref76] Violot, C., Elmas, T., Bilogrevic, I., & Humbert, M. (2024). Shorts vs. Regular videos on Youtube: A comparative analysis of user engagement and content creation trends. Proceedings of the 16th ACM Web Science Conference Stuttgart, Germany New York, NY, USA: Association for Computing Machinery. pp. 213–223. 10.1145/3614419.3644023

[ref77] Voegeli, R., Schoop, R., Prestat-Marquis, E., Rawlings, A. V., Shackelford, T. K., & Fink, B. (2021). Crosscultural perception of female facial appearance: A multi-ethnic and multi-centre study. *PLoS One*, 16(1), e0245998. 10.1371/journal.pone.0245998 33481957 PMC7822532

[ref78] World Bank Group. (2025a). *Gini Index*. https://data.worldbank.org/indicator/SI.POV.GINI

[ref79] World Bank Group. (2025b). *Individuals using the internet (% of population)*. https://data.worldbank.org/indicator/IT.NET.USER.ZS

[ref80] Zebrowitz, L. A. (2017). First impressions from faces. *Current Directions in Psychological Science*, 26(3), 237–242. 10.1177/0963721416683996. 28630532 PMC5473630

[ref81] Zhan, J., Liu, M., Garrod, O. G., Daube, C., Ince, R. A., Jack, R. E., & Schyns, P. G. (2021). Modeling individual preferences reveals that face beauty is not universally perceived across cultures. *Current Biology*, 31(10), 2243–2252. 10.1016/j.cub.2021.03.013 33798430 PMC8162177

