# Supplementary material for: Self-reported social media use does not affect cross-cultural consensus in first impressions
Source: Evol Hum Sci. 2026 Mar 24;8:e10. doi: 10.1017/ehs.2026.10042 (PMC13112124; doi:10.1017/ehs.2026.10042)
Supplement: Fiala et al. supplementary material [file S2513843X26100425sup001.pdf]

Contents

Part I. Demographics 1

Part II. Splitting participants based on social media use 5

Part III. Results: 12

Subsection 1: ATTR/TRUSTW/DOM: Model Comparison 12

Note: A sampling problem experienced during posterior evaluation: 14

Subsection 1.2. Models with attractiveness, trustworthiness, and dominance as the sole response variable: model predictions 15

1.2.1. Figure S6 - analogue of Figure 3 in the article “correlation heatmaps”......................16

1.2.2. An analogue of Fig. 3 in the article “Means & slopes: Females” 18

1.2.3. An analogue of Figure 4 in the article “Means & slopes: Males” 20

Subsection 1.3. Model with the three response variables - attractiveness, trustworthiness, and dominance (main model): supplementary information on the model transcription and predictions 21

Part IV. Model with the three response variables (attractiveness, trustworthiness, dominance), raters in the 25% band around median SMU_score excluded 23

Part V: Travel abroad & SES during childhood 29

Part 3.1. Travel abroad 29

Part 3.2. Socioeconomic status during childhood – ver. 1 34

Part 3.3. Socioeconomic status during childhood – ver. 2 39

Part VI: Score based on 3 visually oriented SM 44

References 50

Part I. Demographics

Table S1A. Basic demographic description of the sample of the participants (raters)

Culture	Number part's	Of it females/males	Age [yrs]	Height [cm]	Weight [kg]
Australia + New Zeal.	53	28 / 24	34.51 ± 12.62	169.57 ± 8.94	73.81 ± 20.77
Colombia	32	13 / 19	29.31 ± 12.54	164.6 ± 10.12*	62.60 ± 11.65
Czech Republic	152	119 / 32	35.94 ± 10.13	170.3 ± 8.23	72.24 ± 16.97
South Africa	47	32 /15	25.62 ± 4.28	165.66 ± 10.11	80.18 ± 24.86
Turkey	79	63 / 15	22.15 ± 3.03	168.79 ± 7.88	63.24 ± 14.88
Vietnam	72	42 / 28	26.36 ± 7.46	163.78 ± 9.4	60.26 ± 11.35

Table S1B. Basic demographic description of the sample of the female participants (raters)

Culture	Number part’s	-	Age	Height	Weight
Australia + New Zealand	28	-	35.29 ± 14.84	165.96 ± 7.93	70.39 ± 25.52
Colombia	19	-	28.5 ± 12.56	158.00 ± 6.01	55.85 ± 7.26
CZech Republic	119	-	35.97 ± 10.21	167.51 ± 5.91	68.41± 15.19
South Africa	32	-	26.12 ± 4.55	153.03 ± 31.74	79.38 ± 26.92
Turkey	63	-	22.33 ± 3.25	156.15 ± 27.87	59.43 ± 11.45

Vietnam	42	-	25.9 ± 7.8	155.88 ± 13.18	53.36 ± 7.39

Table S1C. Basic demographic description of the sample of the male participants (raters)

Culture	Number part's	-	Age [yrs]	Height [cm]	Weight [kg]
Australia + New Zealand	24	-	33.33 ± 9.88	174.04 ± 8.27	77.54 ± 13.41
Colombia	13	-	30.67 ± 12.95	173.64 ± 7.09	71.82 ± 10.19
Czech Republic	32	-	36.31 ± 9.8	180.56 ± 7.63	86.47 ± 16.13
South Africa	15	-	24.53 ± 3.54	174.33 - 7.29	81.87 ± 20.52
Turkey	15	-	21.27 ± 1.79	178.6 ± 6.31	75.87 ± 15.97
Vietnam	28	-	26.54 ± 6.82	173.43 ± 27.82	68.43 ± 6.71

*6 of the Colombian participants did not move the scale from the initial value (80 cm). Due to error during survey preparation, the question was not correctly marked as required.

Please note that small number of participants decided to not specify their sex or other demographic parameters. Consequently, numbers of participants in Table 1C and 1B need not add to the numbers in 1A.

Table S2A. Selected geographic descriptors of raters – selected country of location

Sample	Selected Location	Counts
Australia + New Zealand	AUS (Australia)	35
	NZE (New Zealand)	18
Colombia	COL (Colombia)	32
Czech Republic	CZE (Czech Republic)	144
	SVK (Slovakia)	7
	Other	3
South Africa	ZAF (South Africa)	47
Turkey	TUR (Turkey)	79
Vietnam	VNM (Vietnam)	66
	SGP (Singapore)	2
	Other	4

Table S2B. Selected geographic descriptors of raters – selected mother tongue

Sample	Mother tongue	Counts
Australia + New Zealand	English	43
	Vietnamese	2
	Mandarin	2
	Cantonese	2
	Other (Afan Oromo, Arabic, Bengali, Marathi)	4
Colombia	Spanish	32
Czech Republic	Czech	134
	Slovak	15
	Other (Bulgarian, Hungarian, Russian)	3
South Africa	English	21
	Zulu / IsisZulu	9
	Xhosa / IsiXhosa	4
	Setswana	3

	Tschivenda	3
	Afrikaans	2
	Tsonga	2
	Other (IsiNdebele, Sepedi, Sesotho)	3
Turkey	Türkçe (Turkish)	74
	Armenian (Ermenice)	2
	Other (English, German, Romanian)	3
Vietnam	Vietnamese (Tiếng việt)*	72

*Below, Vietnamese participants only rarely report they are of Vietnamese ethnicity. Nevertheless, all of them report "Tiếng việt" as their mother tongue. This result further highlights that our understanding of the concept of ethnicity does not fit well in the Vietnamese context.

Table S2C. Selected geographic descriptors of raters – passport nationality

Sample	Selected Location	Counts
Australia + New Zealand	Australia	26
	New Zealand	17
	Other (Bangladesh, China, Ethiopia, Ireland, Vietnam)	5
	Not reported	4
Colombia	Colombia	32
Czech Republic	CZE	135
	SVK	16
	Bulgaria	1
South Africa	South Africa	44
	Zimbabwe	2
	India	1
Turkey	Turkey	73
	Other (Armenia, Azerbaijan, Germany, Moldova, Romania)	5
	Not reported	1
Vietnam	VNM	67
	Not reported	5

Ethnicity: We asked the participants to report their ethnicity in writing (open question). Majority of the participants responded in the mother tongue. Individual responses are available in the table "OLDEMOG.Rdata" in the github folder of the survey (https://github.com/VojtechFiala/YUFE_Rating_study_1). The Table (2D) below present decoded, bulk-categorised responses. Participants were asked on "how would they define 'ethnicity'" (responses available in the folder linked above) and then requested to do the following: "Based on the reply above, please, report your self-perceived ethnicity".

They were encouraged to skip the ethnicity-related questions if they were not sure in how to respond, or to report their doubts. The table reveals that for many, it was difficult to self-define with regard to ethnicity. This is also probable reason why there are so many participants who decided to leave this question unanswered. Please mind that we are talking about participants, who fulfilled all the three attention checks, completed initial quiz, rated the faces with a variable number and answered questions related to social media. We therefore think that doubts about the concept of ethnicity (or lack of trust) are the main reason of leaving this question blank.

Table S2D. Self-reported ethnicity of the participants

Sample	Categorised Ethnicity	Countrs
Australia + New Zealand	Caucasian / European /White	21
	South-East Asian	15
	Australia / New Zealand (general / civic identity)	9
	Mixed / Multi-ethnic origin	2
	Other	4
	NA	2
Colombia	Mestizo	7
	Mixed (other than Mestizo)	2
	NA (not reported) or None	12
	Colombian (no further details)	2
	Colombian of African origin	2
	Latino	4
	Other	3
Czech Republic	White / Czech / European	93
	None	3
	Not reported	47
	Other	9
South Africa	Black / African	32
	Coloured / Mixed Race	5
	Indian / South Asian	2
	White	2
	South African (General / Civic Identity)	5
	None	1
Turkey	Turkish / Türk	44
	Balkan origin	4
	Armenian	4
	Mixed, Minor, Religion-based	4
	Turkish Citizen (General / Civil Identity)	3
	NA + not defined	20
Vietnam	Vietnamese / Kinh (majority ethnic group)	8
	Asian (regional / continental identity)	7
	“Da vàng” / Yellow race descriptors	9
	Humanity / Universalist	5
	Undefined (personal / narrative answers)	5
	NA+ not defined	38

Table S3. Device the participants used to complete the survey

Device [↓] Culture [→]	Combined	Australia + New Zeal.	Colobia	Czechia	South Africa	Turkey	Vietnam
Android-Mobile	54	1	1	37	6	3	6
Android-Tablet	2	0	0	1	0	0	1
iPad	7	3	0	0	0	3	1
iPhone	52	0	0	15	0	14	23
Linux-PC	6	1	1	1	2	1	0
Mac/OS-X	74	11	5	14	5	32	7
Windows-PC	240	37	25	84	34	26	34

The same table repeated with relative counts (divided by sample-specific sum of participants)

Device [↓] Culture [→]	Combined	Australia + New Zeal.	Colobia	Czechia	South Africa	Turkey	Vietnam

Android-Mobile	0.124	0.019	0.031	0.243	0.128	0.038	0.083
Android-Tablet	0.005	0.000	0.000	0.007	0.000	0.000	0.014
iPad	0.016	0.057	0.000	0.000	0.000	0.038	0.014
iPhone	0.120	0.000	0.000	0.099	0.000	0.177	0.319
Linux-PC	0.014	0.019	0.031	0.007	0.043	0.013	0.000
Mac/OS-X	0.170	0.208	0.156	0.092	0.106	0.405	0.097
Windows-PC	0.552	0.698	0.781	0.553	0.723	0.329	0.472

Part II. Splitting participants based on social media use

Our goal was to get a score or identify a factor that accounted for substantial part of variance in the participants' responses on social media use frequency and intensity.

We asked the following questions that were related to lifestyle, economic situation, and social media use:

SMU_i_Boer:
Q1 How often PER DAY do you look at social network sites?
Q2 How often A WEEK do you post, photo or video on social network sites?
Q3 How often A WEEK you 'like' your posts, photos or videos of others on social network sites?

All the three questions listed Facebook, X[Twitter], Instagram, TikTok or YouTube as example social media. The responses were anchored as follows:
1 = Never or less than once a day (Q1) or week (Q2, Q3)
2 = 1-2 times per day (Q1) or week (Q2, Q3)
3 = 3-5 times a day (Q1) or week (Q2, Q3)
4 = 6-10 times a day (Q1) or week (Q2, Q3)
5 = 11-20 times a day (Q1) or week (Q2, Q3)
6 = 21-40 times a day (Q1) or week (Q2, Q3)
7 = more than 40 times (Q1) or week (Q2, Q3)

Subsequently, participants were asked "How frequently do you use social media":

Q4 Facebook
Q5 YouTube
Q6 Instagram
Q7 TikTok
Q8 X [Twitter]
Q9 WeChat / Zulu (VN) / WhatsApp (Colombia)

Note: The last of the questions was excluded since it did not use the same social media in every country. Some collaborators considered WeChat not relevant for the local audience. We decided it is more informative to get data on a locally popular social media and not on a globally popular social media that is, according to the local collaborators' knowledge, unknown in their country.

The responses were anchored as follows:
1 = I do not use it,
2 = Once a week or less,
3 = Several times a day, not daily,
4 = Once a day,

5 = Several times a day,
6 = Frequently during the day
Participants were also allowed to list their specific social media. They rarely did, and if so, no single social media (one could think of Threads, Bluesky, Reddit, Mastodon, OnlyFans, etc.) prevailed enough to establish another category.

Next, participants were asked whether they use social media actively or passively. In this context, actively means "posting, commenting...", semi-actively means "only liking and commenting, not posting own content", passively = "only watching the content, not reacting to it in any way, not posting own content".

Q10: Facebook
Q11: YouTube
Q12: Instagram
Q13: TikTok
Q14: X [Twitter]
Q15: WeChat / Zulu (VNM) / WhatsApp (COL).

Additionally, there were three open questions in which participants could add other social media. Again, it was rarely the case they did.
All the questions were anchored as
1 = I don’t use it;
2 = Passively
3 = Semi-Actively
4 = Actively

Subsequently, we asked the participants questions intended to check for potentially problematic aspects of social media use:
Q16: “I spend a lot of time on online social media”
Q17: “I'm losing interest in other hobbies because of online social media”
Q18: “I prefer following people and events on social media to interactions outside of it”
Q19: “If for some reason I can't visit social networks, I feel like I'm missing out”
All anchored as follows:
1 = totally disagree
2 = mostly disagree
3 = undecided
4 = mostly agree
5 = totally agree

Small subset of the participants did not see these questions as they marked “I do not use social media”. These participants were assigned lowest possible score in every question (correspond “I do not use this social media”, “I do not post” etc),

Q20: “I travel abroad:”
1 = Never
2 = Very rarely
3 = Rarely
4 = Occasionally
5 = Rather often
6 = Often

Q21: “In childhood, our family had:”
1 = Poor (“constant financial difficulties”)
2 = Lower CL (“rather little money, compared to my peers I had to cut back”)
3 = Middle (“sometimes more, sometimes less, but we managed to get by without losing our level”)
4 = Upper CL (“enough money, although sometimes we had to cut back”)
5 = Rich (“rich material security, money was never an issue”)
“Prefer not to disclose” coded as “NA”

To explore the underlying factors, we subsetted two series of questions [pseudo-code]:

```
smu_names_12 <- c(Q1:Q8, Q16:Q19)
smu_names_8 <- c(Q1:Q8)
```

Following the subset, we checked for the number of underlying factors (the commands below are functions of package psych in R):

```
fa.parallel(rho, n.obs = nrow(smu_names_12), fm="ml", fa="both")
```

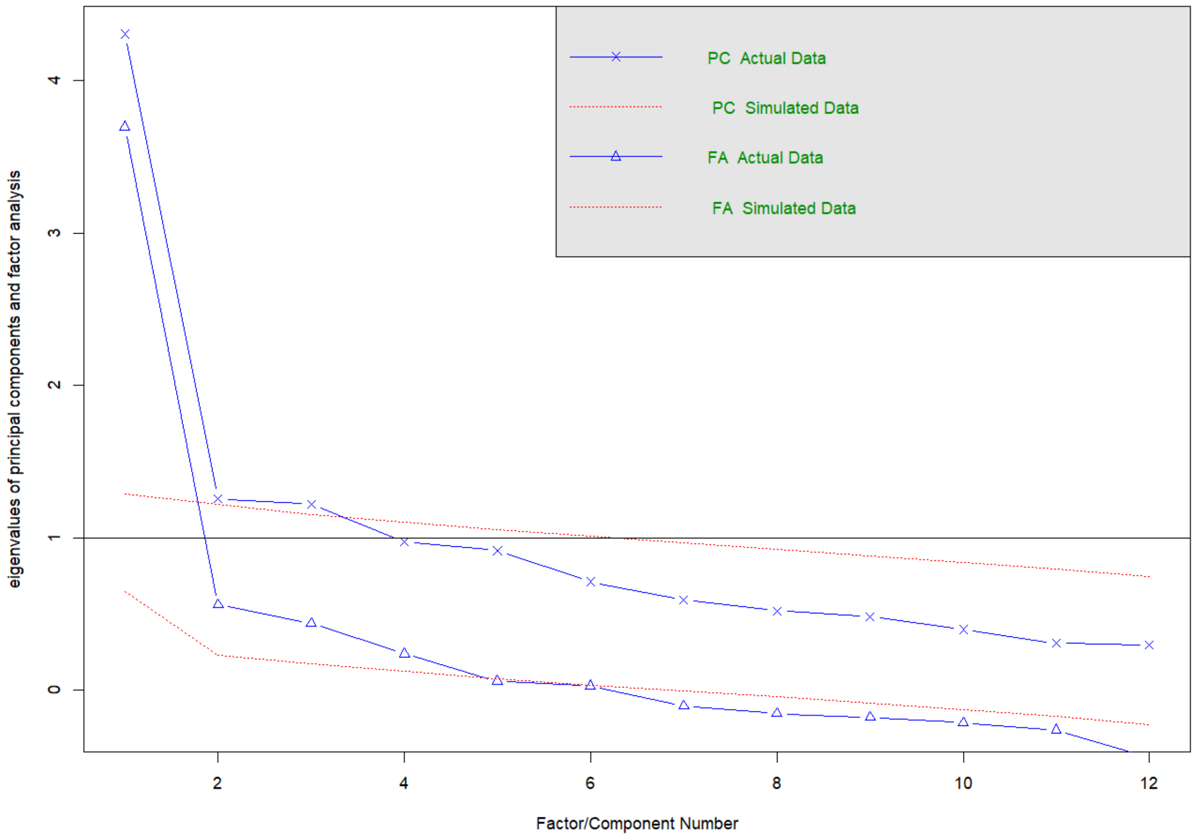

Figure S1. Number of underlying factors proposed by factor analysis, based on twelve questions (Q1-Q8, Q16-Q19).

Analogically for the subset of questions without the “Negative Use” (i.e., without Q16-Q19):

```
fa.parallel(rho, n.obs = nrow(smu_names_8), fm="ml", fa="both")
```

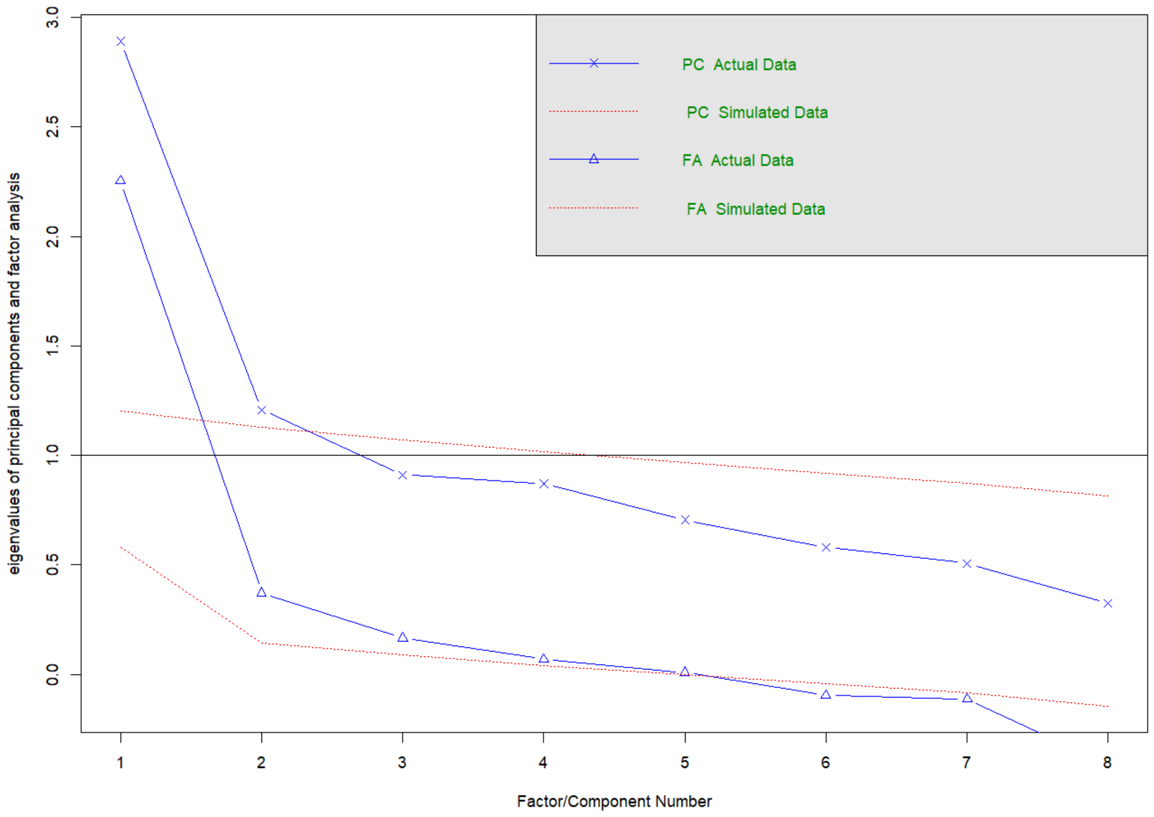

Figure S2. Number of underlying factors proposed by factor analysis, based on twelve questions (Q1-Q8).

Nevertheless, elbow method suggests there is either one or two credible underlying factors. We explored all the potentially credible solutions [pseudo-code]:

```
efa_12_ml <- fa(r = rho12, nfactors = 1 OR 2 OR 3 OR 4, fm = "ml", rotate = "oblimin")
efa_8_ml <- fa(r = rho12, nfactors = 1 OR 2 OR 3 OR 4, fm = "ml", rotate = "oblimin")
```

Table S4A and S4B present the results of an exploratory factor analysis, showing the factor loadings of the observed variables on three latent factors and the proportion of variance explained by each factor.

**Table S4A**. Factor loading, different factor solutions, layout with EIGHT questions						
Loadings (ONE factor):		Loadings (TWO factors):		Loadings (THREE factors):		
	ML1	ML1	ML2	ML1	ML2	ML3
SMU_i_Boer_1	0.636		0.993		0.992	
SMU_i_Boer_2	0.432	0.446				0.996
SMU_i_Boer_3	0.604	0.381	0.283	0.266	0.317	0.19
Freq_FCB		-0.178	0.323	-0.337	0.333	0.255
Freq_YT	0.334	0.237	0.123	0.208	0.147	
Freq_INS	0.724	0.587	0.2	0.568	0.232	
Freq_TT	0.626	0.746		0.73		
Freq_XTW	0.509	0.592		0.519		0.12
	ML1	ML2	ML2	ML3	ML1	ML2
SS loadings	2.25	1.683	1.232	1.353	1.271	1.11
Proportion Var	0.281	0.21	0.154	0.169	0.159	0.139
Cumulative Var	NA	0.21	0.364	0.169	0.328	0.467

**Table S4B**. Factor loading, different factor solutions, layout with TWELVE questions						
Loadings (ONE factor):		Loadings (TWO factors):		Loadings (THREE factors):		
	ML1	ML1	ML2	ML1	ML2	ML3
SMU_i_Boer_1	0.668	0.737			0.728	0.113
SMU_i_Boer_2	0.388	0.403			0.167	0.287
SMU_i_Boer_3	0.601	0.595			0.399	0.242
negative_1	0.801	0.557	0.311	0.352	0.595	
negative_2	0.649		0.787	0.801		
negative_3	0.525		0.664	0.641		
negative_4	0.625		0.724	0.711		
Freq_FCB	0.14	0.177			0.479	-0.295
Freq_YT	0.281	0.308			0.132	0.225
Freq_INS	0.649	0.766			0.358	0.508
Freq_TT	0.546	0.543				0.638
Freq_XTW	0.421	0.419				0.633
	ML1	ML1	ML2	ML2	ML1	ML3
SS loadings	3.683	2.556	1.695	1.702	1.458	1.359
Proportion Var	0.307	0.213	0.141	0.142	0.122	0.113
Cumulative Var	NA	0.213	0.354	0.142	0.263	0.377

Correlations between the underlying scores, obtained using different types of analyses. Below, we briefly define how different scores were obtained. For a complete analysis, refer to the script:

**Table S5**. This is how different scores were obtained.	
fa_8_ml	The sole underlying factor; we are using the questions from “SMU-i-Boer” & the intensity of use of specific social media, calculated with maximum likelihood factor extraction method (ML); questions Q1-8.
fa_8_uls	The sole underlying factor, we’re using the questions from “SMU-i-Boer” & the intensity of use of specific soc. med., calculated with Unweighted Least Squares extract. meth. (ULS/WLSMV).
CFA_smu_score_8_no_covariance	An analogue of„*fa_8_lm*“, bult obtained using Confirmatory Factor Analysis (R package lavaan; Roesseel, 2012). “no_covariance” means that in the model, we did not consider covariance between SMU-i 1 a SMU-i 3, which would be beneficial, as covariance table yielded from the model suggested.
CFA_smu_score_8_covariance	Same as above, but the CFA included covariance between SMU-i 1 and 3.
fa_12_ml_1FA	Underlying factor (ML1), if we take all the 12 questions (including “Negative Use”), “force” the analysis to use just one factor solution & maximum likelihood extract.
fa_12_ml_2FA	Underlying factor (ML2), if we take all the 12 questions (including “Negative Use”), we force the analysis to two factors solution & maximum likelihood extract. This factor should correspond to the “Negative Use Score.“
fa_12_ml_2FA_aka_NEG	Underlying factor of the negative questions (Negative Use Score). Should be almost identical to the factor above.
fa_12_uls_1FA	Underlying factor (ML1), if we take all the 12 questions (including “Negative Use”), we force the analysis to use just one factor solution & ULS extract.
fa_12_uls_2FA	Same as “fa_12_ml_2FA” (ML2), with ULS Extract
fa_12_ml_3FA_first	First factor, if three factor solution, ML1
fa_12_ml_3FA_aka_NEG	Second factor, if three factor solution ("negative use score"), ML2
fa_12_ml_3FA_third	Third factor, if three factor solution, ML3
fa_12_uls_3FA_first	First factor, if three factor solution, ULS1
fa_12_uls_3FA_aka_NEG	Second factor, if three factor solution ("negative use score"), ULS3
fa_12_uls_3FA_third	Third factor, if three factor solution, ULS3

CFA_smu_score_12_SMU_negsep	CFA-based score, the underlying factor is fitted as dependent on the questions that correspond to SMU_score above, but the “Negative Use Score” is in the same CFA
CFA_smu_score_12_NEG	...this is the “Negative Use Score” from the same equation
CFA_smu_score_12_SMU_negNEsep	CFA-based score, when we force the equation to consider all the twelve questions

Table S6. Correlations between the above-defined underlying factors (bolded is the row /column showing the correlations between factors we decided to use to split the participants [1] and factors we decided not to use). If the table came out disordered on your display, try to change the font size (happened to us in Google Doc).

	1	2	3	4	5	6	7	8	9
**fa_8_ml [1]**		**0.999**	**0.991**	**0.986**	**0.891**	**0.951**	**0.606**	**0.897**	**0.969**
fa_8_uls [2]	**0.999**		0.990	0.986	0.888	0.946	0.604	0.895	0.967
CFA_smu_score_8_no_covariance [3]	**0.991**	0.990		0.996	0.894	0.950	0.616	0.899	0.967
CFA_smu_score_8_covariance [4]	**0.986**	0.986	0.996		0.883	0.937	0.606	0.890	0.960
fa_12_ml_1FA [5]	**0.891**	0.888	0.894	0.883		0.977	0.877	0.999	0.972
fa_12_ml_2FA [6]	**0.951**	0.946	0.950	0.937	0.977		0.759	0.974	0.994
fa_12_ml_2FA_aka_NEG [7]	**0.606**	0.604	0.616	0.606	0.877	0.759		0.879	0.751
fa_12_uls_1FA [8]	**0.897**	0.895	0.899	0.890	0.999	0.974	0.879		0.974
fa_12_uls_2FA [9]	**0.969**	0.967	0.967	0.960	0.972	0.994	0.751	0.974	
fa_12_ml_3FA_first [10]	**0.867**	0.856	0.870	0.845	0.932	0.966	0.708	0.918	0.935
fa_12_ml_3FA_aka_NEG [11]	**0.572**	0.571	0.581	0.575	0.848	0.721	0.993	0.851	0.717
fa_12_ml_3FA_third [12]	**0.891**	0.903	0.882	0.903	0.768	0.789	0.556	0.790	0.847
fa_12_uls_3FA_first [13]	**0.861**	0.851	0.864	0.838	0.923	0.958	0.698	0.910	0.927
fa_12_uls_3FA_aka_NEG [14]	**0.566**	0.566	0.575	0.570	0.840	0.712	0.992	0.844	0.709
fa_12_uls_3FA_third [15]	**0.892**	0.904	0.883	0.903	0.773	0.793	0.564	0.794	0.850
CFA_smu_score_12_SMU_negsep [16]	**0.961**	0.958	0.973	0.964	0.967	0.988	0.757	0.968	0.991
CFA_smu_score_12_NEG [17]	**0.735**	0.731	0.752	0.741	0.957	0.890	0.935	0.952	0.873
CFA_smu_score_12_SMU_negNEsep [18]	**0.870**	0.867	0.885	0.877	0.992	0.962	0.885	0.991	0.958

	10	11	12	13	14	15	16	17	18
**fa_8_ml [1]**	**0.867**	**0.572**	**0.891**	**0.861**	**0.566**	**0.892**	**0.961**	**0.735**	**0.870**
fa_8_uls [2]	0.856	0.571	0.903	0.851	0.566	0.904	0.958	0.731	0.867
CFA_smu_score_8_no_covariance [3]	0.870	0.581	0.882	0.864	0.575	0.883	0.973	0.752	0.885
CFA_smu_score_8_covariance [4]	0.845	0.575	0.903	0.838	0.570	0.903	0.964	0.741	0.877
fa_12_ml_1FA [5]	0.932	0.848	0.768	0.923	0.840	0.773	0.967	0.957	0.992
fa_12_ml_2FA [6]	0.966	0.721	0.789	0.958	0.712	0.793	0.988	0.890	0.962
fa_12_ml_2FA_aka_NEG [7]	0.708	0.993	0.556	0.698	0.992	0.564	0.757	0.935	0.885
fa_12_uls_1FA [8]	0.918	0.851	0.790	0.910	0.844	0.794	0.968	0.952	0.991
fa_12_uls_2FA [9]	0.935	0.717	0.847	0.927	0.709	0.850	0.991	0.873	0.958
fa_12_ml_3FA_first [10]		0.655	0.611	0.997	0.643	0.617	0.931	0.870	0.916
fa_12_ml_3FA_aka_NEG [11]	0.655		0.559	0.637	0.999	0.569	0.723	0.913	0.857
fa_12_ml_3FA_third [12]	0.611	0.559		0.597	0.558	0.999	0.831	0.626	0.758
fa_12_uls_3FA_first [13]	0.997	0.637	0.597		0.626	0.600	0.924	0.859	0.907
fa_12_uls_3FA_aka_NEG [14]	0.643	0.999	0.558	0.626		0.567	0.715	0.904	0.850
fa_12_uls_3FA_third [15]	0.617	0.569	0.999	0.600	0.567		0.835	0.632	0.763
CFA_smu_score_12_SMU_negsep [16]	0.931	0.723	0.831	0.924	0.715	0.835		0.880	0.966
CFA_smu_score_12_NEG [17]	0.870	0.913	0.626	0.859	0.904	0.632	0.880		0.971
CFA_smu_score_12_SMU_negNEsep [18]	0.916	0.857	0.758	0.907	0.850	0.763	0.966	0.971	

The figure below shows how participants are distributed along the SMU_score. The taller the column, the more participants at a given score.

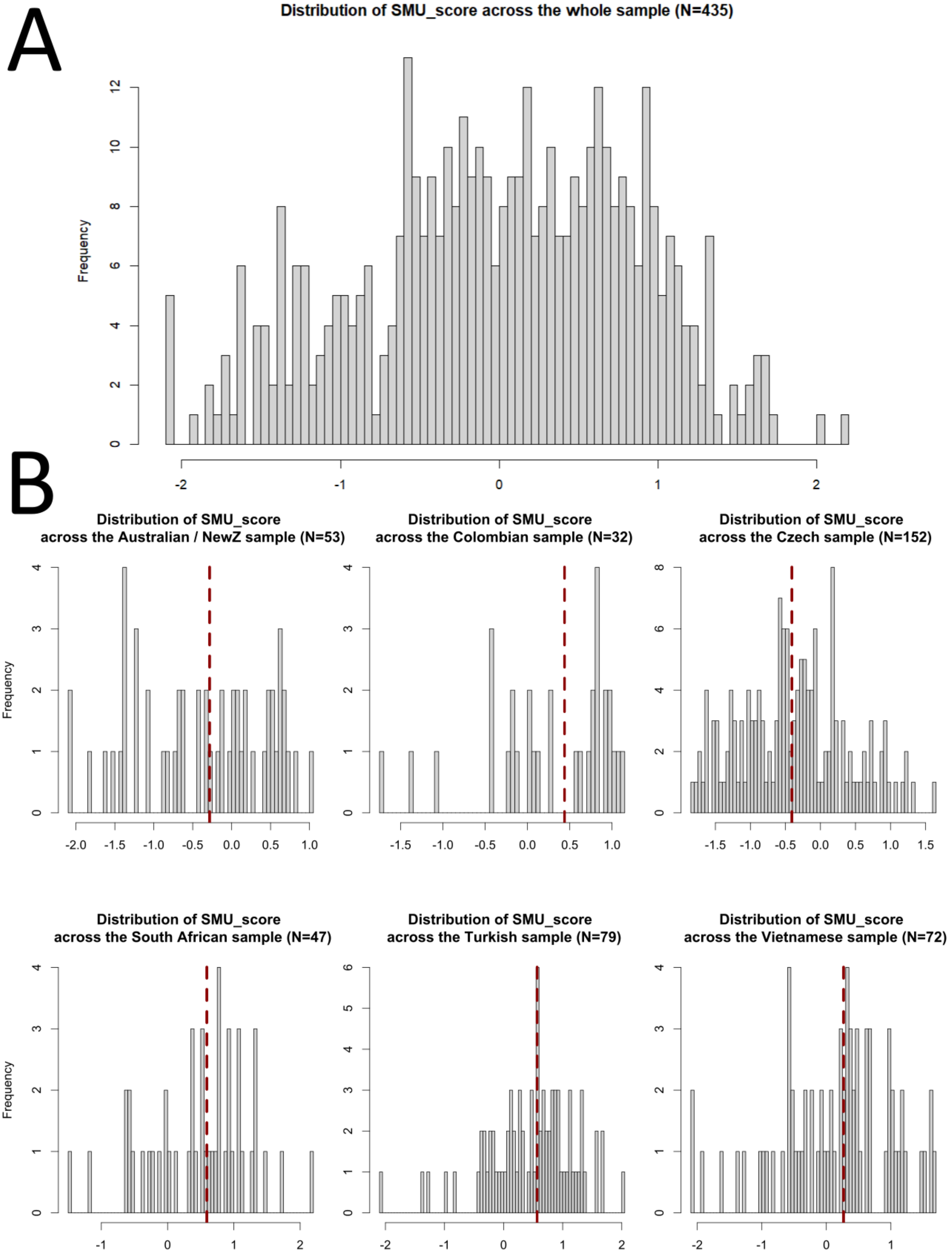

Fig S3. Distribution of SMU_score in the pooled sample and in six regionally based samples. Red vertical line stands for median in the specific sample.

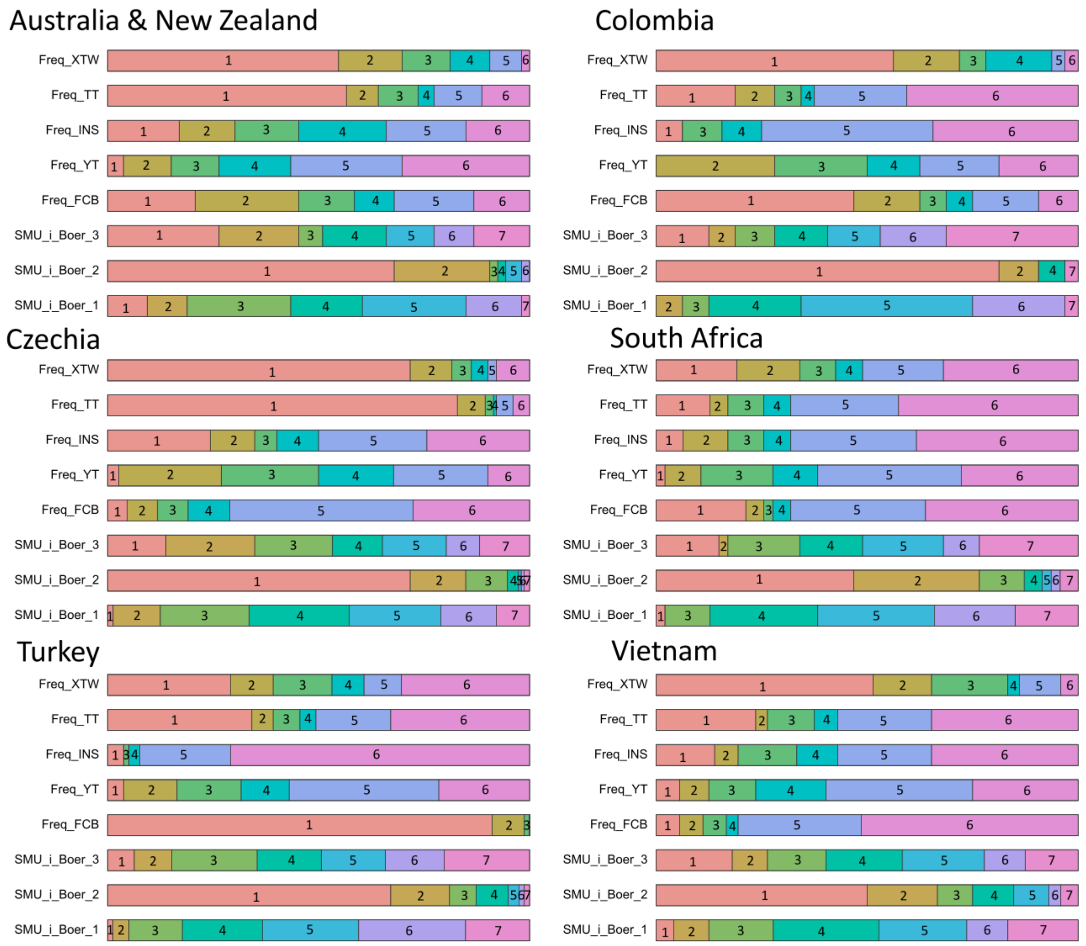

Fig S4. Distribution of answers to the questions underlying the final SMU_score (note that Freq_XTW and Freq_FCB were not included as their factor loadings were low). Numbers in the fields correspond to the response options on 6-point Likert scales (rows 1–5) and 7-point Likert scales (rows 6–8), respectively. The wider the field of a given colour, the more frequently it was selected by raters in the sample. If a number is missing, no one chose that answer. XTW = X [Twitter], TT = TikTok, INS = Instagram, YT = YouTube, FCB = Facebook, SMU_i_Boer = question on social media use intensity taken from Boer et al. (2022).

Part III. Results:

Attr = Rated attractiveness
Dom = Rated dominance
Trustw = Rated trustworthiness

GMM = Geometric Morphometrics

Subsection 1: ATTR/TRUSTW/DOM: Model Comparison

“Zero stage”: Predictions based on the models with just one response variable: Attractiveness, trustworthiness, or dominance. The predictions should be nearly identical to the predictions presented in the article. The only thing that changes is that instead of using one model with three

dependent variables, fitted as if they were from one 3D-multinormal distribution, we fit three models, each with just one of the scales as the dependent variable.

(a) Model with varying term on the level of faces (Attr/Trustw/Dom_long_F/M):

```
Attr_long_F <- ulam(alist(

  # Multivariate normal for Attractiveness
  AtrRating ~ normal(muA, sigma_A),

  # Mean structure for Attractiveness
  muA <- z_rNV_A[rater] * sigma_rater_A
  + f_per_group_A[face, FSMUi] # per face - per culture
  + (aA + f_per_group_pr_A[FSMUi,1])
  + (b_age_A + f_per_group_pr_A[FSMUi, 2]) * Age
  + (b_dist_A + f_per_group_pr_A[FSMUi, 3]) * dist
  + (b_FA_A + f_per_group_pr_A[FSMUi, 4]) * FA
  + (b_sshd_A + f_per_group_pr_A[FSMUi, 5]) * sshd
  + (b_L_A + f_per_group_pr_A[FSMUi,6]) * L,

  # Priors for Attractiveness intercept
  aA ~ dnorm(0, 0.5), # Attractiveness

  # Priors for Attractiveness and slopes
  b_age_A ~ dnorm(0, 0.3),
  b_dist_A ~ dnorm(0, 0.3),
  b_FA_A ~ dnorm(0, 0.3),
  b_sshd_A ~ dnorm(0, 0.3),
  b_L_A ~ dnorm(0, 0.3),

  # Non-centered parameterization for rater effects
  z_rNV_A[rater] ~ dnorm(0, 1), # Attractiveness latent variable

  sigma_rater_A ~ dexp(1), # Attractiveness

  gq> vector[rater]:a_rNV_A <<- aA + z_rNV_A * sigma_rater_A, # Generate rater effects for
Attractiveness

  # Varying effects for Attractiveness morpho-predictors
  transpars> matrix[FSMUi, 6]:f_per_group_pr_A <- compose_noncentered(sigma_pr_A, L_Rho_pr_A,
z_pr_A),
  cholesky_factor_corr[6]:L_Rho_pr_A ~ lkj_corr_cholesky(2),
  matrix[6, FSMUi]:z_pr_A ~ normal(0, 1),
  vector[6]:sigma_pr_A ~ dexp(1),

  gq> matrix[6, 6]:Rho_pr_A <<- Chol_to_Corr(L_Rho_pr_A),

  # Priors for the multivariate normal distribution for face intercepts across groups
  transpars> matrix[face, 12]:f_per_group_A <- compose_noncentered(sigma_FSMUi_A,
L_Rho_FSMUi_A, z_FSMUi_A),
  cholesky_factor_corr[12]:L_Rho_FSMUi_A ~ lkj_corr_cholesky(2),
  matrix[12, face]:z_FSMUi_A ~ normal(0, 1),
  vector[12]:sigma_FSMUi_A ~ dexp(1),

  gq> matrix[12, 12]:Rho_FSMUi_A <<- Chol_to_Corr(L_Rho_FSMUi_A),

  sigma_A ~ dexp(1)

), data=D_AT_F, iter=700, sample=T, cores=14, chains=14, log_lik=T)
```

We also run the same model with the parts in **bold** deleted (Attr/Trustw/Dom_short_F/M). The two models were compared using WAIC. The same models were also fitted for trustworthiness and dominance.

WAIC [out of sample predictive accuracy; "model comparison"] – **attractiveness**

(F):

	WAIC	SE	dWAIC	dSE	pWAIC	weight
Attr_long_F	49494.3	248.88	0	NA	1042.9	1
Attr_short_F	55666.3	238.52	6172	153.23	446.1	0

(M):

WAIC SE dWAIC dSE pWAIC weight

```
Attr_long_M  42128.1 232.77    0.0     NA 932.7      1
Attr_short_M 47265.4 224.66 5137.3 141.72 435.7      0
```

WAIC – trustworthiness

(F):

```
WAIC     SE  dWAIC    dSE pWAIC weight
Trustw_long_F  55418.7 230.32    0.0     NA 941.2      1
Trustw_short_F 58215.4 223.08 2796.7 106.55 438.2      0
```

(M):

```
                  WAIC     SE  dWAIC    dSE pWAIC weight
Trustw_long_M  46600.1 206.34    0.0     NA 884.4      1
Trustw_short_M 49333.2 198.09 2733.1 103.67 430.7      0
```

WAIC – dominance

(F):

```
               WAIC     SE  dWAIC   dSE pWAIC weight
Dom_long_F  57766.6 221.47    0.0    NA 937.3      1
Dom_short_F 59748.2 212.56 1981.6 91.98 433.0      0
```

(M):

```
               WAIC     SE  dWAIC   dSE pWAIC weight
Dom_long_M  48002.9 210.11    0.0    NA 857.9      1
Dom_short_M 50223.0 199.10 2220.2 97.72 428.5      0
```

In all cases, the models with varying terms for each face (estimating also the correlation between different groups' ratings) had better out-of-sample predictive accuracy. Models that try to assign all the variance just to the level of the group (not face) perform *worse*. Results of the "long" models are reported below.

Note: A sampling problem experienced during posterior evaluation:

Posterior sampling was conducted using Stan via the cmdstanr interface on an Intel® Core™ i9-12900K processor. With 16 parallel chains distributed across 16 cores, we occasionally observed that a single chain produced divergent behaviour, leading to an imbalanced distribution with a noticeable skew toward negative values.

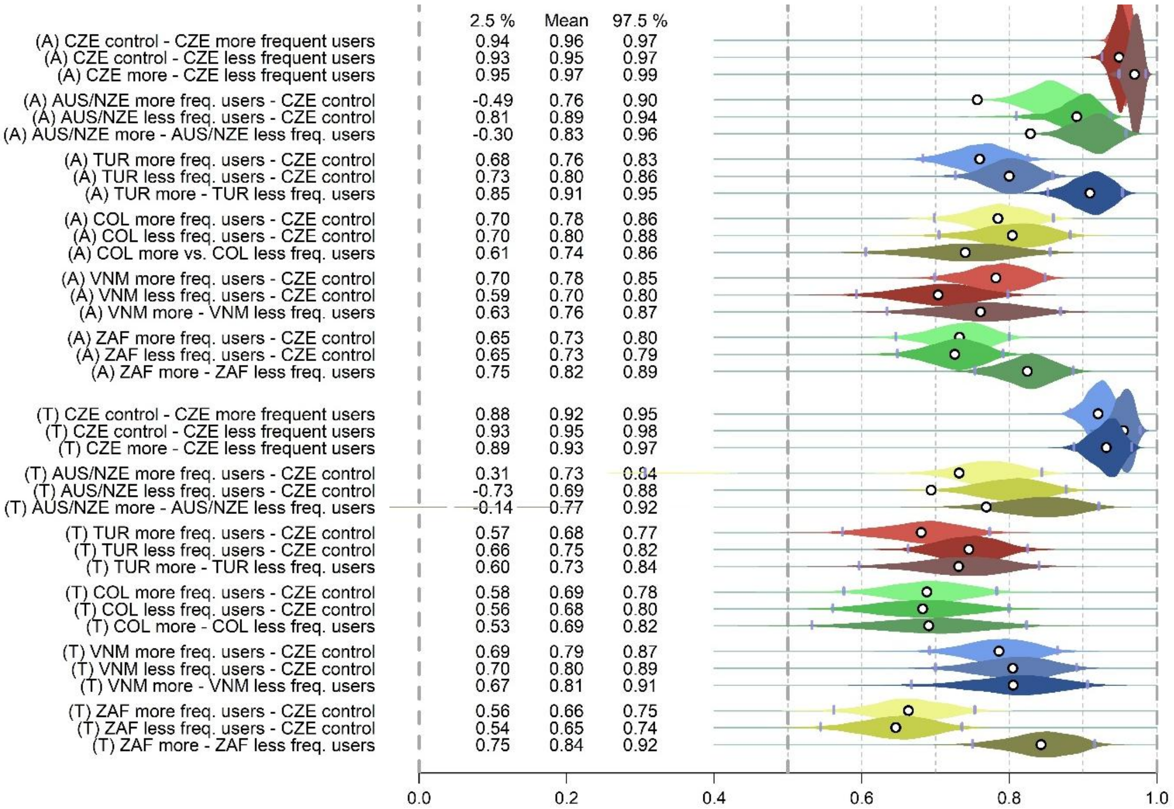

Figure S5. Divergent behaviour, likely induced by just one sample, since the bulk is otherwise precisely framed (plus see the description below).

This problem was experienced only when we assigned one chain to every physical core (but mind there are 24 threads altogether). We could only speculate what is the reason of such behaviour. Importantly, however, this issue was not experienced when we used smaller number of chains (14 for a 16-core CPU). This way, there is clearly a computer-related problem that prevents large model under certain setting from being sampled correctly, which is quite an issue, given that such models last long and usually are desperately awaited by the researchers whose deadline for submission is quickly approchaing. If you possess an explanation for the problem shown above, do not hesitate to contact me at vfiala@umk.pl.

Subsection 1.2. Models with attractiveness, trustworthiness, and dominance as the sole response variable: model predictions

In each model, there was a single dependent variable: Attractiveness, Trustworthiness, or Dominance. We also demonstrate that the predictions of these three separate models do not differ substantially from those obtained when all three scales are included in the same model.

WAIC cannot be directly computed when fitting a model with more than one dependent variable using the ulam() function from the rethinking package. While this limitation could be bypassed by fitting the model directly in Stan within R, the principal investigator preferred to avoid this approach, as it would require performing all subsequent steps without the predefined functions provided by rethinking. Furthermore, with three intercorrelated dependent variables, WAIC values based solely on estimated parameters may be unreliable. This is because part of the correlation among the scales is not explicitly modelled, other than by treating all rated variables as a single three-dimensional normal distribution. The figures below indicate that whether the three scales are modelled together or separately, the resulting predictions are nearly identical.

1.2.1. Figure S6 - analogue of Figure 3 in the article "correlation heatmaps"

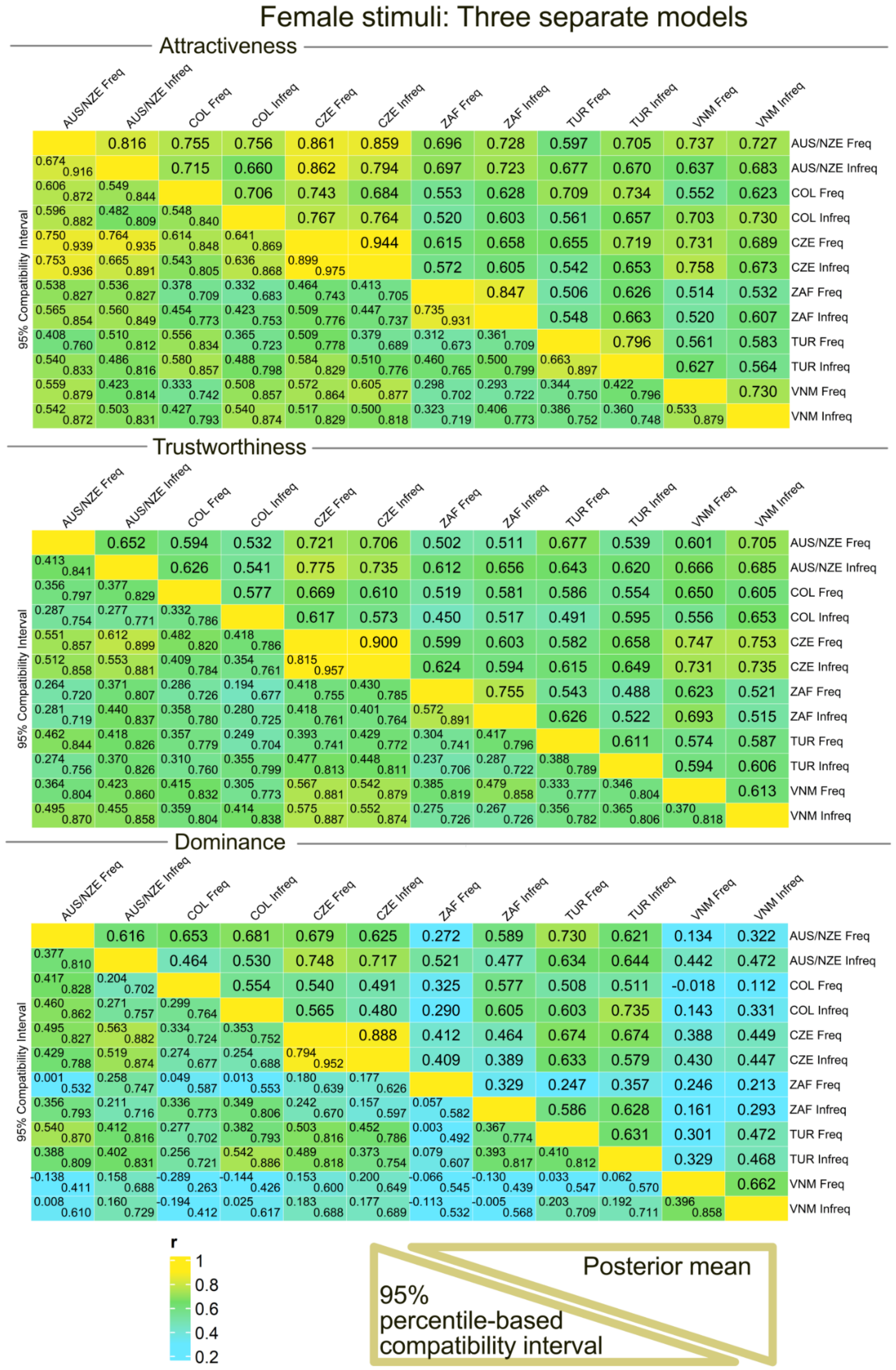

Figure S6. Posterior distributions of correlation coefficients: association between ratings in different samples (Freq = users < median in social media use frequency; Infreq = users > the median). Female stimuli. Predictions based on three separate models. Country abbreviations (CZE [Czechia], AUS/NZE [Australia/New Zealand], TUR [Turkey], COL [Colombia], VNM [Vietnam], ZAF [South Africa]) correspond to three-letter country codes (ISO 3166-1 alpha-3).

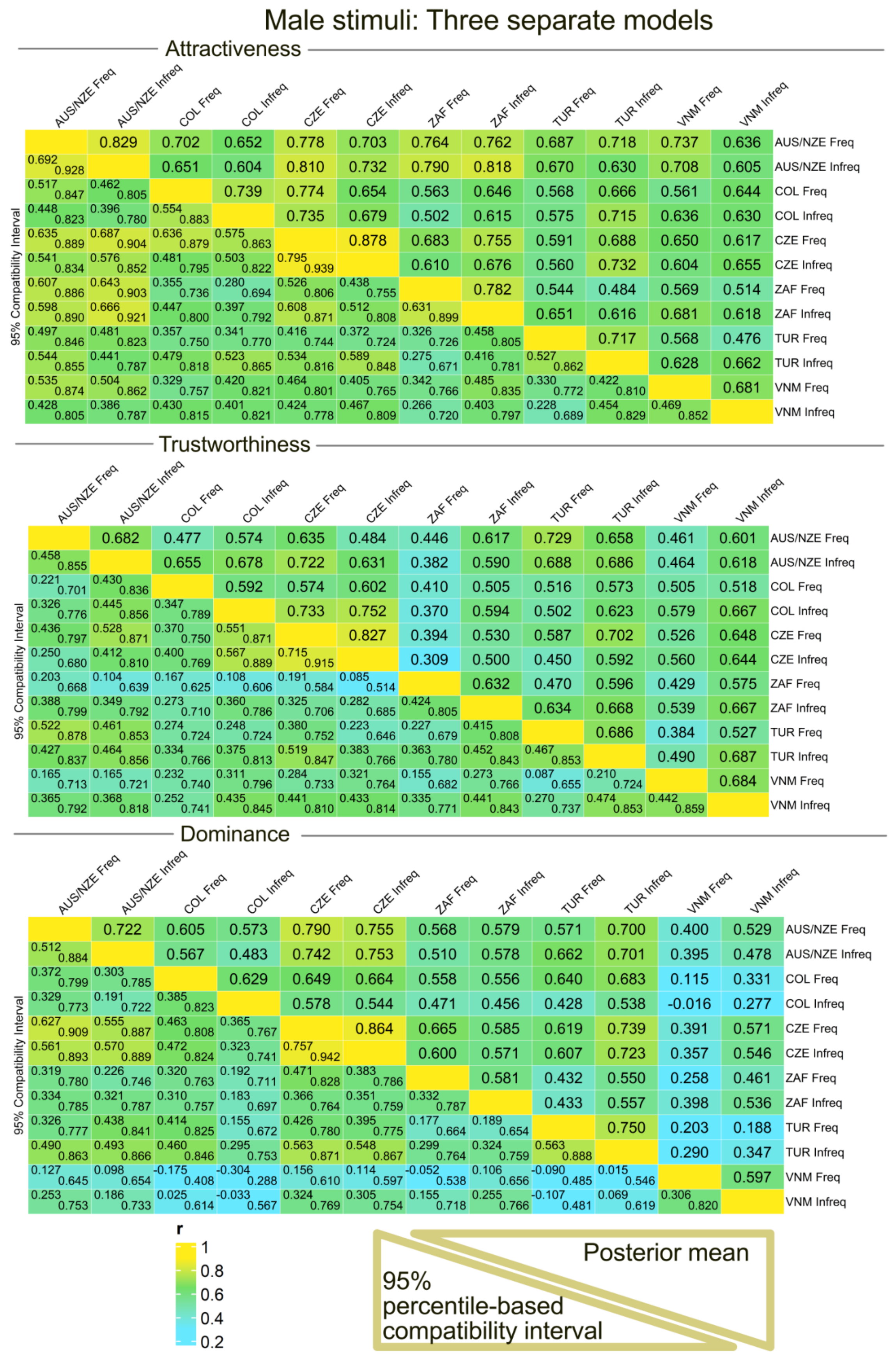

Figure S7. Correlation heatmaps, male stimuli. Layout of this figure is identical to Figure S4.

1.2.2. An analogue of Fig. 3 in the article “Means & slopes: Females”

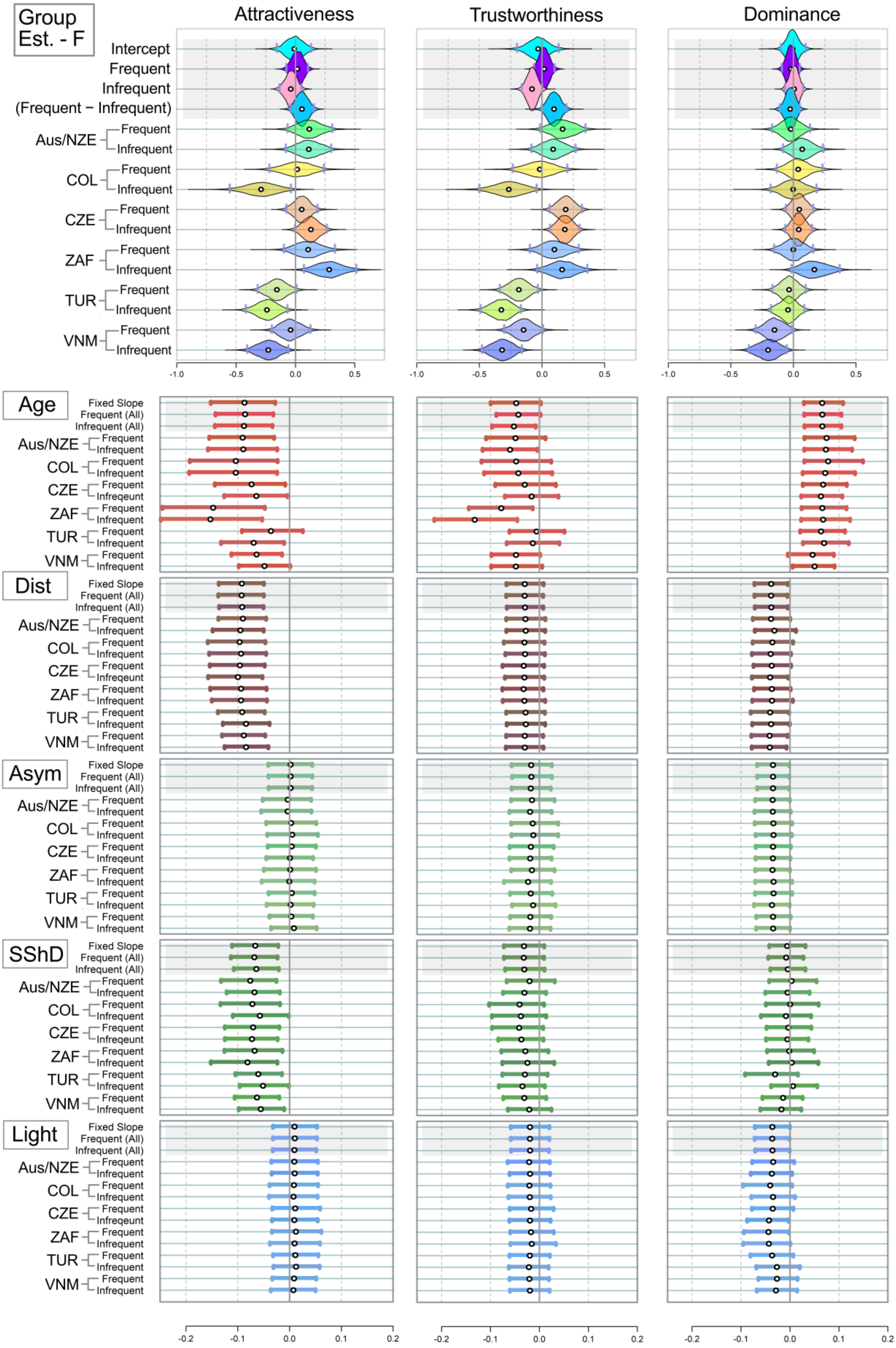

Figure S8. Mean estimated ratings of attractiveness, trustworthiness, and dominance for the twelve female stimuli samples (upper panel). Estimated effects of morphometric predictors and age on the perceived characteristics (lower panel). This figure is an analogue of Figure 3, based on separate models with a single dependent variable (attractiveness, trustworthiness, or dominance). White point = mean estimate; grey vertical bars = bounds of the 95% percentile-based compatibility intervals. Dist = distinctiveness; Asym = facial asymmetry; SShD = sexual shape dimorphism; Light = skin lightness. Group Est. = group estimates, representing the average ratings assigned to faces by frequent and infrequent users within each population. As in all other figures, the country abbreviations correspond to three-letter ISO 3166-1 alpha-3 country codes.

1.2.3. An analogue of Figure 4 in the article “Means & slopes: Males”

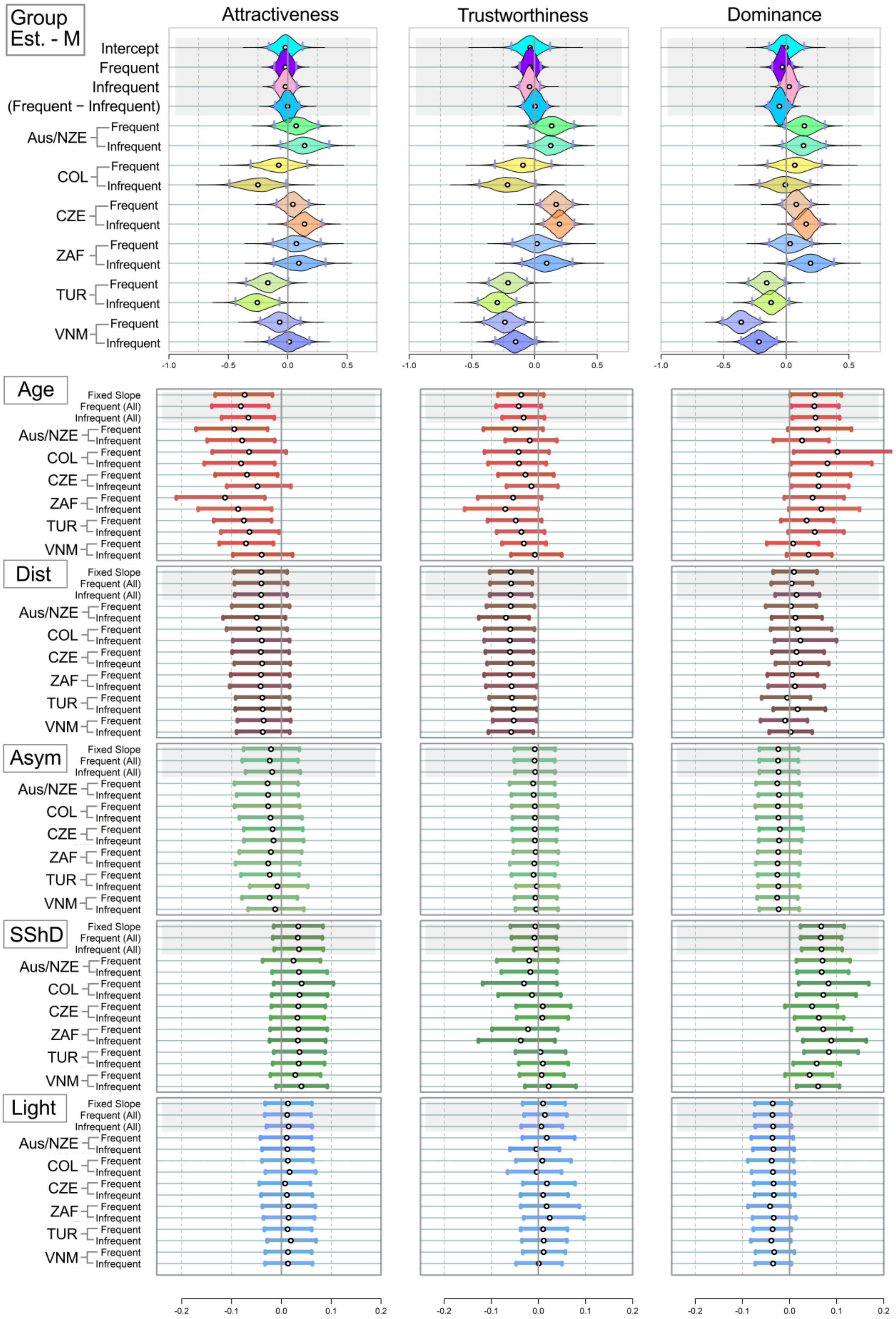

Figure S9. Mean estimated ratings of attractiveness, trustworthiness, and dominance, Males.

Subsection 1.3. Model with the three response variables - attractiveness, trustworthiness, and dominance (main model): supplementary information on the model transcription and predictions

Model transcription:

```
ATD_model <- ulam(alist(

  # Multivariate normal for Trustworthiness and Attractiveness
  c(AtrRating, TrustwRating, DomRating) ~ multi_normal(c(muA, muT, muD), Rho_Scales,
sigma_Scales),


  # ATTRACTIVENESS
  muA <- z_rNV_A[rater] * sigma_rater_A
  + f_per_group_A[face, FSMUi] # per face - per culture
  + (aA + f_per_group_pr_A[FSMUi,1])
  + (b_age_A + f_per_group_pr_A[FSMUi, 2]) * Age
  + (b_dist_A + f_per_group_pr_A[FSMUi, 3]) * dist
  + (b_FA_A + f_per_group_pr_A[FSMUi, 4]) * FA
  + (b_sshd_A + f_per_group_pr_A[FSMUi, 5]) * sshd
  + (b_L_A + f_per_group_pr_A[FSMUi,6]) * L,

  aA ~ dnorm(0, 0.5),  #

  b_age_A ~ dnorm(0, 0.3),
  b_dist_A ~ dnorm(0, 0.3),
  b_FA_A ~ dnorm(0, 0.3),
  b_sshd_A ~ dnorm(0, 0.3),
  b_L_A ~ dnorm(0, 0.3),

  # Non-centered parameterization for rater effects
  z_rNV_A[rater] ~ dnorm(0, 1),  #  latent variable

  sigma_rater_A ~ dexp(1),  # Attractiveness

  gq> vector[rater]:a_rNV_A <<- aA + z_rNV_A * sigma_rater_A,  # Generate rater effects for
Attractiveness

  # Varying effects for Attractiveness morpho-predictors
  transpars> matrix[FSMUi, 6]:f_per_group_pr_A <- compose_noncentered(sigma_pr_A, L_Rho_pr_A,
z_pr_A),
  cholesky_factor_corr[6]:L_Rho_pr_A ~ lkj_corr_cholesky(2),
  matrix[6, FSMUi]:z_pr_A ~ normal(0, 1),
  vector[6]:sigma_pr_A ~ dexp(1),

  gq> matrix[6, 6]:Rho_pr_A <<- Chol_to_Corr(L_Rho_pr_A),

  # Priors for the multivariate normal distribution for face intercepts across groups
  transpars> matrix[face, 12]:f_per_group_A <- compose_noncentered(sigma_FSMUi_A,
L_Rho_FSMUi_A, z_FSMUi_A),
  cholesky_factor_corr[12]:L_Rho_FSMUi_A ~ lkj_corr_cholesky(2),
  matrix[12, face]:z_FSMUi_A ~ normal(0, 1),
  vector[12]:sigma_FSMUi_A ~ dexp(1),

  gq> matrix[12, 12]:Rho_FSMUi_A <<- Chol_to_Corr(L_Rho_FSMUi_A),


  # TRUSTWORTHINESS
  muT <- z_rNV_T[rater] * sigma_rater_T
  + f_per_group_T[face, FSMUi] # per face - per culture
  + (aT + f_per_group_pr_T[FSMUi, 1])
  + (b_age_T + f_per_group_pr_T[FSMUi, 2]) * Age
  + (b_dist_T + f_per_group_pr_T[FSMUi, 3]) * dist
  + (b_FA_T + f_per_group_pr_T[FSMUi, 4]) * FA
  + (b_sshd_T + f_per_group_pr_T[FSMUi, 5]) * sshd
  + (b_L_T + f_per_group_pr_T[FSMUi, 6]) * L,

  aT ~ dnorm(0, 0.5),

  b_age_T ~ dnorm(0, 0.3),
  b_dist_T ~ dnorm(0, 0.3),
  b_FA_T ~ dnorm(0, 0.3),
  b_sshd_T ~ dnorm(0, 0.3),
  b_L_T ~ dnorm(0, 0.3),

  z_rNV_T[rater] ~ dnorm(0, 1),  # Trustworthiness latent variable

  sigma_rater_T ~ dexp(1),

  gq> vector[rater]:a_rNV_T <<- aT + z_rNV_T * sigma_rater_T,  # Generate rater effects for
Trustworthiness
```

```
  # Varying effects for Trustworthiness morpho-predictors
  transpars> matrix[FSMUi, 6]:f_per_group_pr_T <- compose_noncentered(sigma_pr_T, L_Rho_pr_T, 
z_pr_T),
  cholesky_factor_corr[6]:L_Rho_pr_T ~ lkj_corr_cholesky(2),
  matrix[6, FSMUi]:z_pr_T ~ normal(0, 1),
  vector[6]:sigma_pr_T ~ dexp(1),

  gq> matrix[6, 6]:Rho_pr_T <<- Chol_to_Corr(L_Rho_pr_T),

  transpars> matrix[face, 12]:f_per_group_T <- compose_noncentered(sigma_FSMUi_T, 
L_Rho_FSMUi_T, z_FSMUi_T),
  cholesky_factor_corr[12]:L_Rho_FSMUi_T ~ lkj_corr_cholesky(2),
  matrix[12, face]:z_FSMUi_T ~ normal(0, 1),
  vector[12]:sigma_FSMUi_T ~ dexp(1),

  gq> matrix[12, 12]:Rho_FSMUi_T <<- Chol_to_Corr(L_Rho_FSMUi_T),


  # DOMINANCE
  muD <- z_rNV_D[rater] * sigma_rater_D
  + f_per_group_D[face, FSMUi] # per face - per culture
  + (aD + f_per_group_pr_D[FSMUi,1])
  + (b_age_D + f_per_group_pr_D[FSMUi, 2]) * Age
  + (b_dist_D + f_per_group_pr_D[FSMUi, 3]) * dist
  + (b_FA_D + f_per_group_pr_D[FSMUi, 4]) * FA
  + (b_sshd_D + f_per_group_pr_D[FSMUi, 5]) * sshd
  + (b_L_D + f_per_group_pr_D[FSMUi, 6]) * L,

  aD ~ dnorm(0, 0.5),

  b_age_D ~ dnorm(0, 0.3),
  b_dist_D ~ dnorm(0, 0.3),
  b_FA_D ~ dnorm(0, 0.3),
  b_sshd_D ~ dnorm(0, 0.3),
  b_L_D ~ dnorm(0, 0.3),

  # Non-centered parameterization for rater effects
  z_rNV_D[rater] ~ dnorm(0, 1),  #  latent variable

  sigma_rater_D ~ dexp(1),  # Dominance

  gq> vector[rater]:a_rNV_D <<- aD + z_rNV_D * sigma_rater_D,

  transpars> matrix[FSMUi, 6]:f_per_group_pr_D <- compose_noncentered(sigma_pr_D, L_Rho_pr_D, 
z_pr_D),
  cholesky_factor_corr[6]:L_Rho_pr_D ~ lkj_corr_cholesky(2),
  matrix[6, FSMUi]:z_pr_D ~ normal(0, 1),
  vector[6]:sigma_pr_D ~ dexp(1),

  gq> matrix[6, 6]:Rho_pr_D <<- Chol_to_Corr(L_Rho_pr_D),

  # Priors for the multivariate normal distribution for face intercepts across groups
  transpars> matrix[face, 12]:f_per_group_D <- compose_noncentered(sigma_FSMUi_D, 
L_Rho_FSMUi_D, z_FSMUi_D),
  cholesky_factor_corr[12]:L_Rho_FSMUi_D ~ lkj_corr_cholesky(2),
  matrix[12, face]:z_FSMUi_D ~ normal(0, 1),
  vector[12]:sigma_FSMUi_D ~ dexp(1),

  gq> matrix[12, 12]:Rho_FSMUi_D <<- Chol_to_Corr(L_Rho_FSMUi_D),


  # Common terms
  Rho_Scales ~ lkj_corr(2),
  sigma_Scales ~ dexp(1)

), data=data, iter=700, sample=T, cores=14, chains=14)
```

Below, we provide additional tables and extended versions of those included in the article.

<u>Selected comparisons:</u>

Table S7. Correlation comparisons: extended

Female stimuli	Attractiveness	Trustworthiness	Dominance
Mean: Czech Users	0.95 [0.90; 0.98]	0.90 [0.82; 0.96]	0.89 [0.80; 0.96]
Mean: All other Users	0.69 [0.64; 0.75]	0.64 [0.57; 0.69]	0.48 [0.41; 0.55]
Difference	0.25 [0.19; 0.31]	0.26 [0.17; 0.35]	0.41 [0.31; 0.51]
Male stimuli	Attractiveness	Trustworthiness	Dominance

Mean: Czech Users	0.89 [0.82; 0.95]	0.84 [0.73; 0.92]	0.87 [0.76; 0.94]
Mean: All other Users	0.68 [0.62; 0.73]	0.59 [0.52; 0.65]	0.54 [0.47; 0.61]
Difference	0.21 [0.13; 0.29]	0.25 [0.13; 0.35]	0.33 [0.21; 0.43]
Female stimuli	Attractiveness	Trustworthiness	Dominance
Mean: CZ+AUS/NZE Users	0.87 [0.81; 0.92]	0.77 [0.67; 0.85]	0.71 [0.59; 0.80]
Mean: All other Users	0.68 [0.62; 0.74]	0.63 [0.56; 0.69]	0.46 [0.39; 0.53]
Difference	0.19 [0.13; 0.25]	0.14 [0.04; 0.23]	0.24 [0.14; 0.35]
Male stimuli	Attractiveness	Trustworthiness	Dominance
Mean: CZ+AUS/NZE Users	0.80 [0.73; 0.87]	0.68 [0.56; 0.77]	0.77 [0.68; 0.86]
Mean: All other Users	0.67 [0.61; 0.73]	0.58 [0.51; 0.65]	0.52 [0.45; 0.59]
Difference	0.13 [0.06; 0.20]	0.09 [−0.01; 0.19]	0.26 [0.16; 0.36]
Female stimuli	Attractiveness	Trustworthiness	Dominance
Mean: Prolific (AUS/NZE+RSA)	0.70 [0.62; 0.78]	0.66 [0.54; 0.76]	0.61 [0.48; 0.72]
Mean: All other Users	0.70 [0.64; 0.75]	0.64 [0.58; 0.70]	0.47 [0.40; 0.54]
Difference	0.01 [−0.07; 0.08]	0.02 [−0.08; 0.12]	0.13 [0.02; 0.25]
Male stimuli	Attractiveness	Trustworthiness	Dominance
Mean: Prolific (AUS/NZE+RSA)	0.75 [0.66; 0.83]	0.67 [0.54; 0.77]	0.59 [0.46; 0.70]
Mean: All other Users	0.68 [0.61; 0.73]	0.58 [0.51; 0.65]	0.54 [0.47; 0.61]
Difference	0.07 [−0.01; 0.16]	0.08 [−0.03; 0.19]	0.05 [−0.06; 0.16]

Part IV. Model with the three response variables (attractiveness, trustworthiness, dominance), raters in the 25% band around median SMU_score excluded

Exclusion criteria: We computed the underlying score based on questions Q1–Q8, whose factor loadings are described above. Participants were relatively evenly distributed across the full range of this score, particularly when examining country-specific distributions (see Figure S3, provided earlier in this document). However, including participants with scores around the median could reduce the ability to distinguish first impressions between the two groups. Therefore, we excluded these participants from the analysis and examined whether the model predictions changed—for instance, whether there were systematic differences in agreement between the above- and below-median groups.

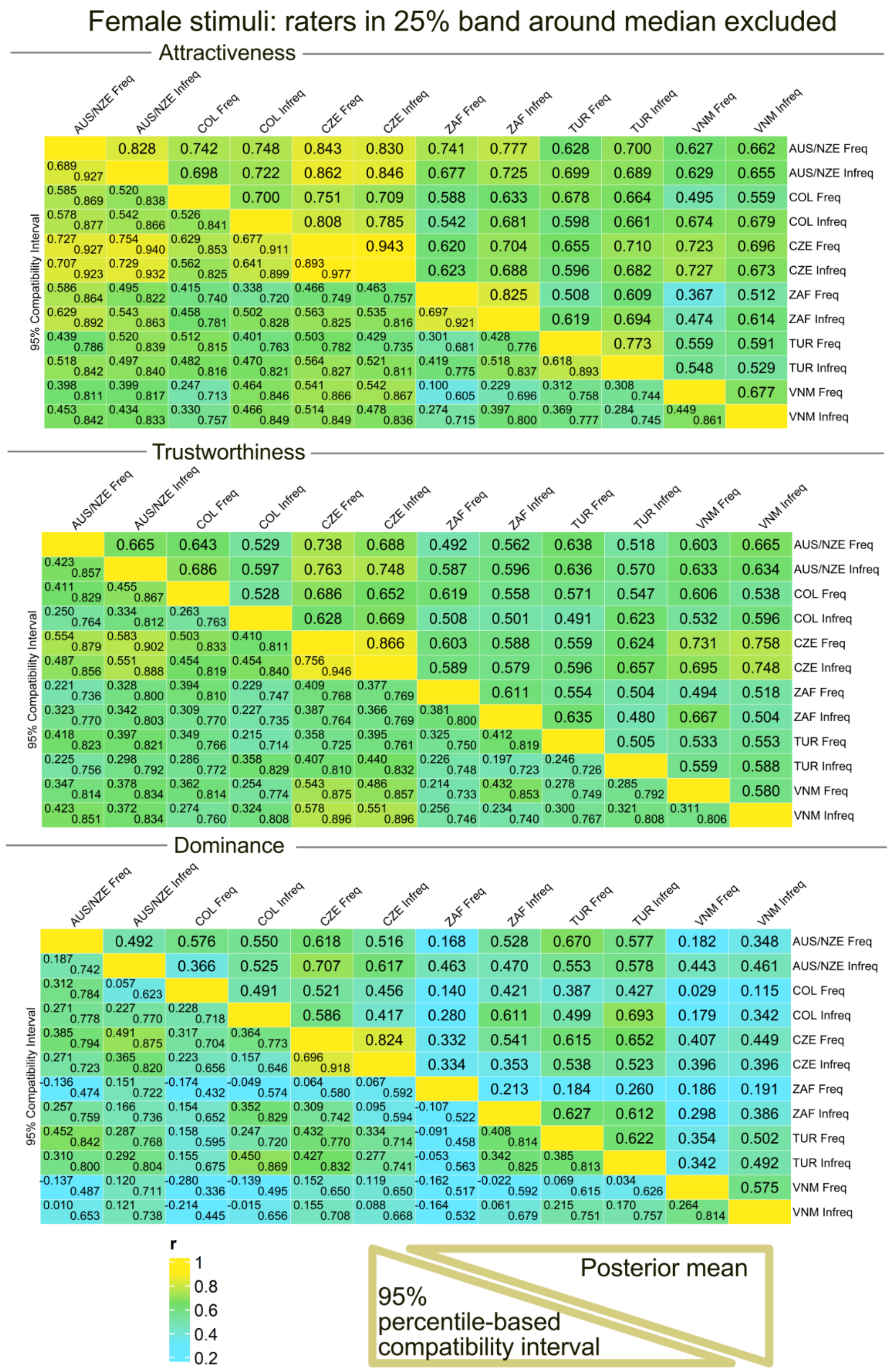

Figure S10. Posterior distributions of correlation coefficients showing associations between ratings across samples. Freq = users above the median social media use frequency; Infreq = users below the median. Female stimuli only. Predictions are based on a model with three dependent variables (Attractiveness, Trustworthiness, and Dominance), with participants within the 25% band around the median SMU_score excluded.

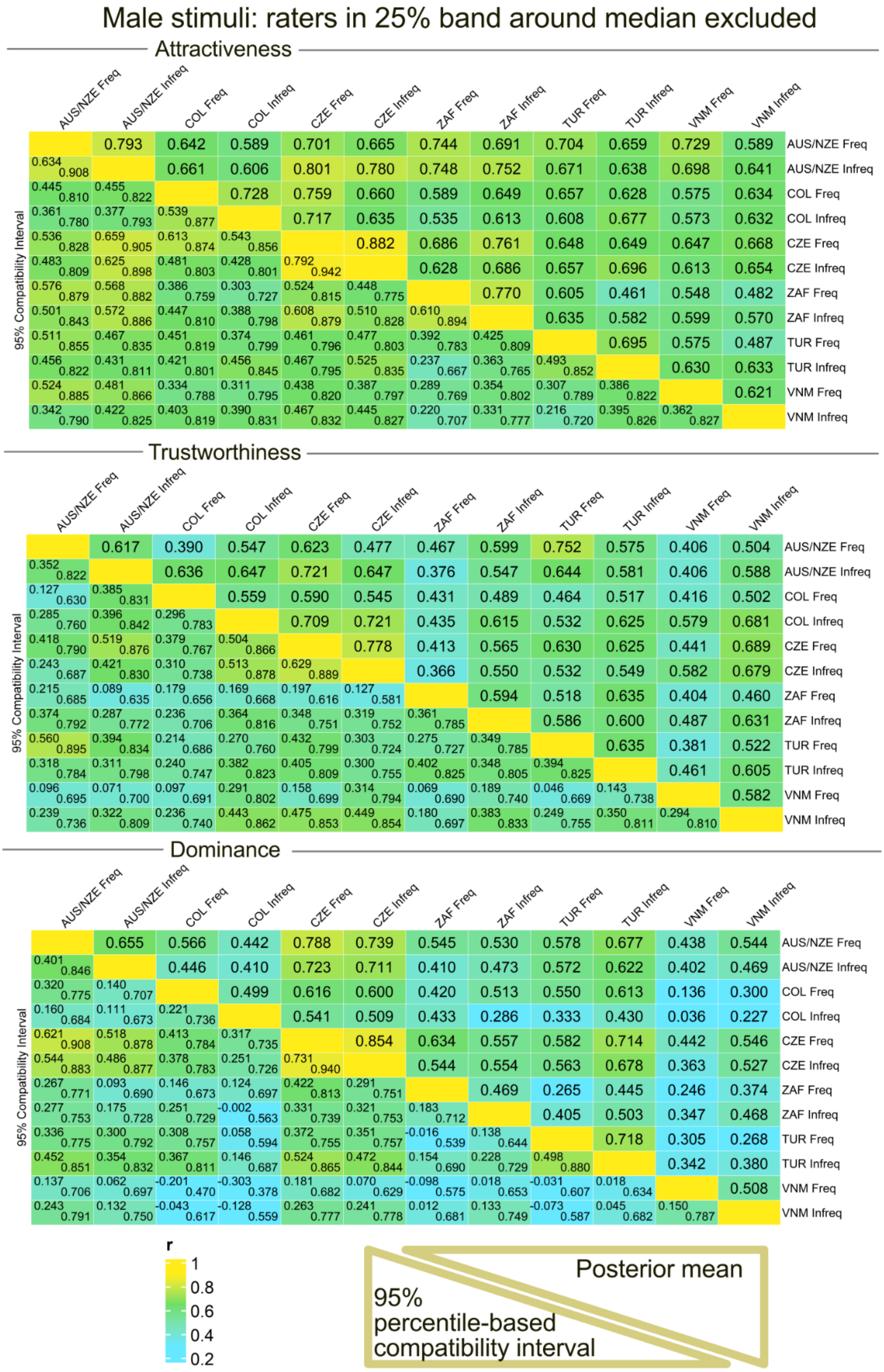

Figure S11. Posterior distributions of correlation coefficients showing associations between ratings across samples, male stimuli. Predictions are based on a model with three dependent variables (Attractiveness, Trustworthiness, and Dominance), with participants within the 25% band around the median SMU_score excluded.

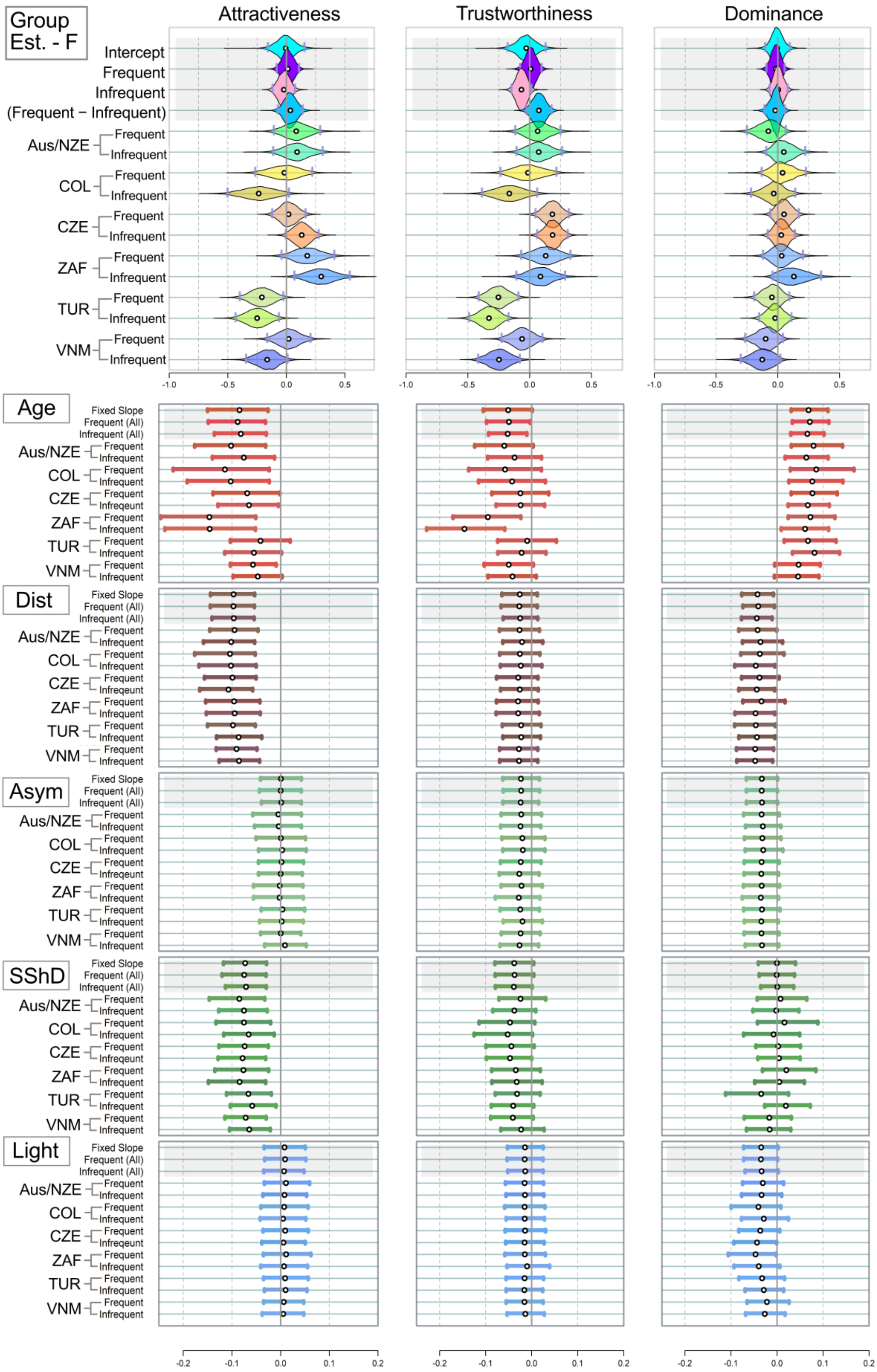

Figure S12. Mean estimated ratings of attractiveness, trustworthiness, and dominance in female stimuli. Analogue of Figure 3, participants in 25% SMU_score median band excluded. White point = Mean of the estimate; Grey Vertical Bars = Border of 95% percentile-based Compatibility Intervals. Dist = distinctiveness; Asym = facial asymmetry; SShD = Sexual Shape Dimorphism;

Light = Skin lightness. Group Est. = Group Estimates - average ratings assigned to the faces in the group of frequent and infrequent users per population.

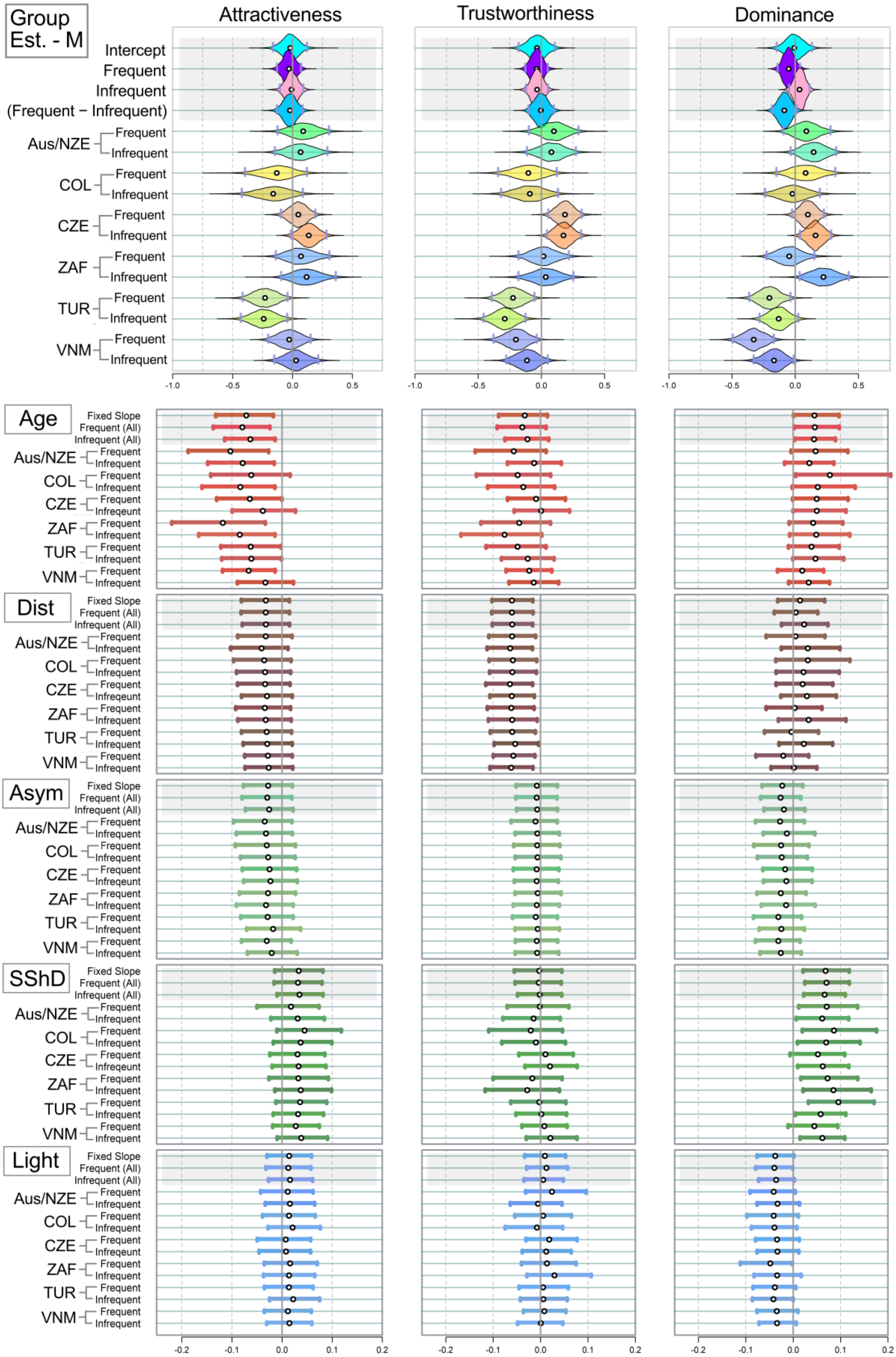

Figure S13. Mean estimated ratings of attractiveness, trustworthiness, and dominance in male

stimuli. Analogue of Figure 3, run on models with the three dependent variables (attractiveness, trustworthiness, and dominance), participants in 25% band around median SMU_score excluded.

Below, we characterise the level of agreement within and between selected populations.

Table S8. Correlation comparisons. Mean stands for "Mean correlation". Participants within 25% band around median in SMU_score excluded.

Female stimuli	Attractiveness	Trustworthiness	Dominance
Mean within cultures	0.79 [0.73; 0.85]	0.63 [0.53; 0.71]	0.54 [0.43; 0.64]
Mean across cultures	0.66 [0.60; 0.72]	0.60 [0.53; 0.67]	0.44 [0.36; 0.52]
Difference	*0.13 [0.07; 0.19]*	*0.03 [−0.05; 0.11]*	*0.10 [−0.00; 0.20]*
Mean: Frequent users	0.63 [0.55; 0.71]	0.60 [0.51; 0.69]	0.60 [0.51; 0.69]
Mean: Infrequent users	0.69 [0.61; 0.76]	0.61 [0.51; 0.70]	0.50 [0.39; 0.60]
Difference	*−0.05 [−0.15; 0.04]*	*−0.00 [−0.12; 0.12]*	*0.11 [−0.03; 0.25]*
Male stimuli	Attractiveness	Trustworthiness	Dominance
Mean within cultures	0.75 [0.67; 0.81]	0.63 [0.52; 0.72]	0.62 [0.51; 0.71]
Mean across cultures	0.63 [0.57; 0.70]	0.54 [0.47; 0.62]	0.48 [0.40; 0.56]
Difference	*0.11 [0.05; 0.18]*	*0.08 [−0.00; 0.17]*	*0.14 [0.04; 0.24]*
Mean: Frequent users	0.65 [0.57; 0.73]	0.49 [0.39; 0.59]	0.47 [0.37; 0.58]
Mean: Infrequent users	0.65 [0.57; 0.74]	0.62 [0.52; 0.71]	0.48 [0.37; 0.59]
Difference	*0.00 [−0.11; 0.11]*	*−0.13 [−0.25; −0.00]*	*−0.01 [−0.14; 0.13]*

Table S9. "Difference of differences" – Are average differences in mean estimates within a population always smaller than average differences between samples from different populations? (Participants within 25% band around median in SMU_score excluded.)

Female stimuli	Attractiveness	Trustworthiness	Dominance
Mean difference within samples	0.15 [0.07; 0.25]	0.13 [0.06; 0.20]	0.09 [0.01; 0.18]
Mean difference between samples	0.24 [0.15; 0.33]	0.24 [0.18; 0.31]	0.12 [0.02; 0.20]
Difference of the two differences	0.09 [−0.00; 0.19]	0.12 [0.04; 0.20]	0.02 [−0.03; 0.09]
Male stimuli	Attractiveness	Trustworthiness	Dominance
Mean difference within samples	0.11 [0.05; 0.20]	0.11 [0.05; 0.18]	0.15 [0.07; 0.23]
Mean difference between samples	0.20 [0.10; 0.30]	0.22 [0.15; 0.29]	0.23 [0.16; 0.30]

Difference of the two differences	0.09 [−0.00; 0.19]	0.12 [0.03; 0.20]	0.08 [0.00; 0.16]

Part V: Travel abroad & SES during childhood

Table S10: Numbers of participants according to the frequency of their travel abroad (**Abroad**) and socioeconomic status (**SES**):

**Abroad**	CZ	VN	RSA	AUS/ NZ	COL	TUR
Often	29	6	2	1	1	5
Rather Often	50	3	2	5	2	7
Occasionaly	37	18	9	16	4	19
Rarely	19	9	6	10	1	9
Very rarely	17	15	10	21	12	8
Never	0	18	18	0	11	31
Nas	0	3	0	0	1	0
**SES**	CZ	VN	RSA	AUS/ NZ	COL	TUR
Rich	13	12	2	7	2	10
Upper CL	31	23	13	23	6	37
**Middle**	**62**	**7**	**23**	**11**	**10**	**15**
Lower CL	42	12	7	8	6	1
Poor	4	5	2	3	6	10
DoNotW2Answer	0	0	0	1	0	5
NAs	0	0	0	0	0	1

Abroad: Line shows where the sample was split in half („above“ / „below“), based on travel abroad frequency.

SES: Lines show where the sample was split in half („above“ / „below“), based socioeconmic status of the participants‘ family during their childhood. Upper line corresponds to „version_1“ (ver_1), lower line corresponds to „version_“2 (ver_2). It is not clear where the „Middle“ group should be added.

Abroad = Frequency of travel abroad.
SES = Socioeconomic status during childhood.
Both the variables are *self-reported*.
DoNotW2Answer = Participant marked she do not want to answer this presumably personal question.

Note that, in the case of SES, the results of the comparison below depend largely on how the 'Middle' group is allocated. In particular, when it is combined with the “Rich”, “Upper CL”, and “Middle” groups, the remaining groups become unbalanced, resulting in the 'below' group being much smaller than the 'above' group.

Part 3.1. Travel abroad

Model layout remains the same, but 'above' and 'below' now refer to travel abroad frequency. Participants were divided into two groups: those who reported travelling abroad 'Often', 'Rather Often', or 'Occasionally' were placed in the 'above' group, while those who reported travelling abroad 'Rarely', 'Very Rarely', or 'Never' were placed in the 'below' group (NAs excluded).

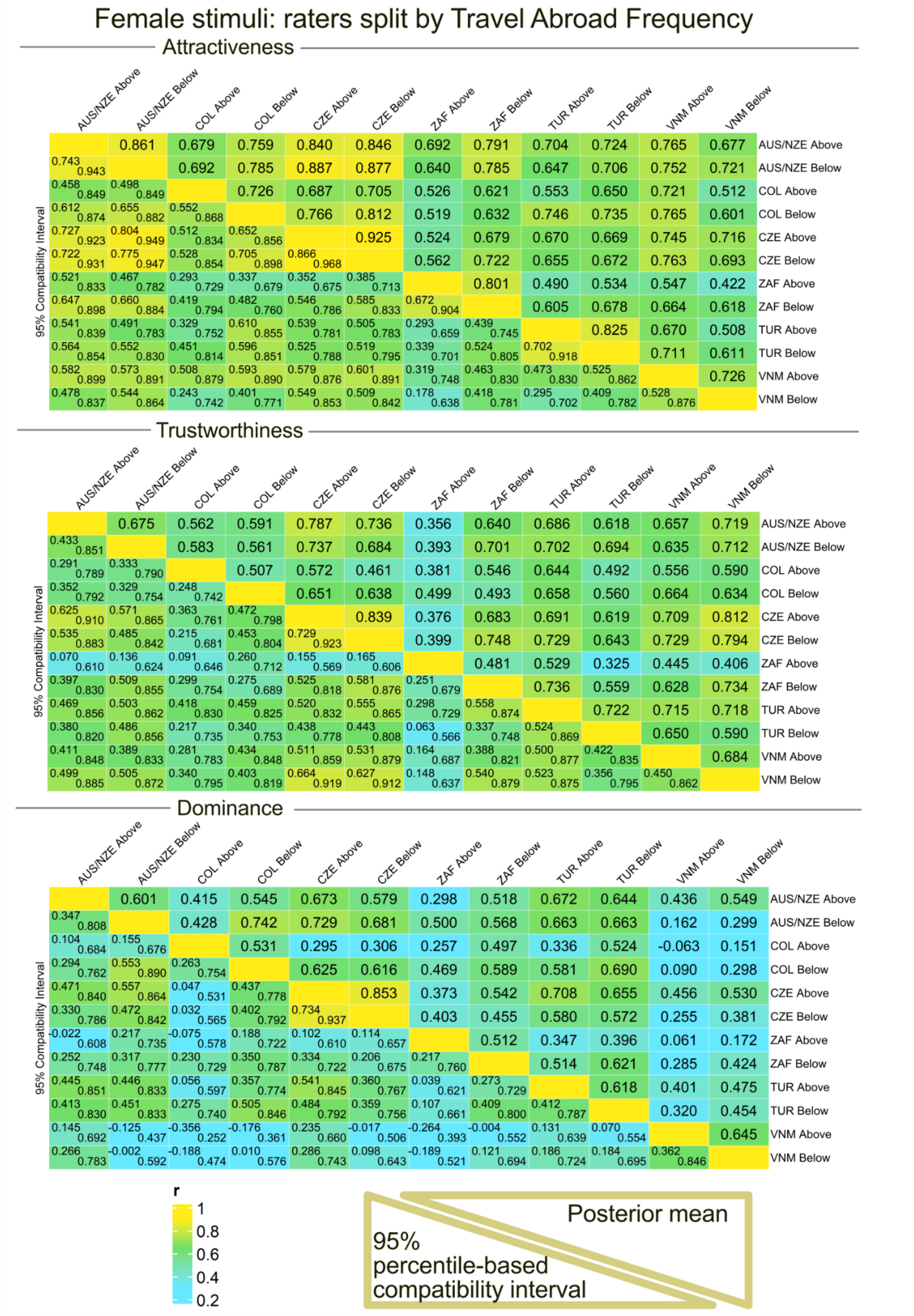

Figure S14. Participants from every country (the abbreviations are same as above), divided according to self-reported frequency of traveling abroad. Female stimuli. Please, mind that the numbers in some samples are low and unequal, which may impair the results' generalisability.

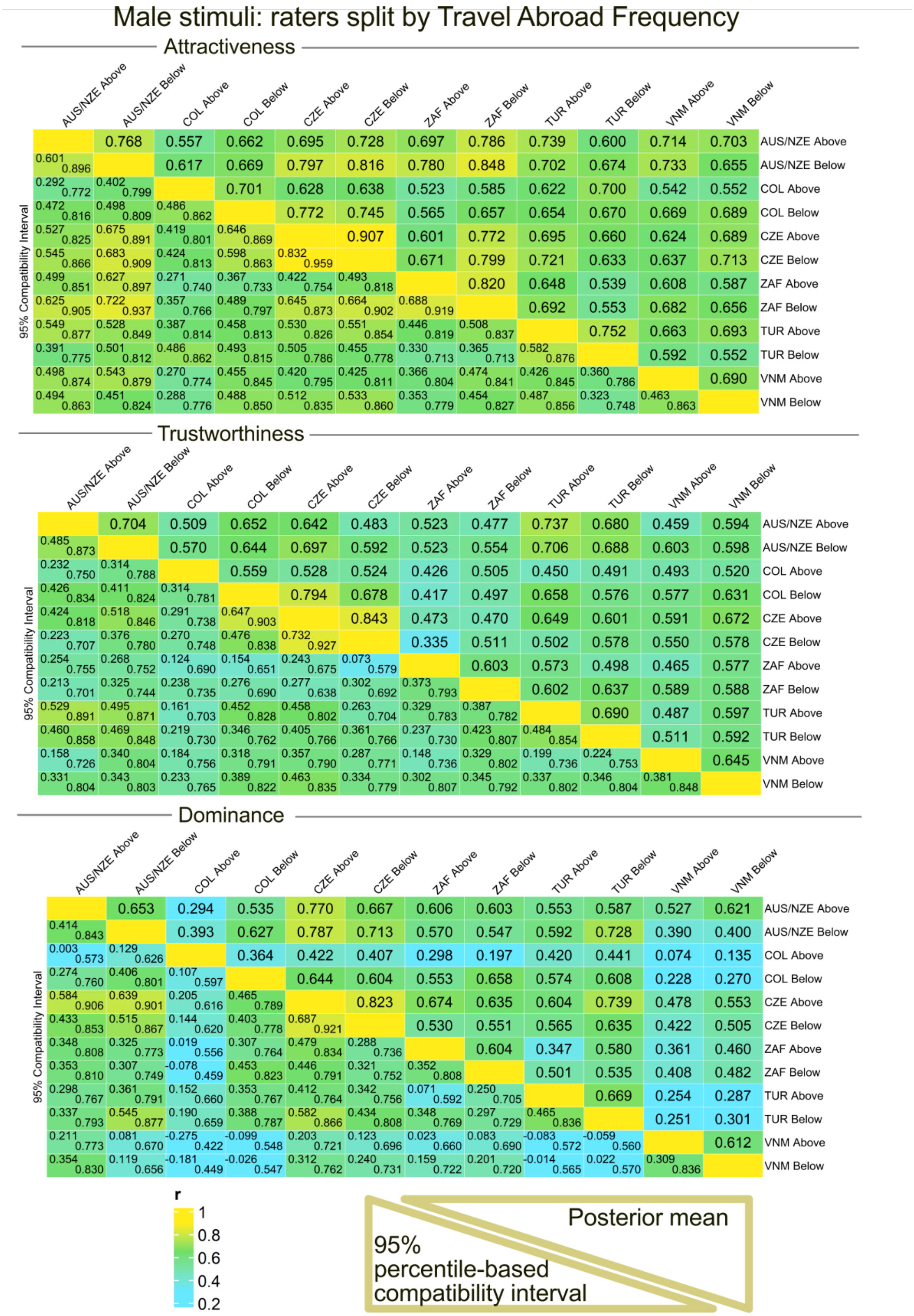

Figure S15. Participants from every country (the abbreviations are same as above), divided according to self-reported frequency of traveling abroad. Male stimuli. Please, mind that the numbers in some samples are low and unequal.

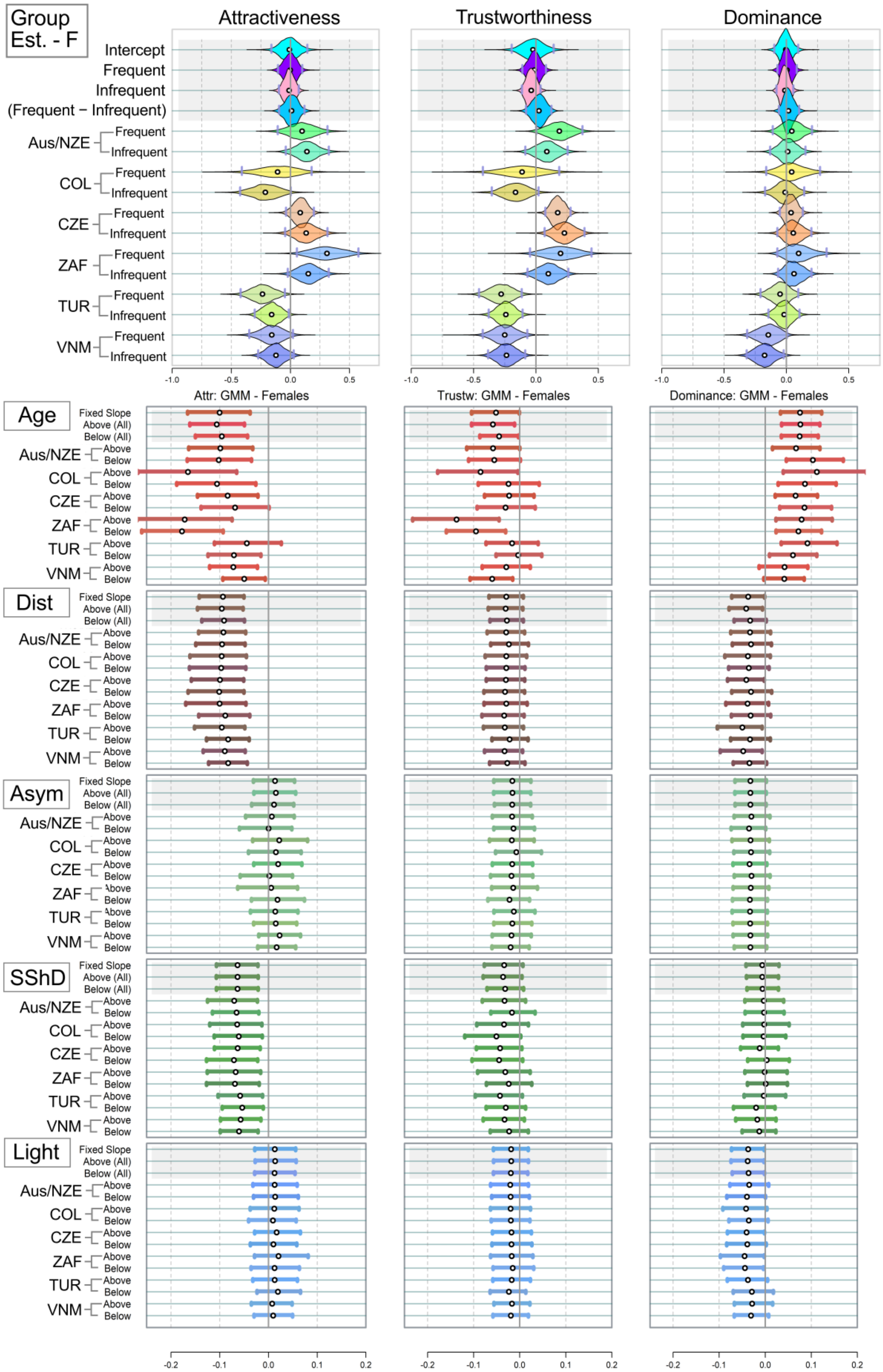

Figure S16. Mean estimated ratings of attractiveness, trustworthiness, and dominance in female stimuli. Analogue of Figure 3, participants divided based on frequency of traveling abroad (*above* = Often, Rather Often, and Occasionally, the rest was put in the category "below").

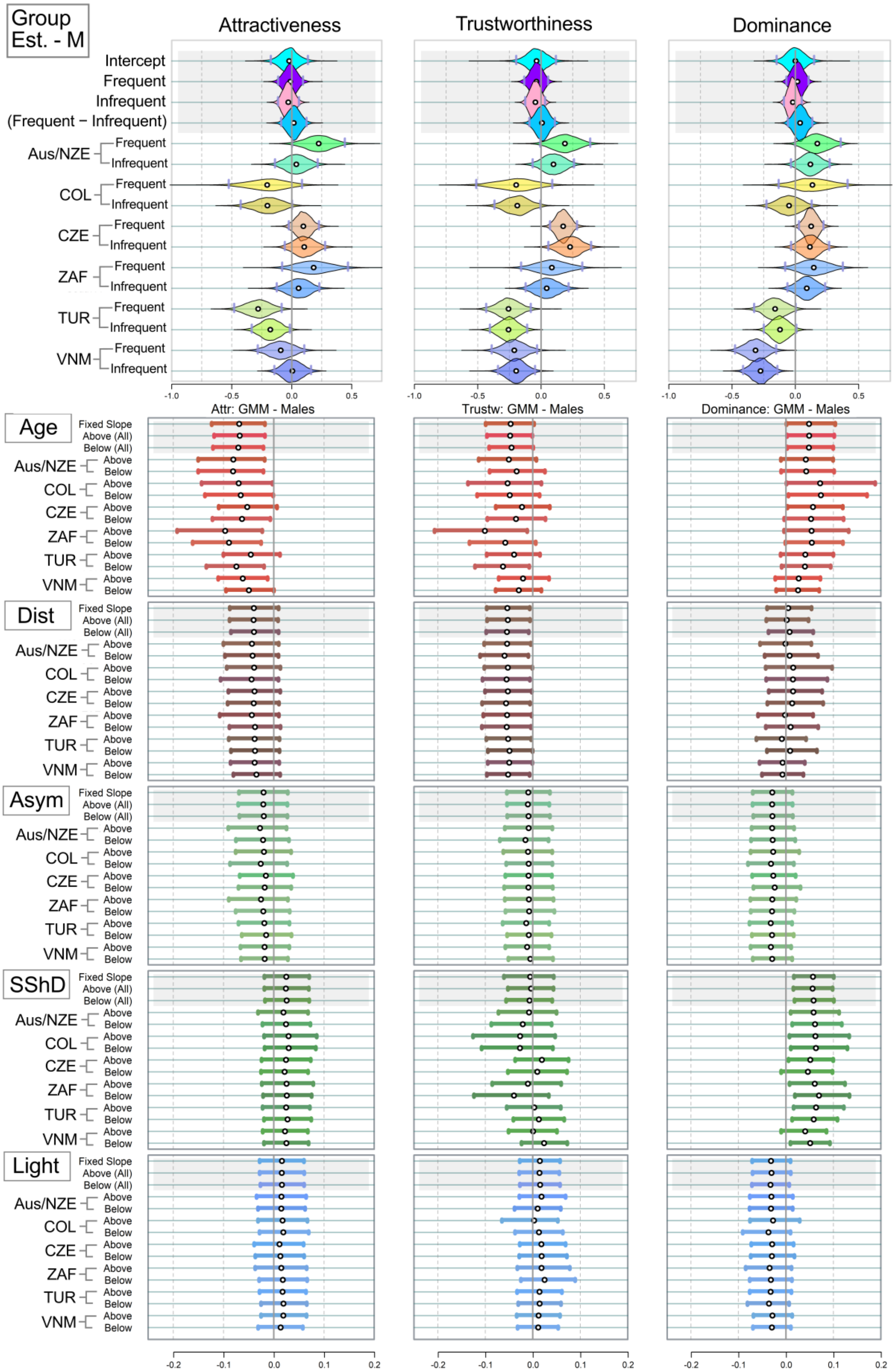

Figure S17. Mean estimated ratings of attractiveness, trustworthiness, and dominance in female stimuli. Analogue of Figure 3, participants divided based on frequency of traveling abroad (*above* = Often, Rather Often, and Occasionally, the rest was put in the category "below").

Table S11. Correlation comparisons. Do those, who travel abroad more frequently (“above”), agree better with each other? Mean stands for “Mean correlation”.

Female stimuli	Attractiveness	Trustworthiness	Dominance
Mean within cultures	0.81 [0.75; 0.86]	0.65 [0.56; 0.73]	0.63 [0.53; 0.72]
Mean across cultures	0.67 [0.61; 0.73]	0.61 [0.54; 0.68]	0.46 [0.38; 0.53]
Difference	*0.14 [0.08; 0.19]*	*0.04 [−0.03; 0.11]*	*0.17 [0.08; 0.26]*
Mean: Above travel users	0.65 [0.57; 0.73]	0.58 [0.48; 0.67]	0.58 [0.48; 0.67]
Mean: Below travel users	0.71 [0.64; 0.77]	0.65 [0.57; 0.73]	0.54 [0.44; 0.63]
Difference	*−0.06 [−0.14; 0.03]*	*−0.07 [−0.18; 0.04]*	*0.04 [−0.09; 0.17]*
Male stimuli	Attractiveness	Trustworthiness	Dominance
Mean within cultures	0.77 [0.70; 0.84]	0.67 [0.58; 0.76]	0.62 [0.53; 0.71]
Mean across cultures	0.67 [0.61; 0.73]	0.57 [0.49; 0.64]	0.50 [0.42; 0.57]
Difference	*0.10 [0.04; 0.17]*	*0.11 [0.02; 0.19]*	*0.13 [0.03; 0.22]*
Mean: Above travel users	0.64 [0.55; 0.72]	0.53 [0.43; 0.63]	0.45 [0.34; 0.55]
Mean: Below travel users	0.69 [0.62; 0.76]	0.60 [0.51; 0.69]	0.54 [0.45; 0.64]
Difference	*−0.05 [−0.15; 0.05]*	*−0.06 [−0.19; 0.06]*	*−0.10 [−0.23; 0.03]*

Part 3.2. Socioeconomic status during childhood – ver. 1

Model layout is still the same, but “above/below” correspond to socioeconomic status.

Socioeconomic status (SES): participants were split in two groups. Participants who reported their Socioeconomic status during childhood as "Rich", "Upper CL" were coded as “above”, the rest of the participants is “below” (NAs and “those who do not want to respond” excluded).

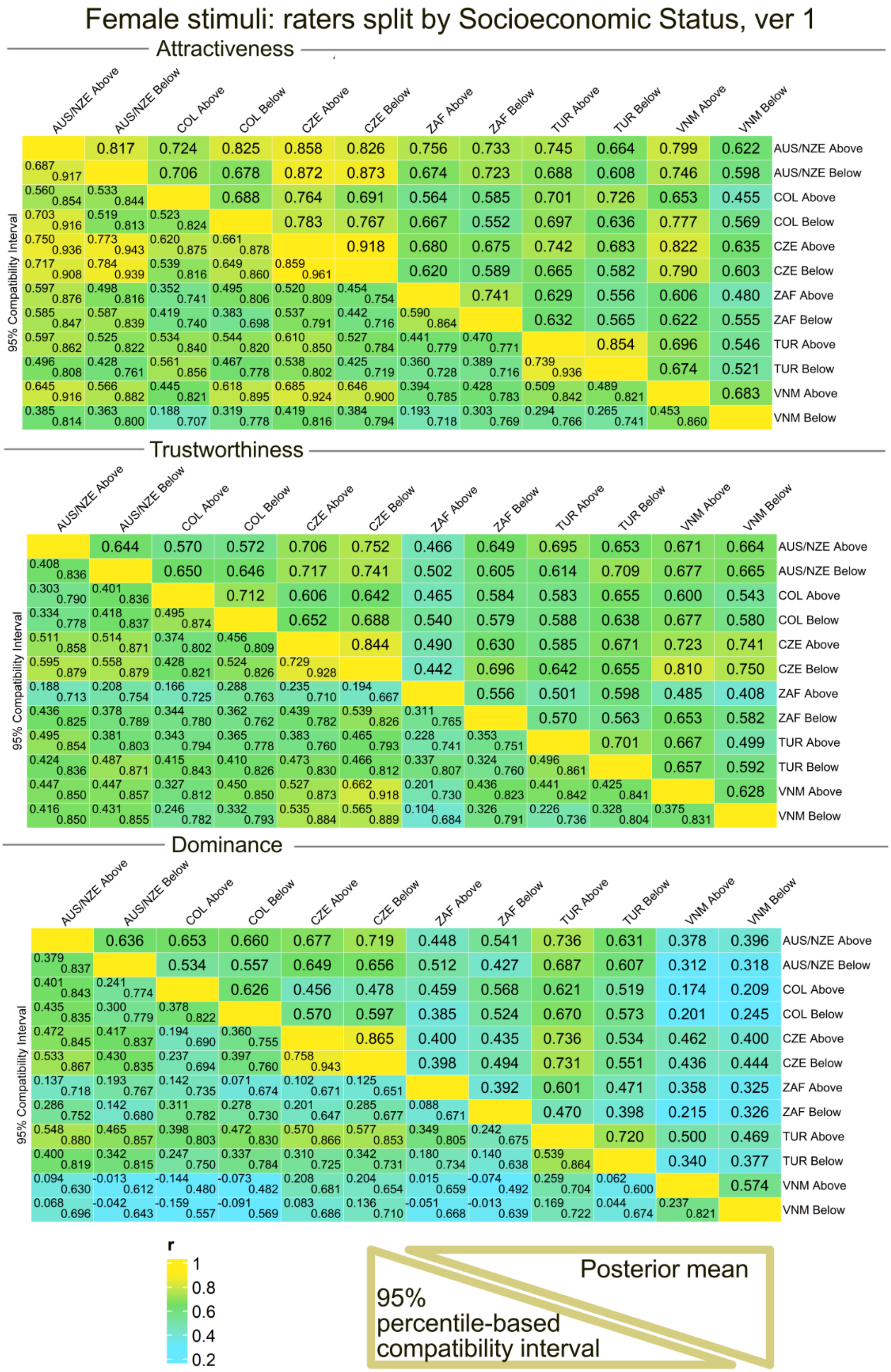

Figure S18. Participants from every country (the abbreviations are same as above), divided according to self-reported SES. Female stimuli. Please, mind that the numbers in some samples are low and unequal / unbalanced. *Version 1*.

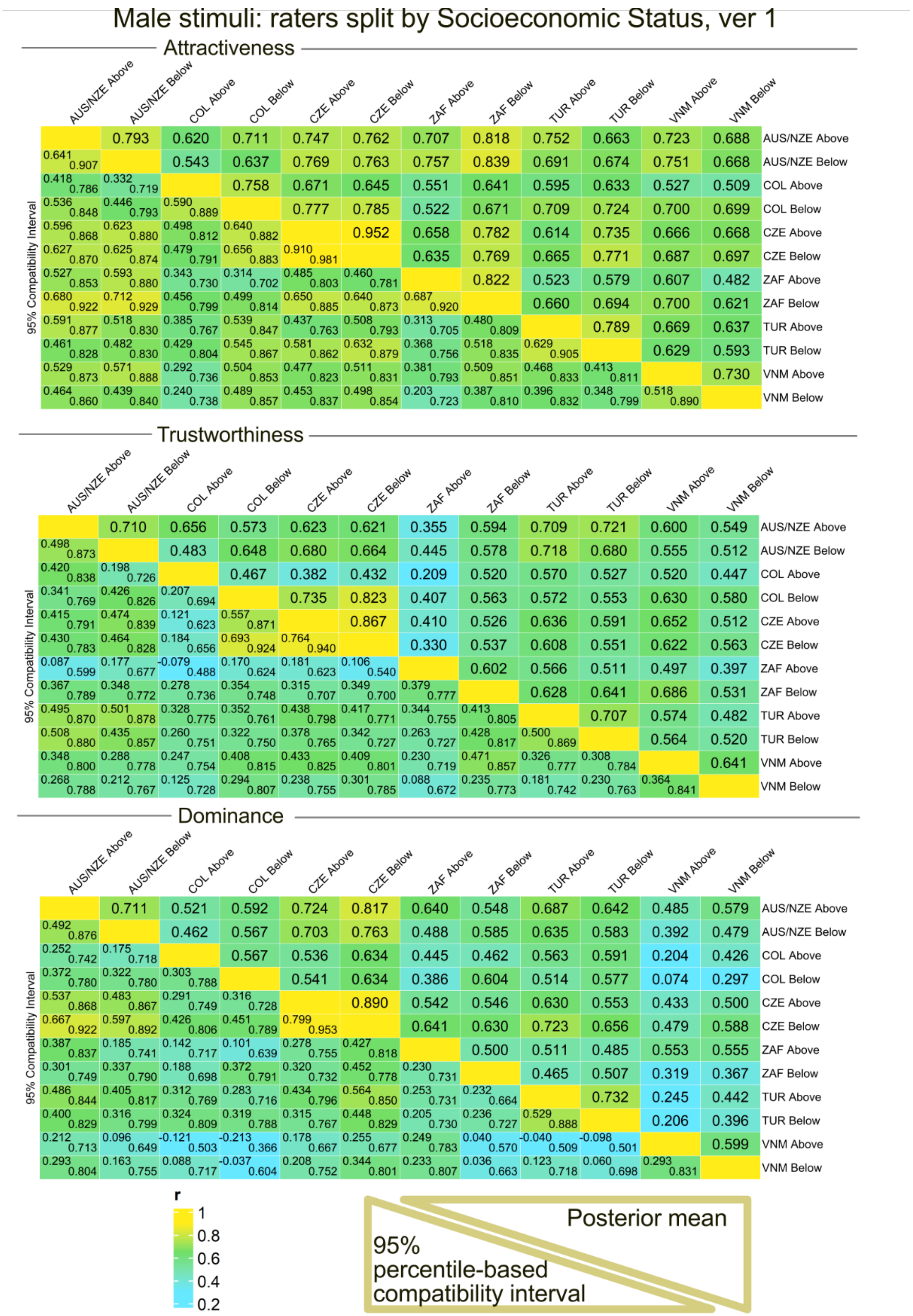

Figure S19. Participants from every country (the abbreviations are same as above), divided according to self-reported SES. Male stimuli. Please, mind that the numbers in some samples are low and unequal / unbalanced. *Version 1*.

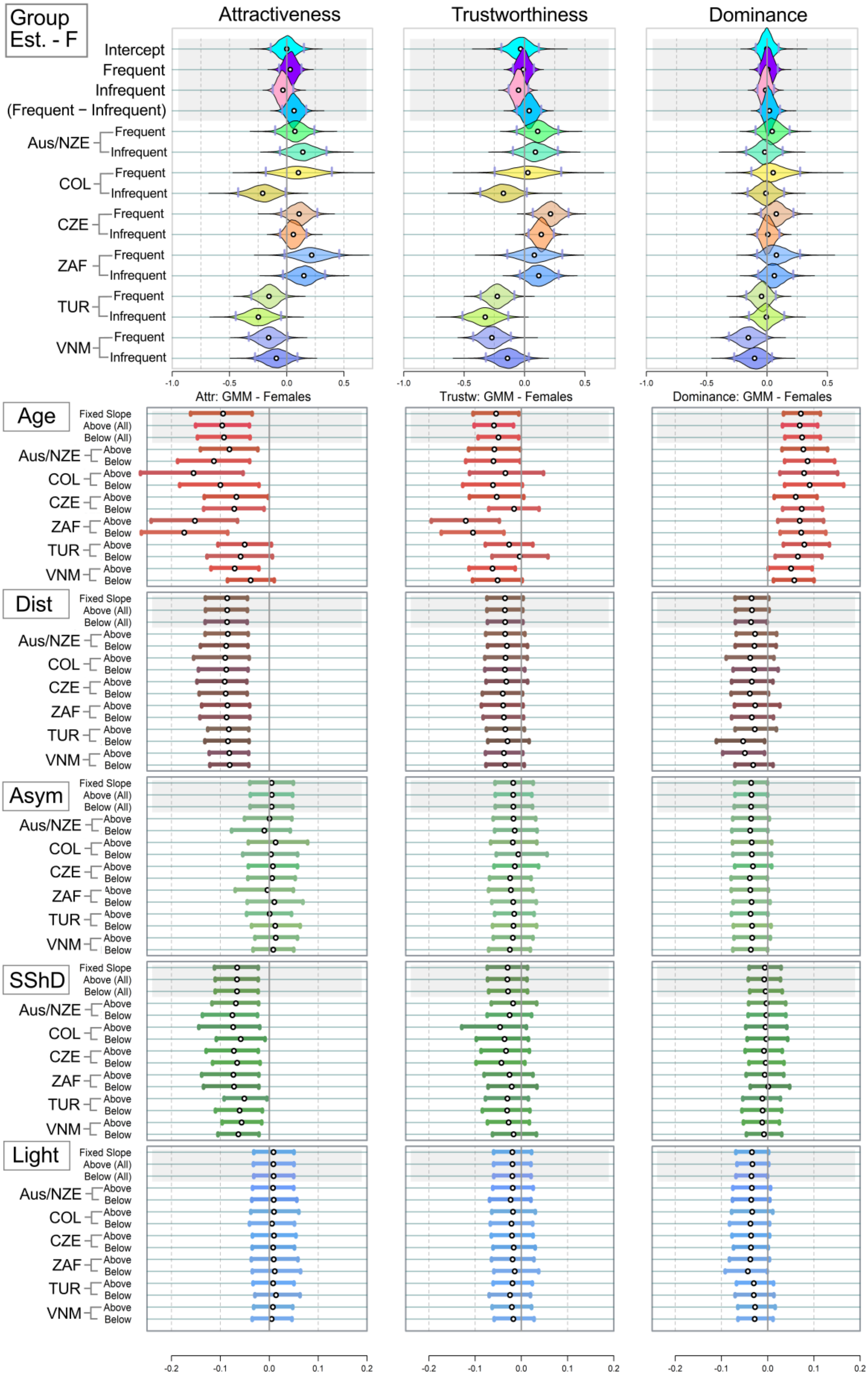

Figure S20. Mean estimated ratings of attractiveness, trustworthiness, and dominance in female stimuli. Analogue of Figure 3, run on models with the three dependent variables (attractiveness, trustworthiness, and dominance), participants divided based on SES. *Version 1*.

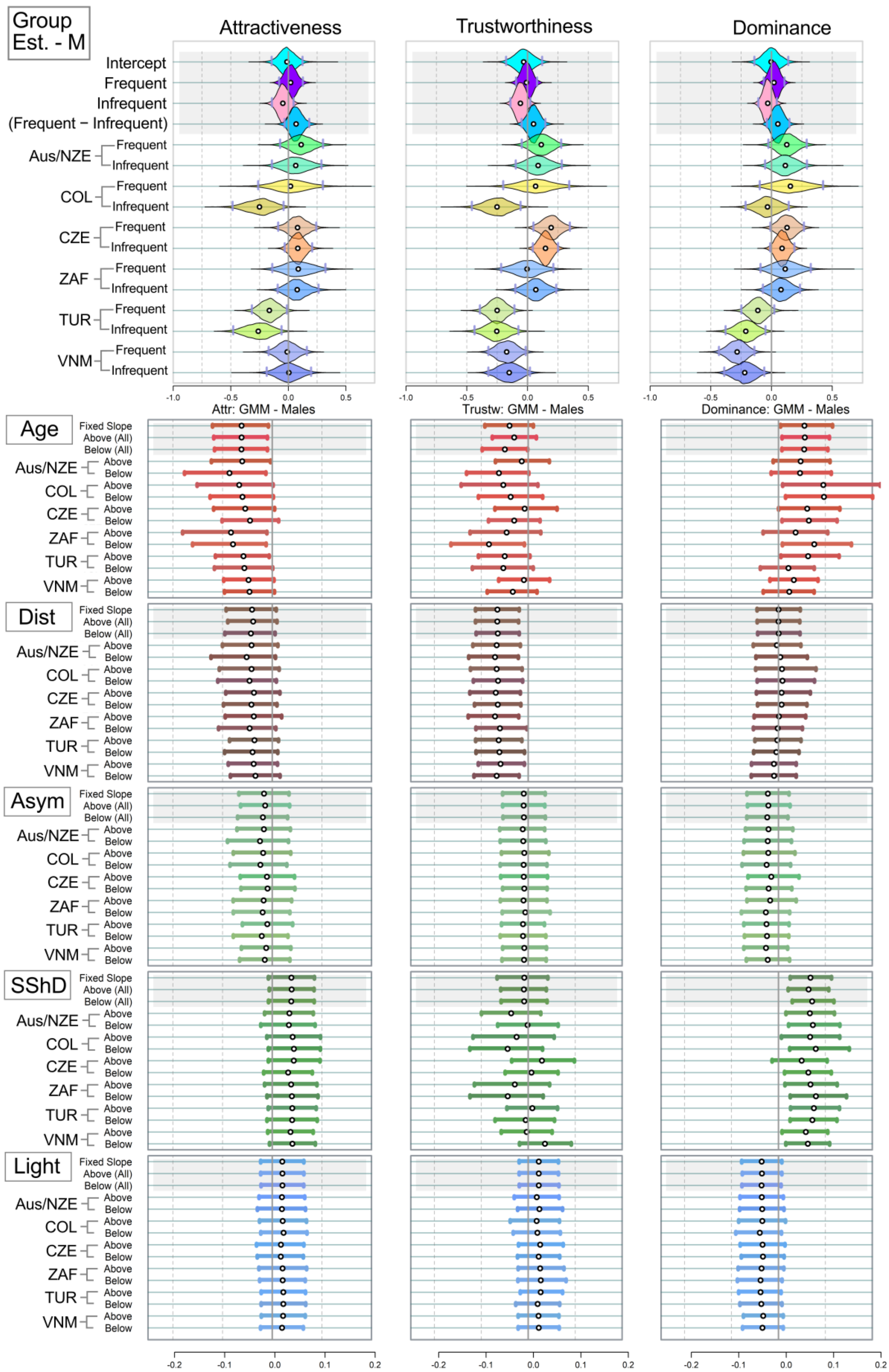

Figure S21. Mean estimated ratings of attractiveness, trustworthiness, and dominance in male stimuli. Analogue of Figure 3, run on models with the three dependent variables (attractiveness, trustworthiness, and dominance), participants divided based on SES. *Version 1*.

Table S12. Correlation comparisons. Do those, who came from high-SES families, agree better with each other? Mean stands for “Mean correlation”. Version 1.

Female stimuli	Attractiveness	Trustworthiness	Dominance
Mean within cultures	0.78 [0.72; 0.84]	0.68 [0.59; 0.76]	0.64 [0.54; 0.73]
Mean across cultures	0.68 [0.62; 0.73]	0.62 [0.55; 0.69]	0.48 [0.41; 0.56]
Difference	*0.11 [0.05; 0.17]*	*0.06 [−0.02; 0.13]*	*0.15 [0.06; 0.25]*
Mean: SES Above	0.72 [0.64; 0.78]	0.59 [0.49; 0.68]	0.59 [0.49; 0.68]
Mean: SES Below	0.63 [0.55; 0.71]	0.65 [0.56; 0.73]	0.47 [0.37; 0.58]
Difference	*0.09 [−0.00; 0.18]*	*−0.06 [−0.17; 0.06]*	*0.11 [−0.03; 0.25]*
Male stimuli	Attractiveness	Trustworthiness	Dominance
Mean within cultures	0.81 [0.75; 0.86]	0.67 [0.58; 0.75]	0.67 [0.57; 0.75]
Mean across cultures	0.67 [0.61; 0.73]	0.56 [0.48; 0.63]	0.51 [0.43; 0.59]
Difference	*0.14 [0.08; 0.20]*	*0.11 [0.03; 0.19]*	*0.15 [0.06; 0.25]*
Mean: SES Above	0.64 [0.56; 0.72]	0.53 [0.43; 0.62]	0.51 [0.41; 0.62]
Mean: SES Below	0.71 [0.63; 0.78]	0.60 [0.50; 0.69]	0.55 [0.45; 0.65]
Difference	*−0.07 [−0.16; 0.03]*	*−0.07 [−0.19; 0.05]*	*−0.03 [−0.17; 0.10]*

Part 3.3. Socioeconomic status during childhood – ver. 2

Model layout is still the same, “above/below” correspond to socioeconomic status as before. **Socioeconomic status**: participants were split in two groups. Participants who reported their Socioeconomic Status during childhood as "Rich", "Upper CL", **and “Middle”** were coded as “above”, the rest of the participants is “below” (NAs and “those who do not want to respond” excluded).

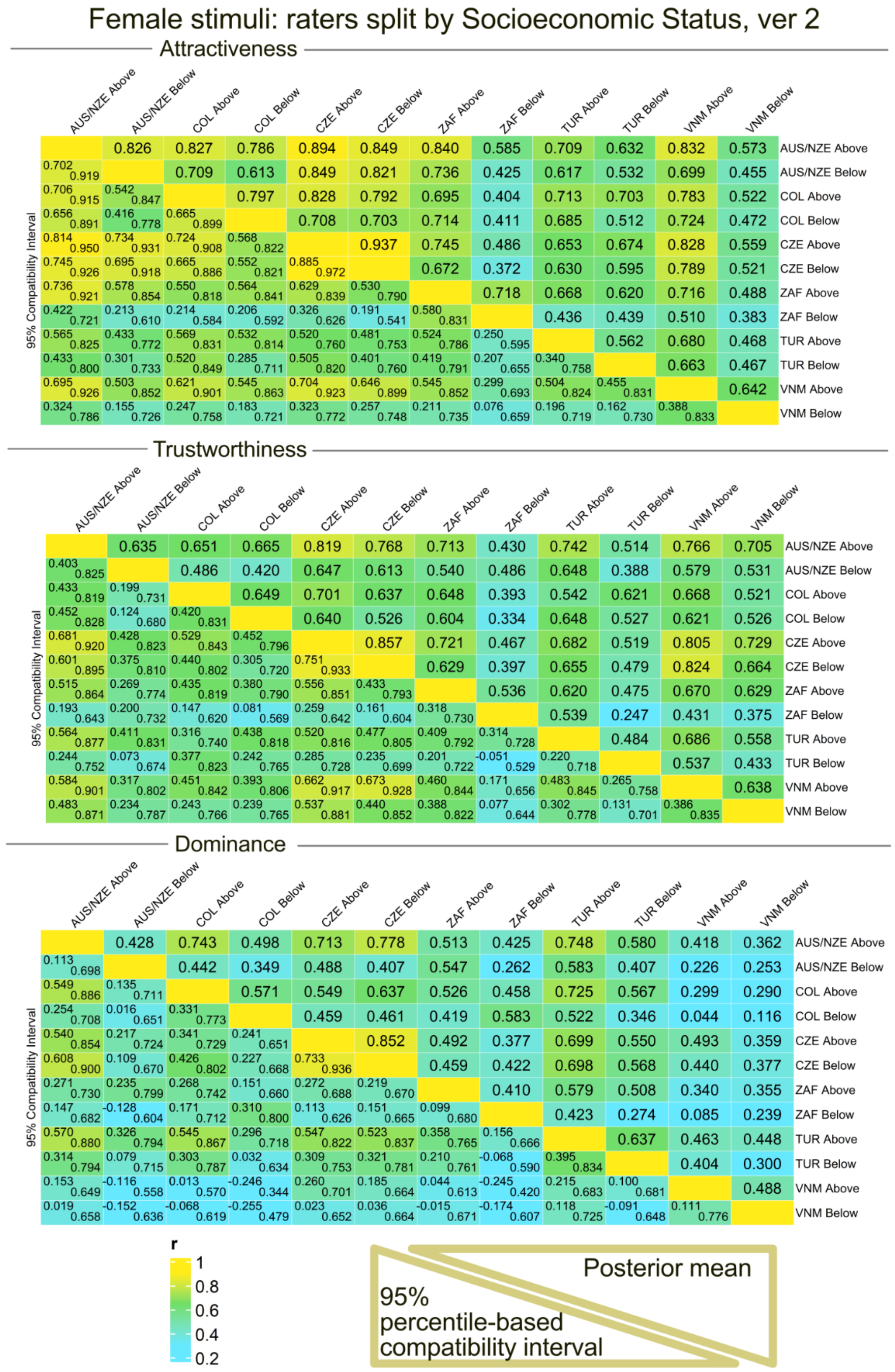

Figure S22. Participants from every country (the abbreviations are same as above), divided according to self-reported SES. Female stimuli. Please, mind that the numbers in some samples are low and, therefore, samples are heavily unbalanced. *Version 2*.

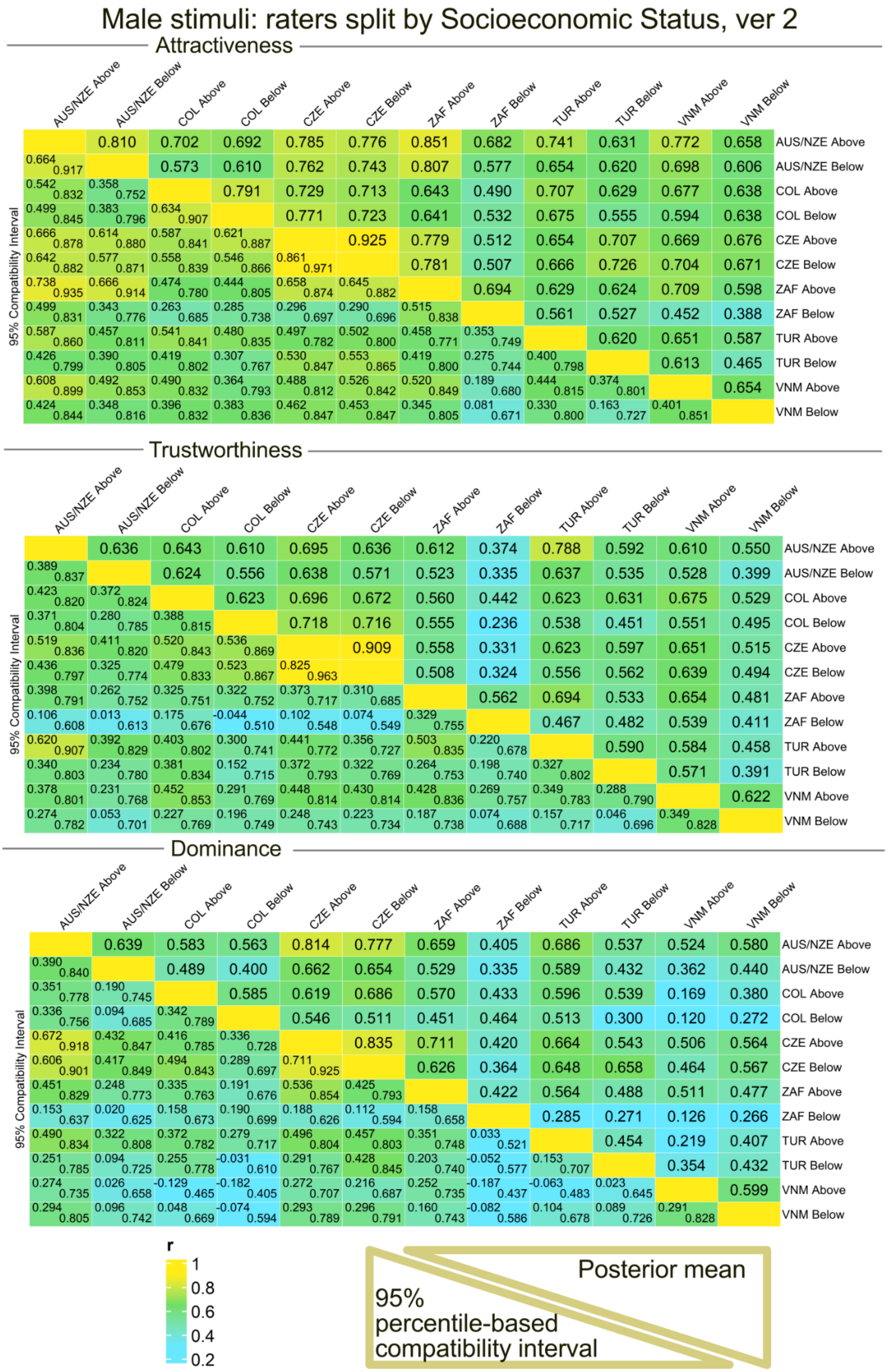

Figure S23. Participants from every country (the abbreviations are same as above), divided according to SES. Male stimuli. Please, mind that the numbers in some samples are low and unequal / unbalanced. *Version 2*.

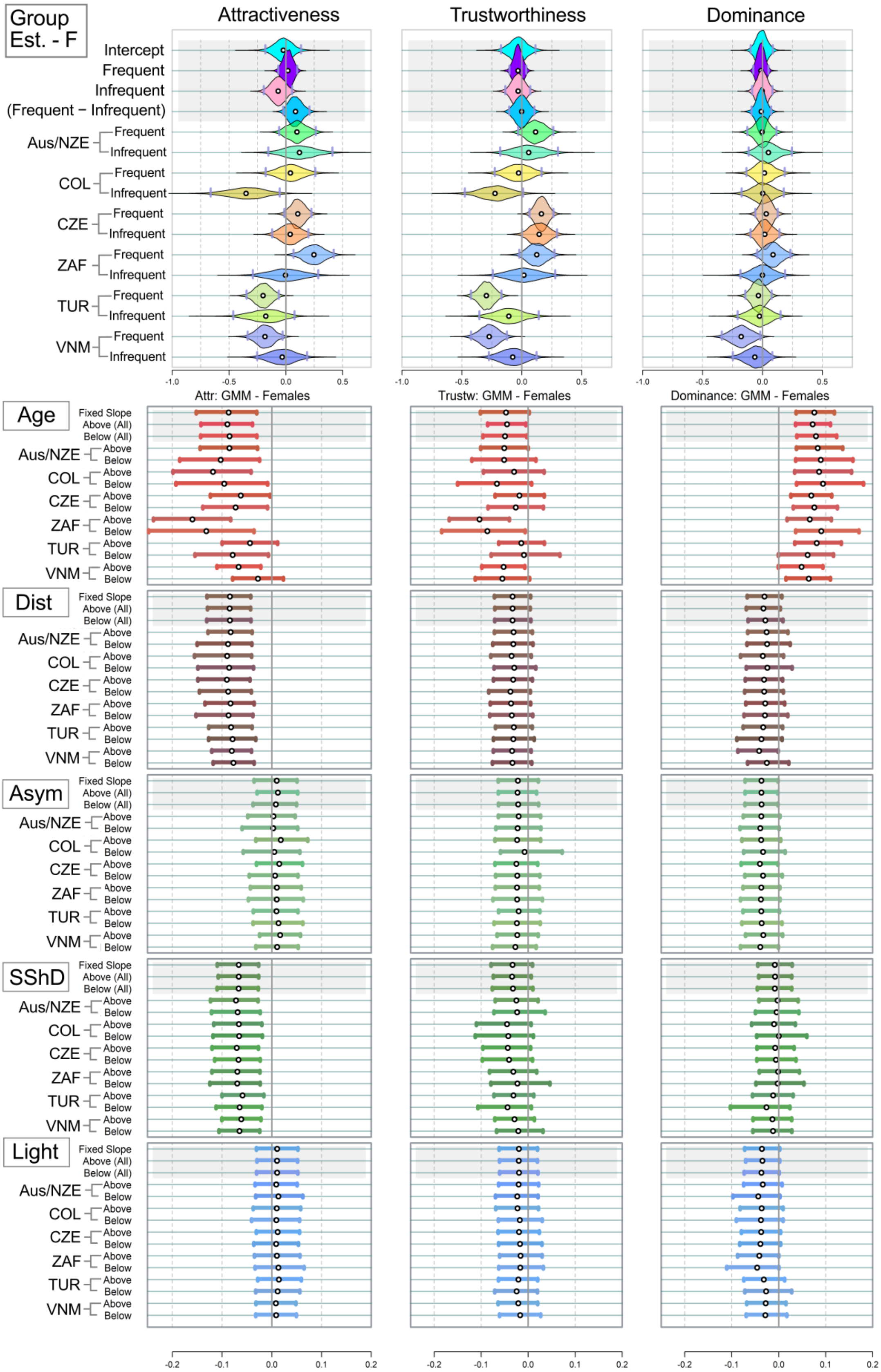

Figure S24. Mean estimated ratings of attractiveness, trustworthiness, and dominance in female stimuli. Analogue of Figure 3, run on models with the three dependent variables (attractiveness, trustworthiness, and dominance), participants divided based on SES (three groups above). *Version 2*.

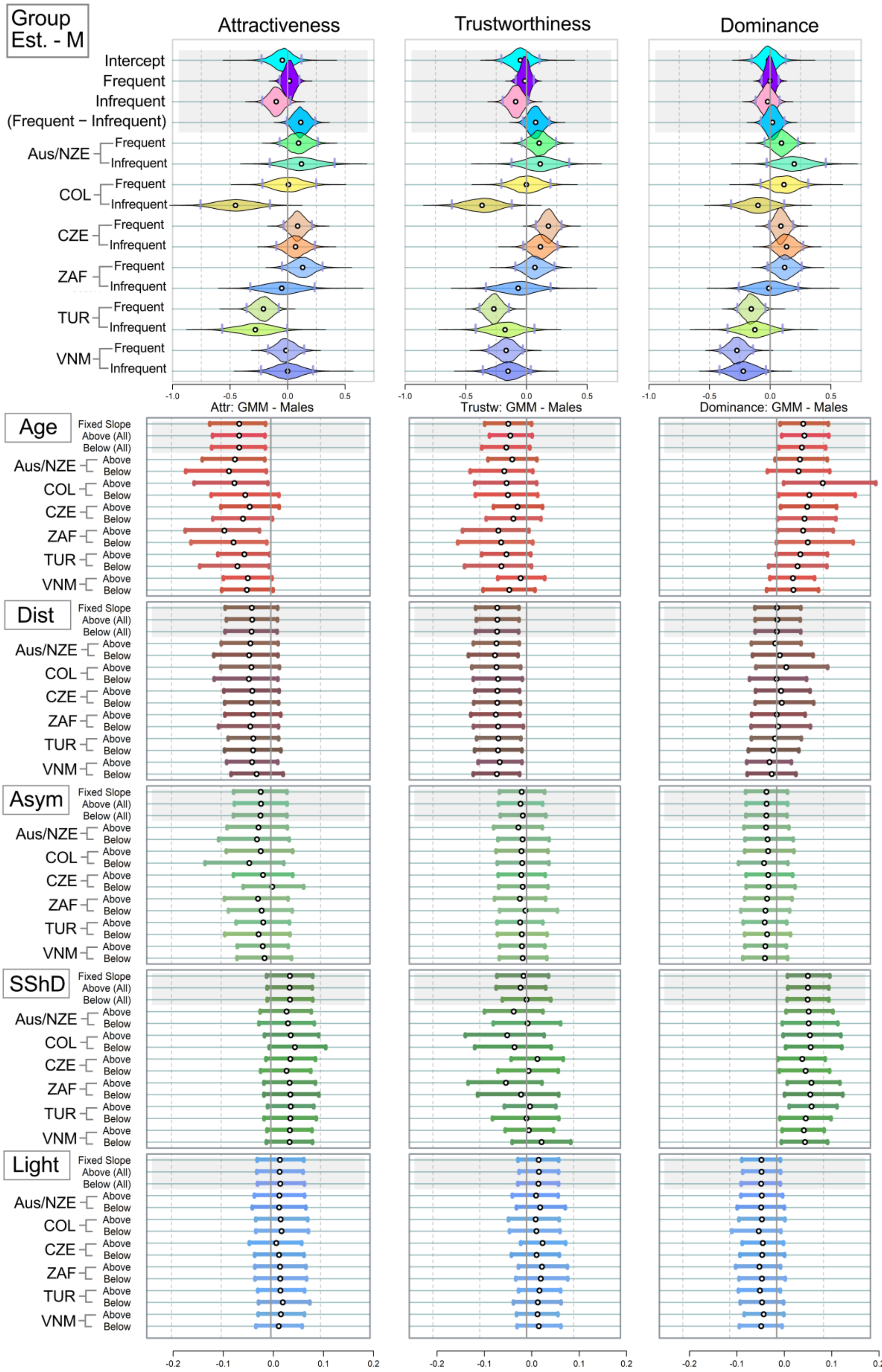

Figure S25. Mean estimated ratings of attractiveness, trustworthiness, and dominance in male stimuli. Analogue of Figure 3, run on models with the three dependent variables (attractiveness or trustworthiness or dominance), participants divided based on SES. *Version 2*.

Table S13. Correlation comparisons. Do those, who came from higher-SES families, agree better with each other? Mean stands for "Mean correlation". Version 1.

Female stimuli	Attractiveness	Trustworthiness	Dominance
Mean within cultures	0.75 [0.68; 0.81]	0.63 [0.54; 0.72]	0.56 [0.46; 0.67]
Mean across cultures	0.64 [0.58; 0.70]	0.59 [0.52; 0.66]	0.45 [0.37; 0.53]
Difference	*0.10 [0.05; 0.17]*	*0.04 [−0.03; 0.12]*	*0.12 [0.02; 0.22]*
Mean: SES Above users	0.76 [0.70; 0.82]	0.70 [0.61; 0.76]	0.70 [0.61; 0.77]
Mean: SES Below users	0.51 [0.42; 0.60]	0.46 [0.36; 0.56]	0.36 [0.24; 0.48]
Difference	*0.25 [0.15; 0.34]*	*0.23 [0.12; 0.35]*	*0.34 [0.19; 0.48]*
Male stimuli	Attractiveness	Trustworthiness	Dominance
Mean within cultures	0.75 [0.68; 0.81]	0.66 [0.57; 0.74]	0.59 [0.49; 0.68]
Mean across cultures	0.65 [0.62; 0.69]	0.55 [0.51; 0.60]	0.49 [0.44; 0.53]
Difference	*0.10 [0.02; 0.17]*	*0.10 [0.00; 0.20]*	*0.10 [−0.00; 0.21]*
Mean: SES Above users	0.71 [0.67; 0.75]	0.64 [0.59; 0.69]	0.56 [0.50; 0.61]
Mean: SES Below users	0.59 [0.53; 0.65]	0.47 [0.39; 0.54]	0.42 [0.35; 0.50]
Difference	*0.12 [0.05; 0.19]*	*0.18 [0.09; 0.26]*	*0.14 [0.05; 0.23]*

Explanation of the large effect in Table S8: When we put the group "Middle" to the "Above" group, the group "Below" becomes much smaller than the "Above" group. We consider these estimates, and, too, differences between groups, unreliable. This is not an instance when Bayesian multi-level modelling with partial pooling could help. Nevertheless, the agreement being higher between more affluent groups would make a perfect sense considering previous literature (e.g., Batres & Perrett, 2014). The only problem is that all our participants have access to the internet (i.e., all were, in our opinion, on the same side of the digital divide).

Part VI: Score based on 3 visually oriented SM

Identifying the underlying factor – based on the three variables below – frequency of visiting YouTube, Instagram, and TikTok:

```
    Freq_YT          Freq_INS          Freq_TT     
 Min.   :1.000   Min.   :1.000   Min.   :1.000  
 1st Qu.:3.000   1st Qu.:3.000   1st Qu.:1.000  
 Median :4.000   Median :5.000   Median :2.000  
 Mean   :4.076   Mean   :4.297   Mean   :2.943  
 3rd Qu.:5.000   3rd Qu.:6.000   3rd Qu.:5.000  
 Max.   :6.000   Max.   :6.000   Max.   :6.000  
```

This one we use:

```
> efa1_3_ml <- fa(r = rho, nfactors = 1, fm = "ml", rotate = "oblimin")
>
```

```
> print(efa1_3_ml$loadings, cutoff=.10)

Loadings:
         ML1  
Freq_YT  0.283
Freq_INS 0.726
Freq_TT  0.708

                 ML1
SS loadings    1.109
Proportion Var 0.370
```

This one we don't:

```
> efa1_2_ml <- fa(r = rho, nfactors = 1, fm = "ml", rotate = "oblimin")
>
> print(efa1_2_ml$loadings, cutoff=.10)

Loadings:
         ML1  
Freq_INS 0.717
Freq_TT  0.717

                 ML1
SS loadings    1.029
Proportion Var 0.514
```

But their Pearson correlation is 0.97, so it's not unimportant.

(the layout of the model is the very same as for the main models, reported in the manuscript)

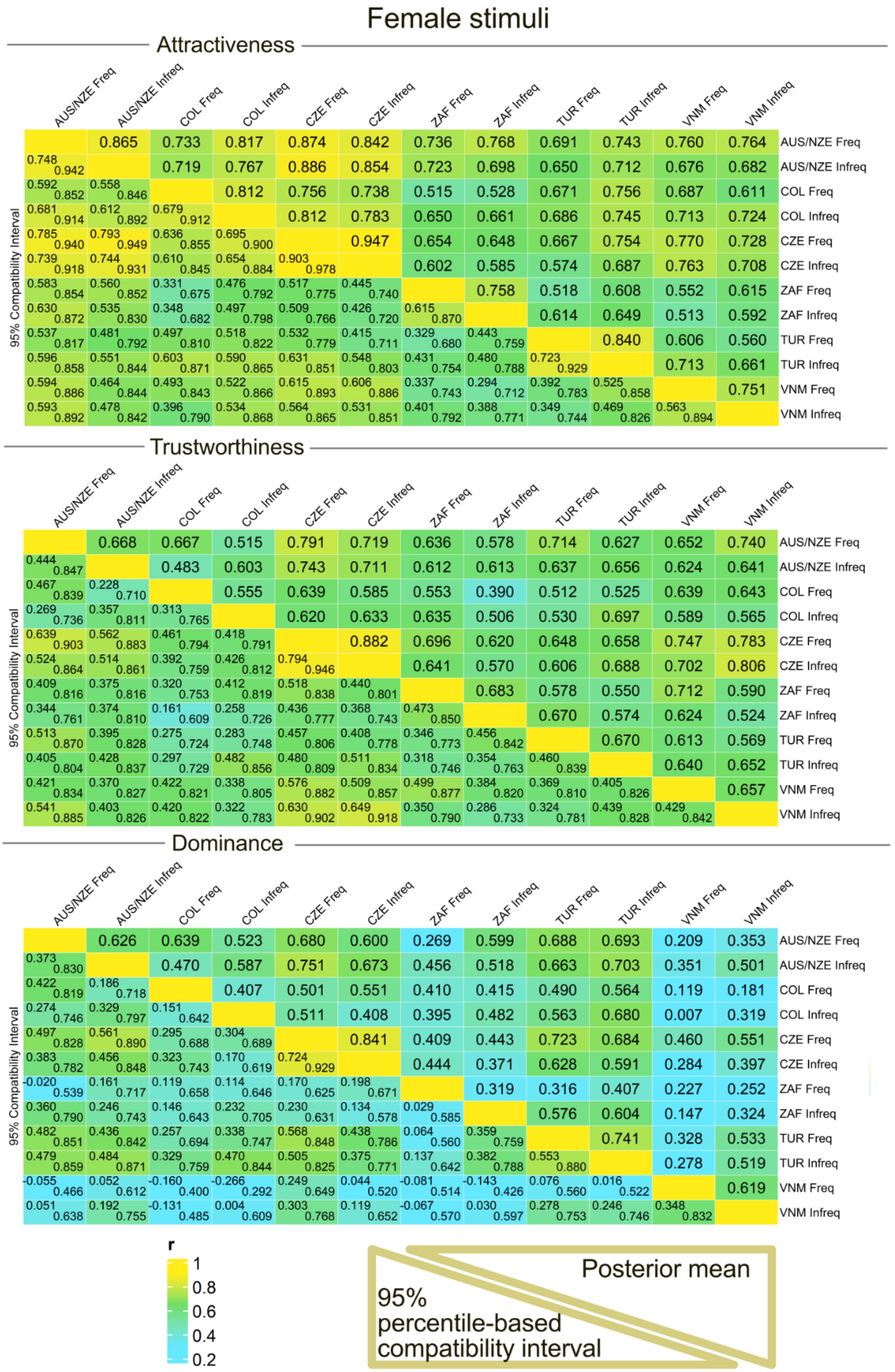

Figure S26. Participants from every country (the abbreviations are same as above), divided according to underlying factor based on frequency of using of three visually oriented social media (YouTube, Instagram, and TikTok). Female stimuli. Please, mind that the numbers in some samples are low and, therefore, samples are heavily unbalanced.

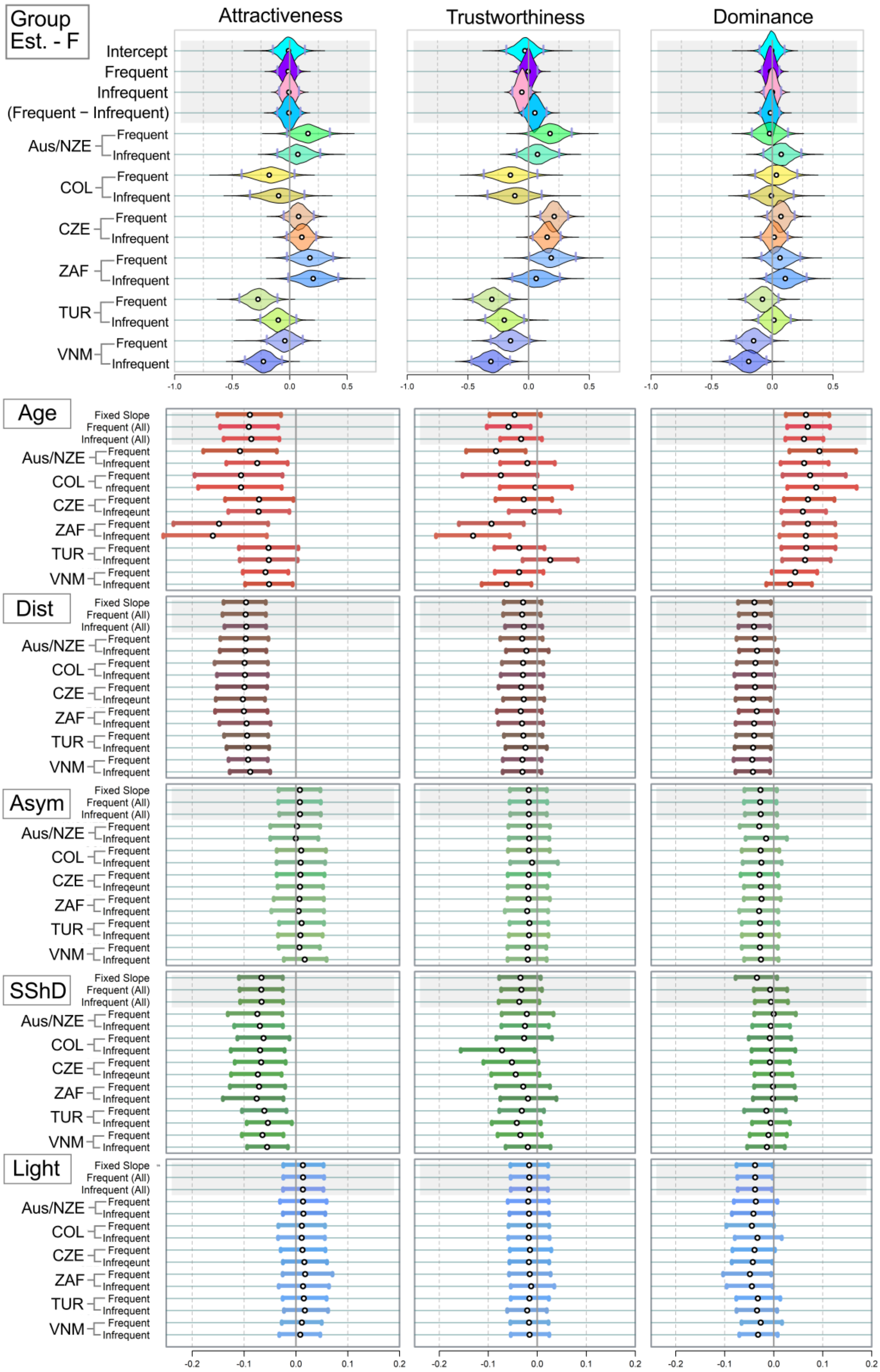

Figure S27. Mean estimated ratings of attractiveness, trustworthiness, and dominance in female stimuli. Analogue of Figure 3, run on models with the three dependent variables (attractiveness, trustworthiness, and dominance), participants divided based on the underlying factor based on frequency of using of three visually oriented social media.

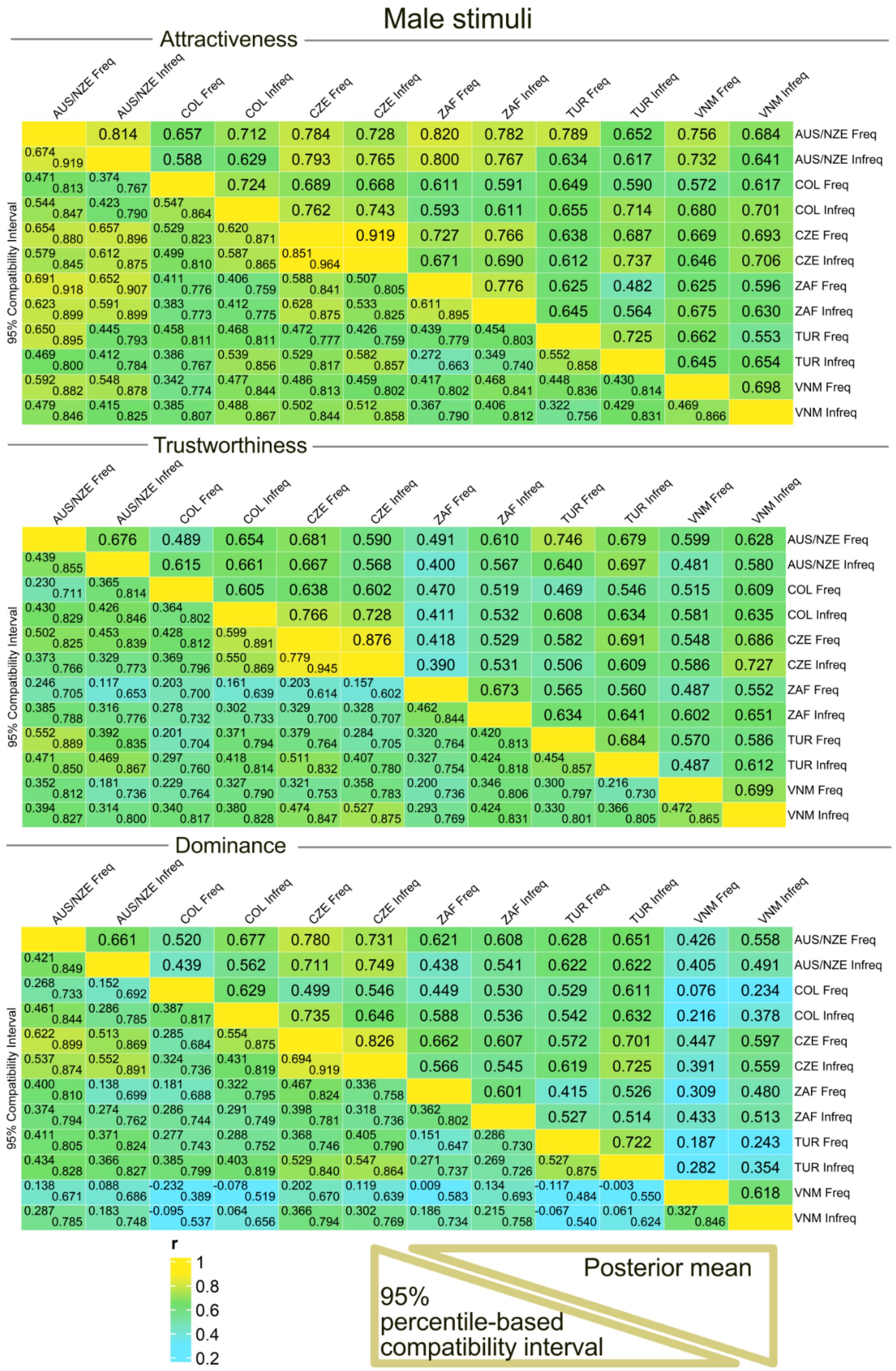

Figure S28. Participants from every country (the abbreviations are same as above), divided according to underlying factor based on frequency of using of three visually oriented social media (YouTube, Instagram, and TikTok). Male stimuli. Please, mind that the numbers in some samples are low and, therefore, samples are heavily unbalanced.

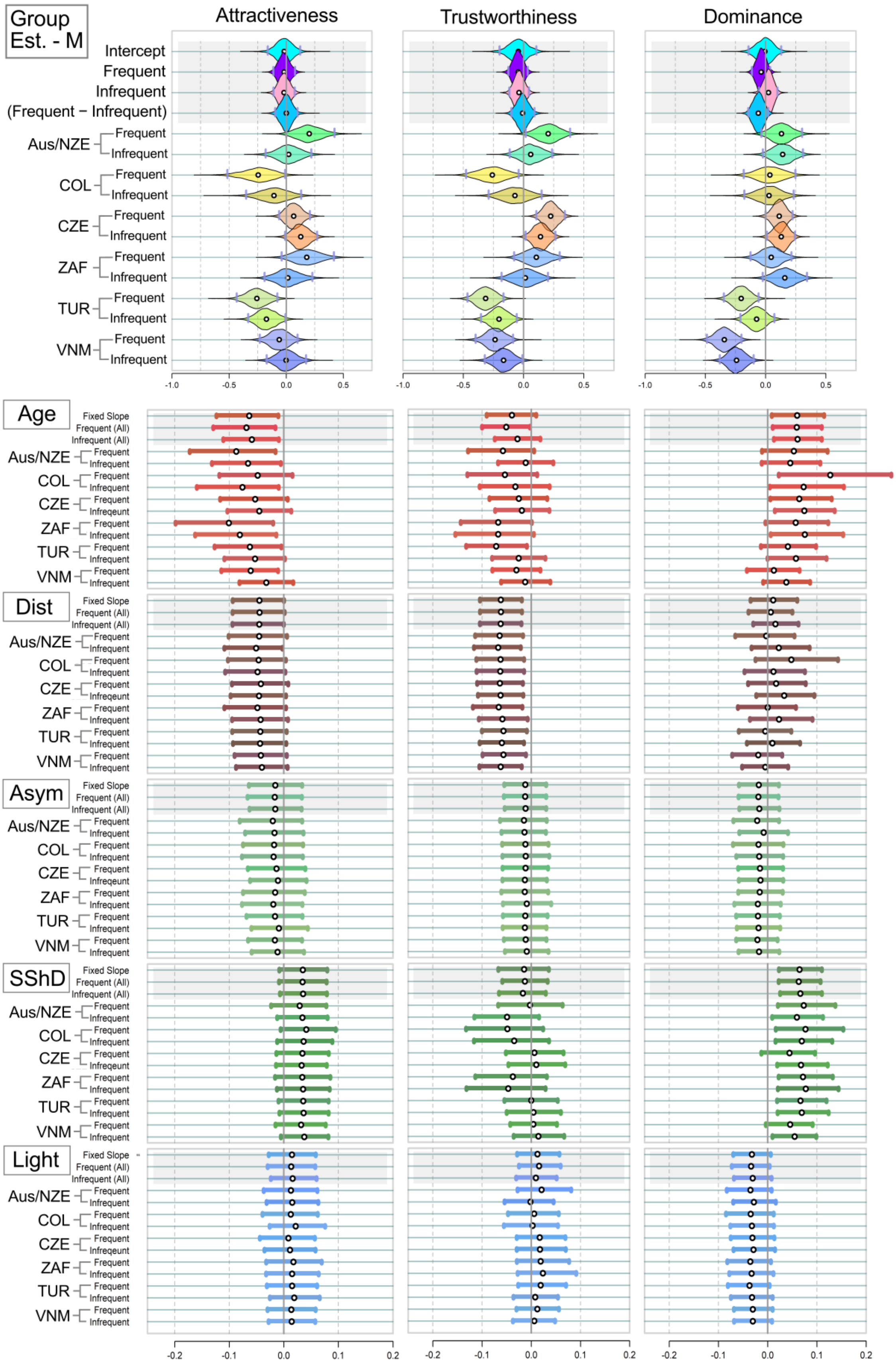

Figure S29. Mean estimated ratings of attractiveness, trustworthiness, and dominance in female stimuli. Analogue of Figure 3, run on models with the three dependent variables (attractiveness, trustworthiness, and dominance), participants divided based on the underlying factor based on frequency of using of three visually oriented social media.

Table S14. Correlation comparisons. Mean stands for "Mean correlation". Median split based on the three visually oriented social media.

Female stimuli	Attractiveness	Trustworthiness	Dominance
Mean within cultures	0.83 [0.78; 0.88]	0.69 [0.60; 0.76]	0.59 [0.49; 0.68]
Mean across cultures	0.69 [0.64; 0.74]	0.62 [0.54; 0.68]	0.46 [0.39; 0.54]
Difference	*0.14 [0.08; 0.19]*	*0.07 [0.00; 0.14]*	*0.13 [0.04; 0.22]*
Mean: Frequent users	0.68 [0.61; 0.75]	0.65 [0.57; 0.73]	0.65 [0.57; 0.74]
Mean: Infrequent users	0.70 [0.63; 0.77]	0.63 [0.54; 0.71]	0.51 [0.42; 0.61]
Difference	*−0.02 [−0.11; 0.06]*	*−0.02 [−0.09; 0.13]*	*0.14 [0.01; 0.27]*
**Male stimuli**	**Attractiveness**	**Trustworthiness**	**Dominance**
Mean within cultures	0.77 [0.71; 0.83]	0.70 [0.62; 0.78]	0.68 [0.58; 0.76]
Mean across cultures	0.66 [0.60; 0.72]	0.58 [0.51; 0.65]	0.53 [0.45; 0.60]
Difference	*0.11 [0.05; 0.18]*	*0.12 [0.04; 0.20]*	*0.15 [0.05; 0.24]*
Mean: Frequent users	0.68 [0.61; 0.76]	0.55 [0.46; 0.64]	0.47 [0.38; 0.57]
Mean: Infrequent users	0.68 [0.60; 0.75]	0.62 [0.53; 0.71]	0.56 [0.46; 0.65]
Difference	*0.01 [−0.09; 0.10]*	*−0.07 [−0.19; −0.04]*	*−0.08 [−0.20; 0.04]*

Commentary: We observe the usual pattern – agreement is always substantial, more so within cultures (samples from the same country). Frequency of social media use has only limited effects; however, stability frequent users agree with each other more in perceived female dominance. This is definitely an effect that would deserve further testing, was it observed in some more stable environment than is the internet. In this case, even though dominance may be somehow important characteristic on the Internet, and perhaps more so in women, to enforce, this is the only prediction we dare to make. Mind that the Internet rapidly changes and our data, collected mostly in the 2nd half of 2024, are mostly outdated.

References

Batres C, Perrett DI (2014) The Influence of the Digital Divide on Face Preferences in El Salvador: People without Internet Access Prefer More Feminine Men, More Masculine Women, and Women with Higher Adiposity. PLoS ONE 9(7): e100966. doi:10.1371/journal.pone.0100966

Rosseel Y (2012). "lavaan: An R Package for Structural Equation Modeling." Journal of Statistical Software, 48(2), 1–36. doi:10.18637/jss.v048.i02.
